# The Intolerance of Regulatory Sequence to Genetic Variation Predicts Gene Dosage Sensitivity

**DOI:** 10.1371/journal.pgen.1005492

**Published:** 2015-09-02

**Authors:** Slavé Petrovski, Ayal B. Gussow, Quanli Wang, Matt Halvorsen, Yujun Han, William H. Weir, Andrew S. Allen, David B. Goldstein

**Affiliations:** 1 Institute for Genomic Medicine, Columbia University, New York, New York, United States of America; 2 Center for Human Genome Variation, Duke University, School of Medicine, Durham, North Carolina, United States of America; 3 Department of Medicine, The University of Melbourne, Austin Health and Royal Melbourne Hospital, Melbourne, Victoria, Australia; 4 Program in Computational Biology and Bioinformatics, Duke University, Durham, North Carolina, United States of America; 5 Department of Biostatistics and Bioinformatics, Duke University, Durham, North Carolina, United States of America; Yale School of Medicine, UNITED STATES

## Abstract

Noncoding sequence contains pathogenic mutations. Yet, compared with mutations in protein-coding sequence, pathogenic regulatory mutations are notoriously difficult to recognize. Most fundamentally, we are not yet adept at recognizing the sequence stretches in the human genome that are most important in regulating the expression of genes. For this reason, it is difficult to apply to the regulatory regions the same kinds of analytical paradigms that are being successfully applied to identify mutations among protein-coding regions that influence risk. To determine whether dosage sensitive genes have distinct patterns among their noncoding sequence, we present two primary approaches that focus solely on a gene’s proximal noncoding regulatory sequence. The first approach is a regulatory sequence analogue of the recently introduced residual variation intolerance score (RVIS), termed noncoding RVIS, or ncRVIS. The ncRVIS compares observed and predicted levels of standing variation in the regulatory sequence of human genes. The second approach, termed ncGERP, reflects the phylogenetic conservation of a gene’s regulatory sequence using GERP++. We assess how well these two approaches correlate with four gene lists that use different ways to identify genes known or likely to cause disease through changes in expression: 1) genes that are known to cause disease through haploinsufficiency, 2) genes curated as dosage sensitive in ClinGen’s Genome Dosage Map, 3) genes judged likely to be under purifying selection for mutations that change expression levels because they are statistically depleted of loss-of-function variants in the general population, and 4) genes judged unlikely to cause disease based on the presence of copy number variants in the general population. We find that both noncoding scores are highly predictive of dosage sensitivity using any of these criteria. In a similar way to ncGERP, we assess two ensemble-based predictors of regional noncoding importance, ncCADD and ncGWAVA, and find both scores are significantly predictive of human dosage sensitive genes and appear to carry information beyond conservation, as assessed by ncGERP. These results highlight that the intolerance of noncoding sequence stretches in the human genome can provide a critical complementary tool to other genome annotation approaches to help identify the parts of the human genome increasingly likely to harbor mutations that influence risk of disease.

## Author Summary

Mutations in noncoding sequence can cause disease but are very difficult to recognize. Here, we present two approaches intended to help identify noncoding regions of the genome that may carry mutations influencing disease. The first approach is based on comparing observed and predicted levels of standing human variation in the noncoding exome sequence of a gene. The second approach is based on the phylogenetic conservation of a gene’s noncoding exome sequence using GERP++. We find that both approaches can predict genes known to cause disease through changes in expression level, genes depleted of loss-of-function alleles in the general population, and genes permissive of copy number variants in the general population. We find that both scores aid in interpreting loss-of-function mutations and in defining regions of noncoding sequence that are more likely to harbor mutations that influence risk of disease.

## Introduction

Despite strong evidence that regulatory regions can be affected by pathogenic mutations, such as in fragile-X syndrome, β-thalassemia, Charcot-Marie-Tooth neuropathy, breast cancer and others [1–5], little has been done to quantify stretches of regulatory sequence in the context of both phylogenetic conservation and human-specific intolerance to variation, and then correlate it back to disease causing potential. While methods to assess phylogenetic conservation at a single site are established, such as GERP++ [6,7], purely phylogenetic approaches are at a risk of ignoring human specific regulatory sequence [8,9]. Furthermore, while efforts have been made to create predictors that seek to identify variants in noncoding sequence that might influence expression or have higher chance of causing disease [10–13], no framework has been introduced that focuses on standing variation in the human population to estimate the relative intolerance of a gene’s noncoding exome sequence to genetic variation. Since this regional-based approach proved effective for protein coding genes, it is natural to assess its application to noncoding exome sequence.

To assess whether noncoding sequence can predict genes that cause human disease through gene dosage aberration, we derive two measures: a phylogenetic conservation based score and a score reflecting intolerance to standing variation in a human population. To permit an unambiguous comparison to gene lists, we concentrate on each gene’s proximal regulatory regions: 5’ UTR, 3’ UTR, and the 250bp upstream of the transcription start site; recognizing that these three regions are only a subset of the relevant regulatory sequence for protein-coding genes. We generate a GERP++ region-based conservation score to assess the overall conservation of each gene’s proximal noncoding sequence [6,7]. To capture regulatory function that might be human-specific we formulate a novel human population genetic approach (ncRVIS). We then assess each gene’s proximal noncoding region for phylogenetic conservation and intolerance to genetic variation in the human population, and tie these scores back to genes known to cause disease due to a gene dosage aberration. An important clarification is that the current RVIS framework is a regional-based measure of intolerance to variation, and as such is complementary to traditional variant-level predictions. More recent ensemble-based predictors, such as CADD [12] and GWAVA [13] leverage multiple features including phylogenetic conservation to make predictions of functionality even for noncoding variants. To assess the levels of contribution from information beyond conservation, we adapted CADD and GWAVA into regionalized scores in a way analogous to ncGERP by taking the average CADD and subsequently the average GWAVA score across a gene’s noncoding proximal regulatory sequence as its ncCADD and ncGWAVA score, respectively (Methods).

Our results show that it is possible to use a combination of phylogenetic and human standing variation to identify regions of noncoding sequence that associate with gene-dosage sensitivity. Beyond the immediate noncoding flanking sequence of protein-coding genes, the framework introduced in this paper can be elaborated to include stretches of regulatory sequence beyond UTRs. Another important goal of this work is to illustrate that in addition to traditional phylogenetic signatures of important noncoding sequence, we can use signatures from human standing variation to help define boundaries of noncoding sequence that when considered as a unit might show an excess of mutations identified in cases compared to controls—similar to what is currently done in exome-sequencing studies where we assess excess mutations per each protein-coding gene.[14]

## Results

### Gene dosage lists

To evaluate whether a gene’s regulatory sequence can predict dosage sensitivity, we took four gene lists derived from independent sources. The first list contained OMIM disease-associated genes previously characterized as “haploinsufficient” [15]. The second list took a set of genes curated as dosage sensitive in ClinGen’s Genome Dosage Map (http://www.ncbi.nlm.nih.gov/projects/dbvar/clingen/). The third list—a novel list introduced here—relies solely on human polymorphism data from the 6503 whole exome sequences made available by the NHLBI Exome Sequencing Project (ESP) [16] to identify genes where, based on the sequence context and mutability, we observed fewer loss-of-function variants than we expected to observe. Finally, to look at the opposite end of the dosage sensitivity spectrum, we identified genes that are tolerant to copy number variations (CNVs) based on the CNV data from two large Database of Genomic Variants (DGV) studies [17–19].

### Deriving the noncoding genic scores

We used pre-calculated hg19 GERP++ scores (accessed January 2014) to calculate a single average GERP++ score across a gene’s noncoding sequence (3’ UTR, 5’ UTR and immediate promoter region). We refer to this score as the noncoding GERP (ncGERP) score. We then constructed a protein-coding conservation based score for each gene, pcGERP, by using the same methodology across the gene’s protein-coding sequence. As described in the relevant papers, GERP++ provides a score per nucleotide base, which has been shown to reflect a base’s conservation across the mammalian lineage [6,7].

**Fig 1:**
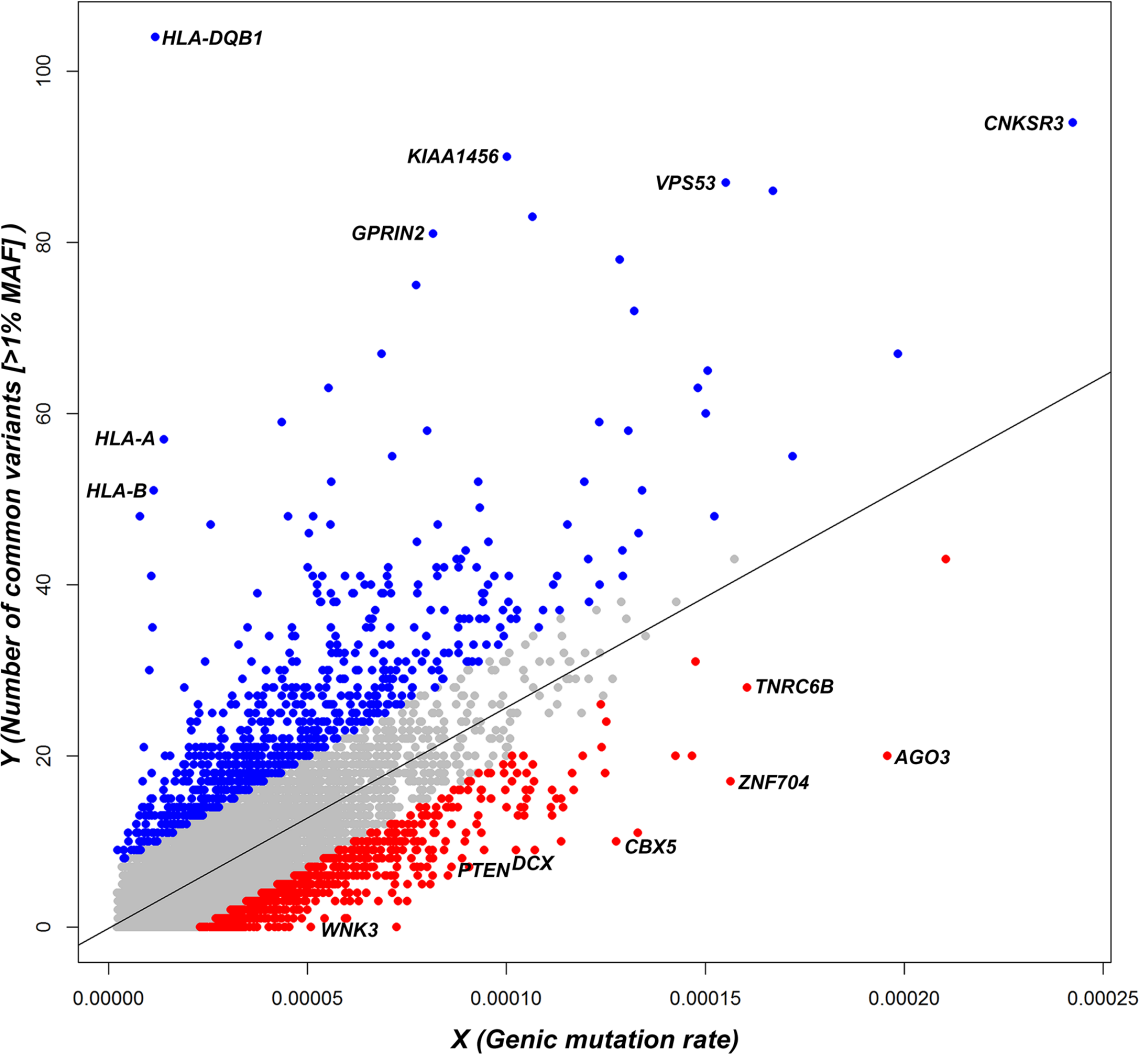
A regression plot that shows the regression of noncoding polymorphisms (Y) on an estimate of the noncoding sequence mutability (X) (S1 Data). Each dot represents the position of a gene in the regression plot and the corresponding regression line is provided. Annotations are made for the 5% extremes: red = 5% most intolerant, blue = 5% most tolerant.

A limitation of phylogenetic approaches is that they are unable to capture sequence with human specific function. To address this, we used the pattern of standing genetic variation in a human population. This approach is a noncoding formulation of the Residual Variance Intolerance Score (RVIS), a regression framework we recently developed to score the protein-coding sequence of genes in terms of their tolerance to functional genetic variation. We showed in Petrovski et al (2013) that this approach provides significant information for which genes are likely to carry protein-coding pathogenic mutations [15]. To adapt this approach to noncoding sequence, however, several changes are needed (Methods). First, instead of using the total number of observed variants to predict the expected number of common variants, we used the estimated mutation rate to reflect the mutability of the noncoding sequence. Second, since we cannot reliably distinguish functional and non-functional UTR variation we compared the prediction to all possible common noncoding variants. Finally, because most currently available exome kits do not provide sufficient coverage of UTRs, we relied on whole-genome sequence (WGS) data from 690 samples generated at the Duke Center for Human Genome Variation (CHGV (S1 Table). We first demonstrated that the RVIS itself when applied to protein-coding sequence of genes still has predictive utility when each of these adjustments are made, suggesting that a similar approach is possible for regulatory sequence (S2 and S3 Tables). For comparisons to our previously published protein-coding RVIS, we also generated RVIS-CHGV, a score that is the exact formulation of the published RVIS [15], but is dependent on the 690 CHGV whole-genome sequenced samples used to construct the ncRVIS score and similarly to ncRVIS, adopts the mutation rate of the effectively sequenced sites (Methods). We found that the noncoding ncRVIS and protein-coding RVIS-CHGV scores are weakly correlated (Pearson’s *r* ^*2*^ correlation of 0.04, S1A Fig). The ncRVIS (Fig 1), RVIS-CHGV and ncGERP scores and their corresponding genome-wide percentile scores can be found in S1 Data and at http://igm.cumc.columbia.edu/GenicIntolerance/.

### OMIM haploinsufficiency

To evaluate whether the ncGERP and ncRVIS scores correlate with known disease genes, we used the same gene lists as previously described [15]. We found that, using a logistic regression model, RVIS-CHGV, ncRVIS and ncGERP significantly predict OMIM haploinsufficient genes that have been linked through *de novo* mutations: p = 4.7x10^-21^ (AUC = 0.75), p = 2.4x10^-7^ (AUC = 0.63) and p = 2.7x10^-24^ (AUC = 0.78), respectively. A joint model of the three scores achieved an AUC of 0.816 when predicting OMIM haploinsufficient genes that have been linked through *de novo* mutations (Table 1). However, based on the other OMIM gene sets it does not appear that the noncoding sequence of genes can currently distinguish the broader set of OMIM disease genes, indicating that the patterns within the noncoding sequence are likely to be for the most part specific to diseases linked to haploinsufficiency (Table 1).

**Table 1:** Comparing protein-coding and noncoding genic intolerance scores.

*14*,*567 CCDS genes scored for*:	OMIM disease (1845 genes)	Recessive (721 genes)	HI^a^ (144 genes)	Dominant negative (306 genes)	HI^a^ and de novo (82 genes)	Essential Gene List (1919 genes)	ClinGen Dosage (181 genes)	Pearson’s *r* (*r* ^*2*^) to RVIS (EVS6503)
**protein-coding RVIS-CHGV**	***p* = 1.4x10** ^**-6**^ AUC = 0.543 [0.53–0.56]	***p* = 0.87** AUC = 0.512 [0.49–0.53]	***p* = 3.1x10** ^**-23**^ AUC = 0.712 [0.67–0.76]	***p* = 4.7x10** ^**-12**^ AUC = 0.631 [0.60–0.66]	***p* = 4.7x10** ^**-21**^ AUC = 0.753 [0.70–0.81]	***p* = 8.8x10** ^**-86**^ AUC = 0.665 [0.65–0.68]	***p* = 7.9x10** ^**-42**^ AUC = 0.748 [0.72–0.78]	**0.80 (0.63)**
**noncoding RVIS (ncRVIS)**	***p* = 0.61** AUC = 0.496 [0.48–0.51]	***p* = 0.13** AUC = 0.482 [0.46–0.50]	***p* = 9.5x10** ^**-6**^ AUC = 0.599 [0.55–0.65]	***p* = 0.08** AUC = 0.523 [0.49–0.56]	***p* = 2.4x10** ^**-7**^ AUC = 0.633 [0.57–0.69]	***p* = 4.7x10** ^**-15**^ AUC = 0.570 [0.56–0.58]	***p* = 1.3x10** ^**-19**^ AUC = 0.664 [0.62–0.71]	**0.17 (0.03)**
**noncoding GERP (ncGERP)**	***p* = 0.027** AUC = 0.516 [0.50–0.53]	***p* = 0.80** AUC = 0.497 [0.48–0.52]	***p* = 1.4x10** ^**-24**^ AUC = 0.720 [0.68–0.76]	***p* = 8.6x10** ^**-14**^ AUC = 0.627 [0.60–0.66]	***p = 2*.*7*x10** ^**-24**^ AUC = 0.780 [0.73–0.83]	***p* = 3.8x10** ^**-105**^ AUC = 0.659 [0.65–0.67]	***p* = 5.7x10** ^**-61**^ AUC = 0.778 [0.75–0.81]	**-0.25 (0.063)**
**Joint Model**	**AUC = 0.547**	**AUC = 0.515**	**AUC = 0.755**	**AUC = 0.652**	**AUC = 0.816**	**AUC = 0.695**	**AUC = 0.820**	**N/A**

To enable a matched comparison, the estimates in this table are based on a set of 14,567 CCDS genes with assessable scores across RVIS-CHGV, ncRVIS and ncGERP formulations. Both RVIS-CHGV and ncRVIS are based on the same population of 690 whole-genome sequenced samples from the CHGV.

^a^HI = Haploinsufficiency. To obtain the presented levels of significance, we used a logistic regression model to regress the presence or absence of a gene within the corresponding gene list on each of the genic scores.

Joint Model: The AUC of a combined logistic regression model that uses all three features. Correlation plots for the pairs of scores are available in S1 Fig.

### ClinGen dosage sensitivity map

**Fig 2:**
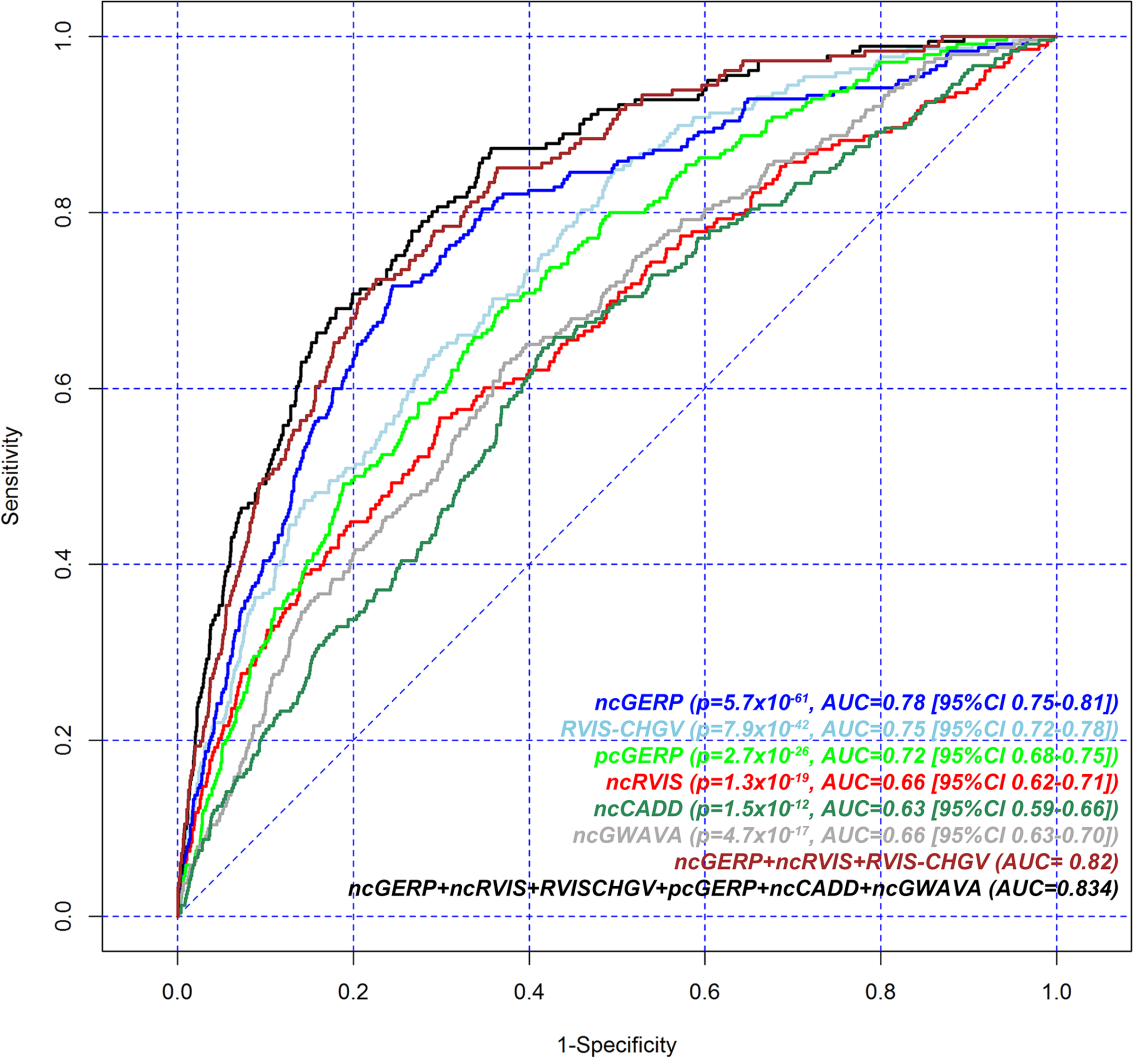
Receiver operating characteristic (ROC) curves to measure the ability of RVIS-CHGV, ncRVIS, pcGERP, ncGERP, ncCADD, ncGWAVA scores and two joint models to discriminate genes reported among ClinGen’s dosage sensitivity map from the rest of the human genome. Here, for a given score, all assessable genes were used. To obtain the presented levels of significance, we use a logistic regression model to regress the presence or absence of a gene among the ClinGen dosage sensitivity map list on each of the genic scores.

ClinGen’s dosage sensitivity map is another growing resource for genes that are curated by experts as being haploinsufficient or triplosensitive (http://www.ncbi.nlm.nih.gov/projects/dbvar/clingen/). As of the 1^st^ of May 2015, 263 genes had been annotated as having either “Some evidence for dosage pathogenicity” or “Sufficient evidence for dosage pathogenicity” (S2 Data). We repeated the gene dosage sensitivity assessment using this better curated set of 263 genes. We again observed that both protein and noncoding RVIS and GERP scores were significantly associated (*p* < 1x10^-16^) with genes curated to be dosage sensitive in ClinGen (Fig 2). ncGERP had the highest AUC (0.78) among the set of scores (Table 1 and Fig 2). Based on the curated ClinGen list this indicates that genes with highly conserved noncoding sequence relative to the rest of the genome are strongly correlated with gene dosage sensitivity.

Additional noncoding scores were constructed for both CADD [12] and GWAVA [13], by taking the average of the nucleotide-level score across a gene’s noncoding sequence (Methods). These are designated ncCADD and ncGWAVA, respectively. These scores were assessed against ClinGen’s dosage sensitivity genes as well; both ncCADD (p = 1.5x10^-12^; AUC = 0.63) and ncGWAVA (p = 4.7x10^-17^; AUC = 0.66) were also found to be significantly predictive of ClinGen’s dosage sensitivity genes (Fig 2).

We performed a joint logistic regression model to investigate performance in predicting ClinGen dosage sensitive genes from the genome-wide background using six features: the two RVIS and two GERP scores supplemented with two additional noncoding scores derived from ncCADD and ncGWAVA (S4 Table, Fig 2). There was modest improvement (AUC = 0.83) compared to a joint model using three features: ncGERP, ncRVIS and RVIS-CHGV (AUC = 0.82, Table 1).

### Loss-of-function deficient genes

The haploinsufficiency gene lists in the previous section relied on known Mendelian gene-disease relationships. Another way to identify a list of genes sensitive to gene dosage is to use the absence, where expected, of protein-coding loss-of-function (LoF) variants in a large human population (Methods). Such a population-based LoF deficient gene list highlights genes where changes in expression levels could be selected against, yet are independent of known gene-disease associations.

Using the standing variation from the ESP6500SI reference population we identified 1,235 LoF deficient genes (FDR < 1%) and 1,762 LoF control genes that had both an ncRVIS and ncGERP score assigned (Methods). We found that 2.3% of the 1,235 LoF deficient genes overlap with known OMIM “haploinsufficient” genes compared to one (0.06%) of the 1,762 control genes (Fisher’s Exact test, two-tailed p = 2.4x10^-10^). Given that the construction of the LoF deficient gene list is independent of gene-disease databases, this list could include genes where haploinsufficiency might be incompatible with life (non-viable), genes that are yet to be associated to disease through haploinsufficiency, or genes that cause disease through mechanisms other than haploinsufficiency. The overlaps of the 1,235 LoF deficient genes and the 1,762 control genes with the OMIM disease gene list (minus haploinsufficiency genes) were 26.6% and 13.2%, respectively (Fisher’s Exact two-tail p = 3.1x10^-20^).

**Fig 3:**
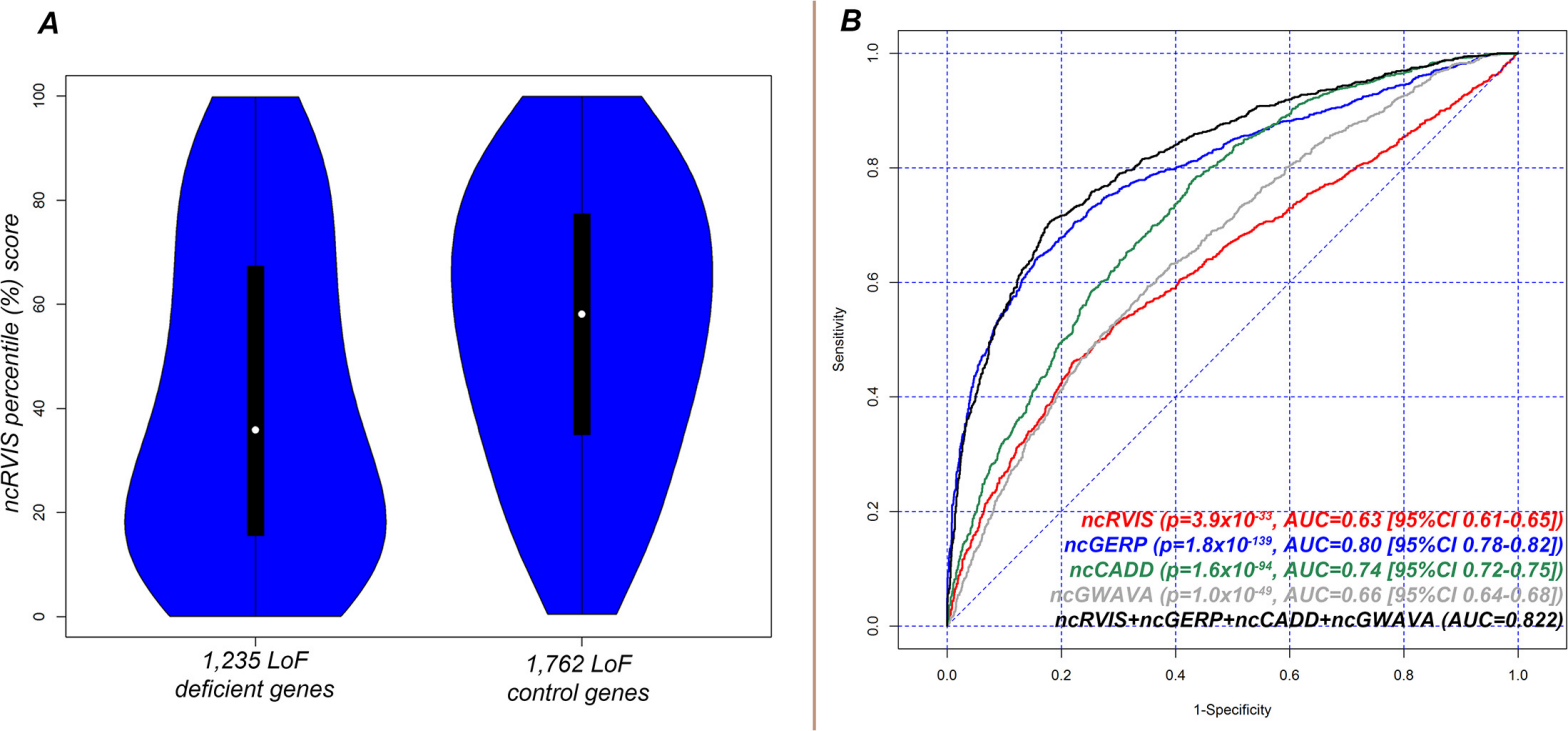
(A) Distribution of ncRVIS scores for the 1,235 loss-of-function deficient genes (left) compared to the 1,762 loss-of-function control genes (right). Median 37.95% vs. 58.09%; Mann-Whitney U test, p = 6.6x10^-34^. (B) Receiver operating characteristic (ROC) curves measuring the ability of RVIS, ncRVIS, pcGERP and ncGERP to discriminate between loss-of-function deficient and loss-of-function control genes. To obtain the presented levels of significance in **(B)**, we used a logistic regression model to regress loss-of-function deficient or control gene status for the combined 2,997 genes on each of the four genic scores.

We find that the median ncRVIS of the collective 2,997 genes is 50.8%. By comparing the distribution of ncRVIS scores between the 1,235 LoF deficient genes and the 1,762 LoF control genes we demonstrated that LoF deficient genes have significantly more intolerant noncoding sequence (median 37.9% vs. 58.1%; Mann-Whitney U test, p = 7.1x10^-34^, Fig 3A and S2A Fig).

We repeated the LoF deficient assessment with ncGERP, which showed that LoF deficient protein-coding genes preferentially have a more phylogenetically conserved regulatory sequence (median ncGERP 23.4% vs. 64.5%; Mann-Whitney U test, p = 3.4x10^-171^, S2B Fig. To understand whether information can be gained from combining ncRVIS and ncGERP scores, we used a multivariate logistic regression model, which showed that ncRVIS (p = 5.4x10^-6^) maintains a significant signal for predicting LoF deficient genes. This supports the expectation that regulatory functions specific to humans may not always be captured by ncGERP, while ncRVIS is likely picking up such patterns of human-specific selection within the regulatory sequence of genes where regulated dosage is critical to normal function. An investigation of alternative noncoding scores showed that both ncCADD and ncGWAVA were also significantly associated with LoF deficient genes (Fig 3B and S2 Fig).

Taken together, these data advocate prioritizing coding LoF mutations and potentially the regulatory region mutations among LoF deficient genes that have conserved or intolerant noncoding sequence (Fig 3B). This conclusion is corroborated by earlier results showing that ncRVIS and ncGERP are both significantly predictive of OMIM and ClinGen disease genes with a primary mechanism of haploinsufficiency.

### Copy Number Variant: Dosage insensitive genes

Individual CNVs distorting single or contiguous gene dosage have been linked to human diseases [20]. Inversely, genes that tolerate CNVs in the general population are unlikely to be dosage sensitive [21]. In this section we extended our assessment of ncRVIS and ncGERP to CNVs by asking whether a relationship exists between genes that have been shown to overlap (≥50% of the consensus coding sequence [CCDS]) with a deletion/duplication based on two study populations from Database of Genome Variation (DGV) [18]: Conrad et al (2010) and the 1K Genomes Project (2012) [17,19]. These two studies amass 1,602 individuals with comprehensive CNV data across 14,714 assessable CCDS genes. Of these assessable genes, 861 genes were found to have at least one CNV overlap among the combined population of 1,602 samples.

Genic tolerance to CNVs shows a clear relationship with the genes whose regulatory sequence also tolerates variation. We found that, on average, the 861 genes with a CNV overlap in these public databases have significantly higher ncRVIS (p = 2.3x10^-28^; AUC = 0.61) and ncGERP percentile scores (p = 9.2x10^-31^; AUC = 0.62) than the 13,853 genes without a reported CNV overlap in those data. Moreover, in a multivariate logistic regression model, RVIS (p = 3.1x10^-26^), ncRVIS (p = 1.9x10^-9^) and ncGERP (p = 8.9x10^-12^) each individually contribute to an improved overall prediction of genes that tolerate CNVs (AUC = 0.68).

The current data indicates that genes tolerating CNVs in the general population are also more likely to tolerate variation in their noncoding regulatory sequence. With 13,853 genes reporting no CNV overlap in this CNV dataset, much larger populations of high-quality, genome-wide CNV data are required to appropriately assess the question of whether intolerance in the regulatory sequence of a gene can strongly predict intolerance to specifically CNV deletions.

### Utilizing protein and noncoding RVIS

**Fig 4:**
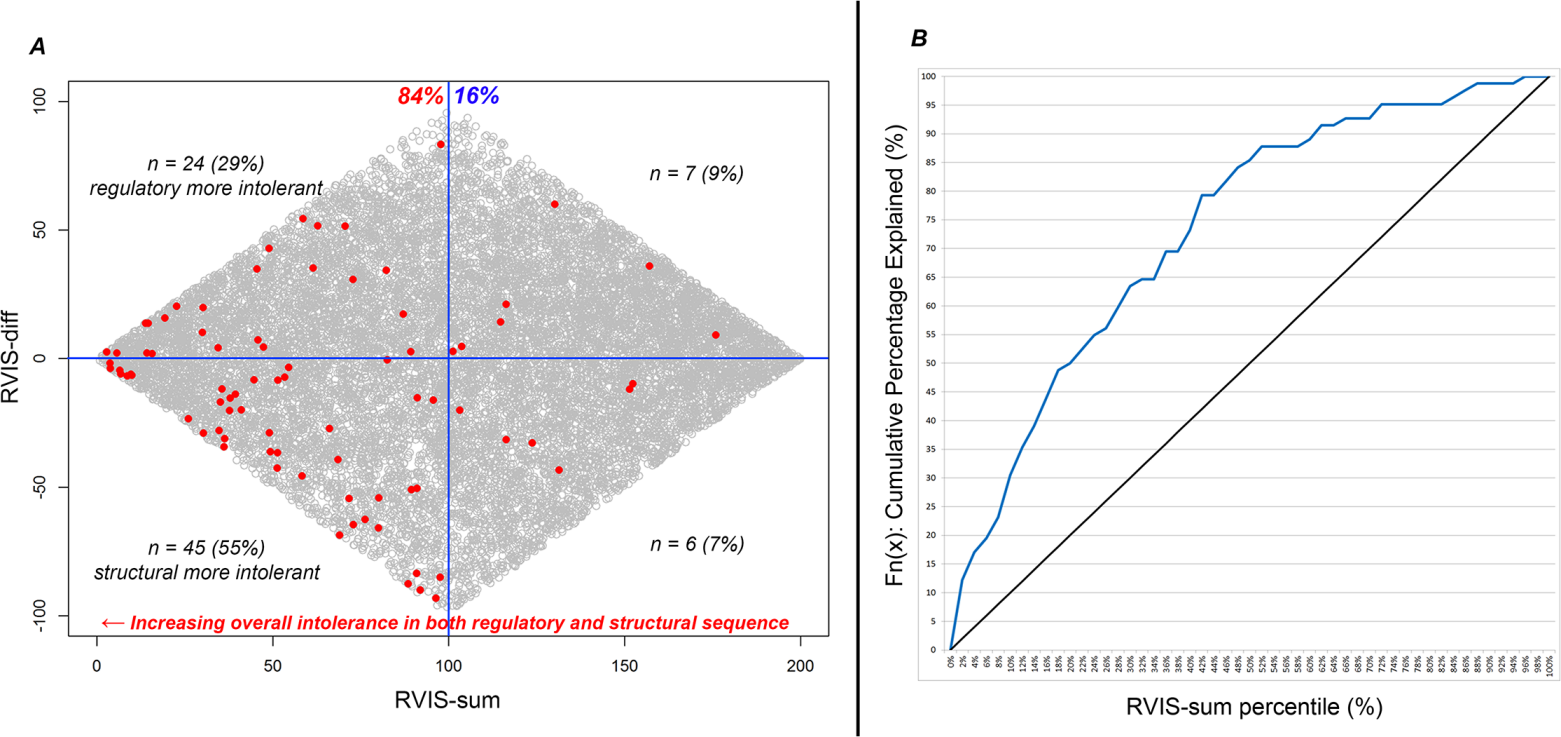
(A) Scatterplot of RVIS-sum (RVIS-CHGV + ncRVIS) and RVIS-diff (RVIS-CHGV–ncRVIS) scores. Each dot represents a gene. The grey dots represent the background genome-wide distribution. The red dots highlight the 82 OMIM haploinsufficiency genes with reported causal de novo mutations. A higher (positive Y-axis value) RVIS-diff score indicates genes where we might have a greater expectation of gene dosage aberrations being important compared with protein structure aberrations. A lower RVIS-sum (X-axis value) highlights genes that are increasingly intolerant in both their noncoding and protein-coding sequence. (B) A cumulative percentage plot for the RVIS-sum percentile accommodating the 82 OMIM halpoinsufficiency genes. At any given point on the X-axis (RVIS-sum percentile) we can determine what percentage of the 82 OMIM haploinsufficiency genes are accounted for.

The correlation between RVIS-CHGV and ncRVIS is *r* ^*2*^ = 0.04 (S1A Fig). We included RVIS-CHGV and ncRVIS in a multivariate logistic regression model and found that the signals from RVIS-CHGV and ncRVIS provided significant independent information in predicting OMIM haploinsufficiency genes annotated as carrying *de novo* pathogenic mutations. This multivariate logistic regression achieves an AUC estimate of 0.77; higher than each of the RVIS-CHGV (AUC = 0.75) and ncRVIS (AUC = 0.63) models. Next, we generated two additional scores for each gene (Fig 4A). The first was a combined genic intolerance assessment that considers the sum of the regulatory and protein-coding sequence by summing the values corresponding to a gene’s RVIS-CHGV and ncRVIS genome-wide percentiles, termed “RVIS-sum.” Using the list of OMIM haploinsufficient genes, we found that 84% of genes are in the lower 50^th^ percentile of RVIS-sum scores (Fig 4B). The second score is meant to reflect the extent to which these two measures diverge, which we term “RVIS-diff.” A positive RVIS-diff score indicates that the noncoding regulatory sequence of the gene is ranked as more intolerant than the protein-coding sequence of the same gene (Fig 4A).

### Interpreting neuropsychiatric loss-of-function *de novo* mutations

To assess how ncRVIS may be useful in interpreting mutations among patients, we specifically evaluated ncRVIS in the context of loss-of-function (LoF) *de novo* mutations reported across cohorts of individuals ascertained for the presence[22–33] and absence[30–34] of neuropsychiatric disorders. Here, loss-of-function *de novo* mutations were defined as nonsense, canonical splice and protein-coding indels that occurred within CCDS sequence and were absent in the ESP6500SI database. Firstly, among controls, we identified 180 LoF *de novo* mutations and the median ncRVIS percentile score of the genes those mutations were found in was 45.8%. When we considered the 494 LoF DNMs identified across cohorts of simplex trios ascertained for various neuropsychiatric disorders, we found that the LoF DNMs preferentially occurred among noncoding intolerant genes, with the median ncRVIS being 36.2%. No single LoF *de novo* mutation was observed twice among controls. Among cases, a *SCN1A* splice-donor *de novo* mutation was found among two probands, both ascertained for an epileptic encephalopathy [28]. Taking the combination of ncRVIS and protein-coding RVIS, the RVIS-sum vector, we found that among controls the median RVIS-sum was 85.91, while among neuro-ascertained cases it was 70.30 (Mann-Whitney U 2-tail test p = 0.001, S3A Fig). The significance remained even after excluding 19 loss-of-function *de novo* mutations among six previously known disease genes: *CDKL5*, *NRXN1*, *SCN1A*, *SCN2A*, *STXBP1 and SYNGAP1* (Mann-Whitney U 2-tail test p = 0.008).

A similar assessment is to use the information from a gene’s noncoding and protein-coding percentiles to calculate a single metric that reflects Euclidean distance from the most intolerant coordinate (0,0). Genes close to the (0,0) coordinate are characterized as having both the most intolerant noncoding and protein-coding sequence. We found that loss-of-function *de novo* mutations among cases preferentially occurred among genes closer to (0,0) with a median Euclidean distance for case-ascertained LoF DNMs of 0.588 compared to 0.698 for control LoF DNMs (Mann Whitney U test, *p* = 0.0035). A logistic regression model regressing case/control LoF DNM assignment on the Euclidean distance achieved an AUC of 0.58 (S3B Fig). We then combined the genic information from the Euclidean distance metric with the previously defined loss-of-function deficiency bioinformatics signature. We took only the LoF *de novo* mutations that fell in genes with a Euclidean distance ≤0.4 and also occurred in loss-of-function deficient genes with no more than a single LoF variant reported among the Exome Variant Server (EVS) [16] (S5 Table). This identified nine observations among the controls—corresponding to 5.0% of LoF DNMs—and 70 observations among cases, corresponding to 14.2% of all LoF *de novo* mutations among cases (Fisher’s Exact test two-tail p = 6.5x10^-4^; odds ratio of 3.2); a modest boost to what we got when we relied solely on the loss-of-function deficient bioinformatics signature (Fisher’s Exact two-tail p = 9.5x10^-4^; odds ratio of 2.4).

The list of genes carrying one of the 70 case loss-of-function *de novo* mutations includes established genes: *NRXN1*, *SCN1A* and *SCN2A*. The list also includes recently implicated genes: *CHD2 [35], CHD8 [36], KMT2E [37], MBD5 [35], SETD5 [38], and WDFY3 [39].* It is important to note that among the cases, the above six loss-of-function mediated pathogenic genes were of unknown significance when the *de novo* mutation data were first reported. This helps highlight the utility of this loss-of-function bioinformatics signature. The remaining case loss-of-function *de novo* mutations include some Mendelian disease genes with an existing neurological association, such as *NIPBL*, which is known to cause Cornelia de Lange syndrome [40] and *KMT2A*, which is known to cause Wiedemann-Steiner Syndrome [41]. The remaining genes with the same bioinformatics signature as the above established genes are: *ANK2*, *ARHGAP5*, *ASH1L*, *BRD4*, *BTAF1*, *DLL1*, *DNAJC6*, *DOT1L*, *EPHB2*, *FAM91A1*, *GIGYF2*, *INTS6*, *ITGA5*, *KIAA1429*, *LARP4B*, *MED13*, *MED13L*, *NCKAP1*, *NOTCH1*, *PHF3*, *POGZ*, *RALGAPA1*, *RALGAPB*, *RANBP2*, *RB1CC1*, *SPAG9*, *STAG1*, *UBN2*, *UBR5*, *ZC3H4* and *ZNF292* (S5 Table). It is unclear which of these genes could have their gene-disease association confirmed in the coming years; however, five of these candidates already have multiple LoF *de novo* mutation observations across neuropsychiatric ascertained patients: *ANK2*, *MED13L*, *NCKAP1*, *POGZ* and *ZNF292*.

## Discussion

Developing methods to recognize functional mutations in the regulatory part of the human genome is widely recognized as one of the central challenges facing modern human genetics. The difficulty is well illustrated by the results of the ENCODE project. Considerable effort and progress has been made in identifying parts of the genome with clear regulatory potential based on experimentally confirmed transcription factor binding sites and related approaches. However, since much of the genome is currently assigned a possible regulatory role it is difficult to use only those data to prioritize mutations in the study of human disease. Here, we show that population genetic and phylogenetic approaches can help fill this gap by adding further information about the possible functional role of a noncoding stretch of sequence. Integrating these approaches with the sequence regions identified by ENCODE [42] and related studies may ultimately prove to be the most effective approach. There are many additional regulatory sequences that can be included using the framework described here. Examples include distal enhancers, noncoding RNAs and larger promoter regions. However, correctly and unambiguously associating distal regulatory elements to the genes they regulate requires highly curated data, which is not yet straightforward to acquire. Therefore, here we focus only on regulatory sequences that can be unambiguously associated with specific genes in order to test the ability of the noncoding exome sequence to predict genes that cause human disease via gene dosage aberrations.

Using multiple resources, we show that dosage sensitive genes have distinct patterns of genetic variation in their proximal noncoding regulatory sequence. To the extent that more distant regulatory sequences may also carry variants that influence expression, we may expect a correlation between the intolerance patterns of a gene’s proximal and distal regulatory sequence. This possibility suggests that a sliding window of intolerance data throughout the human genome may provide a valuable new tool for identifying important regulatory sequence. Interpreting genome wide patterns of intolerance and relating those patterns to genes will not be a trivial task, but our results imply that genome wide patterns of intolerance have the potential to provide an important complement to other tools [42] used to identify important regulatory parts of the genome.

ncRVIS is a ‘regional-based’ guide to patterns of standing variation in the proximal noncoding sequence of a gene in the human population (Fig 1). It leverages the collective information from the standing variation in a stretch of noncoding sequence to assess whether that stretch of noncoding sequence has more or less polymorphic variation than expected. This is distinct from variant based scores that look at individual variants. By identifying stretches of noncoding sequence with preferential depletion of standing variation we are hypothesizing that in many cases this is driven by purifying selection among the human population acting against variation in that noncoding region as a whole, rather than at an individual variant site.

We and others have previously found that for the protein-coding sequence, RVIS and other estimates of human constraint are more indicative of disease causing genes than mammalian conservation [15,43]. However, in its current formulation, ncGERP outperforms ncRVIS in all current assessments. There can be a few explanations for this. Firstly, it is possible that the coding region is highly conserved throughout the genome to the point that there is limited allowance for big enough deviations between genic conservation in order to create an informative ranking. However, the noncoding regions may be more prone to allow such deviations. Secondly, the current ncRVIS formulation is based on a comparatively modest cohort (n = 690 samples).

There are two reasons we think ncRVIS remains important in light of the stronger signal observed from ncGERP. First, as we have shown throughout this work, the two scores are only weakly correlated (*r* ^*2*^ = 0.06, S1K Fig) and ncRVIS can add information beyond ncGERP. This is evidenced by the various dosage sensitive gene list assessments including the ClinGen assessment where in a joint logistic regression model of just ncRVIS and ncGERP, ncRVIS had a significant contribution (p = 7.2x10^-7^). This likely occurs, at least in part, for the interesting reason that there are genomic regions that have important functions only in humans. Evolutionary conservation will miss these regions, population genetic approaches will not. Second, the performance of ncGERP is close to its limit, as we already have a fairly good assessment of which sites are phylogenetically constrained, and which are not. ncRVIS, however, we anticipate will increase in predictive value as sample sizes of sequenced genomes grow, and thus a more extensive dataset of noncoding standing variation is available.

Alternative noncoding predictors of dosage sensitive genes, which take the overall propensity for a gene’s proximal noncoding sequence to score as more ‘functional’ based on the average nucleotide-level CADD or GWAVA scores, suggest that nucleotide-level predictors of noncoding functionality do appear to detect additional signatures of regulatory function beyond conservation. We observe correlation between a gene’s ncGERP and ncCADD score (*r* ^*2*^ = 0.32, S1V Fig), and to a lesser degree its ncGWAVA score (*r* ^*2*^ = 0.06, S1AA Fig), as a result of their dependence on conservation-based signals in their construction. In a joint model, however, we found that both ncCADD and ncGWAVA provide signal independent of ncGERP and ncRVIS when predicting human dosage sensitive genes (Fig 2). This suggests that even though conservation is a major component of their predictive signal for ClinGen’s dosage sensitive genes, additional information not directly captured by conservation might be captured by these two ensemble predictors (S4 Table).

Currently, the basic paradigm to analyze protein-coding sequence is to use aggregate statistics that integrate the effect of different rare mutations affecting the same functional unit, often defined as the protein-coding sequence of a single human gene. This has proven effective in whole-exome sequence data because we know the protein-coding sequence boundaries we need to consider in order to effectively aggregate variants that affect the same functional unit [14]. In order to effectively interpret whole-genome regulatory sequence data, and find the noncoding regions that harbor risk-influencing mutations, we need to learn to recognize the functional noncoding stretches of sequences that affect gene expression. Current annotations lack specificity to define truly functional noncoding regions. Here, we have shown that a phylogenetic and population genetic framework can help define and prioritize the functional noncoding regions, and this is expected to improve when combined with information about sequences with regulatory potential from ENCODE [42] and related resources. Here, we also explore additional signals beyond conservation and human standing variation by assessing the dosage sensitivity predictive value of ncCADD and ncGWAVA scores, two nucleotide-level scoring frameworks that in addition to capturing signals of conservation, leverage other features and annotations from the noncoding sequence. Such an integrated framework will enable the definition of intolerant noncoding regulatory regions that have been under both strong evolutionary (ncGERP) and human population (ncRVIS) constraint. For these reasons, ncRVIS and related approaches are likely to play a key role in the development of a statistical genetic framework to support the interpretation of large scale whole genome sequence data that will soon emerge, for example through the recently announced National Human Genome Research Institute (NHGRI) call for genomics of common disease centers. In this context, it is essential to appreciate that the resolution of the ncRVIS approach depends critically on the total number of individuals that have been sequenced, and therefore its value is expected to continue to increase as whole-genome sequenced sample sizes increase.

## Methods

### Data

Eleven data sources were used to develop and assess noncoding RVIS (ncRVIS) and noncoding GERP (ncGERP). As exome sequencing kits only capture a fraction of the untranslated region (UTR) sequence of genes, we utilized human whole-genome sequenced samples from the Institute for Genomic Medicine, Columbia University database (formerly Center for Human Genome Variation (CHGV), Duke University) to assess noncoding intolerance. We used Consensus Coding Sequence (CCDS) release 14 as the set of protein-coding genes of interest for scoring noncoding intolerance [44]. We used Ensembl 73 to define the UTR sequence of CCDS genes that did not overlap with CCDS protein coding regions of the same or overlapping genes [45]. We extracted gene-lists from OMIM database to reflect differing genetic models. We extracted a heavily curated list of haploinsufficient or triplosensitive genes from ClinGen’s Genome Dosage Map (http://www.ncbi.nlm.nih.gov/projects/dbvar/clingen/). For copy number variants (CNVs), we identified a set of deletions and duplications reported across two published studies: The 1K Genomes Project and Conrad et al. (2010) [17,19]. We used the GERP++ database to derive noncoding and protein-coding regional GERP scores to compare to phylogenetic conservation at the genic level [6,7]. We also used two noncoding ensemble nucleotide-level predictors, CADD [12] and GWAVA [13], to derive noncoding regional scores for each gene’s noncoding sequence as done for GERP++. Finally, we relied on the ESP6500SI [16] database to extrapolate a loss-of-function (LoF) deficient gene list, based on observing less than expected LoF variants in a gene.

### Defining the noncoding sequence of genes

To define the noncoding sequence for each gene, we relied on the Ensembl 73 noncoding annotation from that gene’s canonical transcript (downloaded 19^th^ September 2013). We refer to noncoding exonic sequence of genes as the collection of 5'-UTR, 3'-UTR and an additional non-exonic 250bp upstream of transcription start site (TSS). The 5’ and 3’ UTR are derived based on the canonical transcript annotation. The additional 250bp upstream of the TSS is defined as the 250 bases upstream of the initial exon junction, taking into consideration whether the transcript lies on the (+/-) strand. For three Ensembl genes (*PKD1L2*, *SPIB*, and *UGT2A1*) that had multiple canonical transcripts, we took the larger of the two canonical transcripts.

Given the challenge of defining the upstream promoter region, we opted to choose a relatively small number of bases immediately adjacent to the TSS, and this was set at 250 for all genes. Defining different sized promoters per gene guided by the distribution of conservation (e.g., GERP++ scores) or human polymorphism density would create a situation where we specifically define the promoter region of the gene we are assessing based on the more intolerant or more conserved set of bases upstream of the TSS. Evidently, this could create a bias towards intolerant or conserved promoters in our score, and therefore we prefer for this formulation to define the upstream promoter regions agnostic to the data we use for the assessments.

The initial dataset was comprised of 56,715 noncoding units. These 56,715 units resulted in 19,563 unique Ensembl genes. We found that 18,507 genes had a 5’ UTR (average = 260 bases, median = 182 bases). 18,638 genes had a 3’ UTR. And, by design, all 19,563 genes had a 250bp promoter region. Of the 19,563 genes, 18,148 (92.77%) had all three noncoding units represented. 849 had only two units represented, whereas 566 were based solely on their promoter unit, with no UTR boundaries defined.

The overall genomic noncoding sequence comprised of these 19,563 unique Ensembl genes is 34,065,650 bases. These reflect the number of sites prior to exclusion of inadequately covered sites and sites that overlap with protein-coding position among other genes, as discussed below.

### Whole-genome sequenced samples

We found that whole-exome sequencing is inadequate for capturing the noncoding exonic sequence of protein-coding genes. To derive a noncoding RVIS, and to generate a comparative protein-coding RVIS based on the same subset of samples, we selected 690 internally sequenced (CHGV) control-approved whole genomes (78% Caucasian ancestry). For these 690 whole-genome sequenced samples, an average of 92.7% sites were covered, with at least 10-fold coverage across the 34,065,650 Ensembl defined noncoding sites. Similarly, relying on CCDS release 14 for the protein-coding sequence, we observed that these 690 whole-genome sequenced samples covered on average 94.6% of the 33,266,994 protein-coding sites in CCDS release 14 with at least 10-fold coverage.

The set of phenotypes contributing to the whole-genome sequenced set of 690 cases is summarized in S1 Table.

### Pruning the noncoding sites for inadequate coverage and protein-coding overlap

It is our experience that sites sequenced with consistently good coverage represent sites with more reliable alignment and variant calling than sites where coverage is sparse and inconsistently represented among a population. S4A Fig (blue curve) represents the number of our 690 whole-genome sequenced samples that had at least 10-fold coverage (Y-axis) versus the cumulative percentage of the 34.1Mbp Ensembl-defined UTR noncoding sites (X-axis). The intersection between the blue curve and green line (an illustrative cutoff) indicates that at this point approximately 92% of samples have at least 10-fold read coverage at approximately 83% of the Ensembl noncoding sites. Together, the green threshold line and the blue sample-site coverage profile partition the space into four regions. Region II and region III represent the overall heterogeneity of coverage and the amount of noncoding sequence pruned from analysis, respectively. Shifting the green line left retains noncoding sequence (smaller region III) at a cost of increased coverage heterogeneity (larger region II). Moving the threshold right reduces the noncoding sequence used in the analysis, but also reduces the noise from coverage heterogeneity. There are multiple ways to select a cutoff from these data; however, a balanced approach is to choose a cutoff that ensures region II and region III are as close as possible in terms of area.

To evaluate the area for region II and region III, we first smooth the sample-coverage profile (blue curve) by fitting smooth spines, as illustrated by S4B Fig where the blue dots represent the original profile, while the red curve represents the smoothed splines. The smoothed curve traces the original data well. We use the smoothed curve to compute the areas for region II and region III (through numeric integrations) for a selection of evenly spaced cutoff values. The areas for region II and region III for different cutoff values is shown in S4C Fig, with red and blue curves representing region II and region III, respectively. We choose the balanced cutoff to be the point where the curves intersect, representing a balance between loss of data (noncoding sequence sites) and variability from coverage heterogeneity. The method yields an optimal value of 0.074 based on the noncoding sequence data. This suggests that removing the 7.4% most inconsistently covered noncoding sites corresponds to requiring noncoding sites to have at least 67% of samples with at least 10-fold read coverage. We selected 70% for the manuscript, corresponding to removing the 8% ‘noisiest’ noncoding sites with respect to inadequate coverage at the population level (S4D Fig). We performed a sensitivity test varying the 70% threshold to a threshold of 60% (*r* ^*2*^ = 0.986) or 80% (*r* ^*2*^ = 0.971) and show that the final ncRVIS score is not highly sensitive to varying this threshold (S5 Fig).

Requiring ≥70% of the 690 samples have at least 10-fold coverage at a site prunes the noncoding sequence down to 31,355,520 (92.0%) of the initial 34,065,650 Ensembl-defined UTR noncoding sites. For the CCDS release 14 protein-coding sites, we found that this pruning process retained 31,528,600 (94.8%) of 33,266,994 CCDS sites.

Although it is expected that some variants in protein-coding sequence will affect gene regulation and that these would be easily associated with the genes they fall in, we excluded all protein-coding regions in order not to confound the ncRVIS score with protein-coding sequence. Through this additional step, we ultimately retained 31,112,586 (91.3%) of the 34,065,650 noncoding sites. We find that on average each of the 690 genomes has at least 10-fold coverage across 97.8% of the 31.1Mbps of noncoding sequence used to derive ncRVIS. Overall, the GC content of the 5’ UTR sequence is 61% in comparison to the GC content of the 3’ UTR sequence which is 42.5%.

Combining the three noncoding components into a single genic noncoding unit resulted in 19,484 (99.6%) of the 19,563 Ensembl genes retaining at least one noncoding component. The average length of noncoding sequence across the 19,484 Ensembl 73 genes was 1,597 (median = 1,096 sites).

Finally, we defined ncRVIS “assessable” genes as Ensembl genes not located on the Y chromosome, and with at least 70% of their noncoding sequence surviving the aforementioned filters. Through this, we retained 16,273 CCDS release 14 protein-coding genes that fulfilled the coverage requirements of having at least 10-fold coverage of at least 70% of the gene protein-coding sites across at least 70% of the CHGV whole-genome sequenced samples. The overlap between CHGV-derived RVIS and ncRVIS indicates that 15,471 genes were “assessable” for both coding and noncoding RVIS (S1 Data).

### Calculating the genic coding and noncoding sequence mutation rate (X)

To accommodate the uncertainty surrounding the percentage of noncoding sequence sites that are neutral, we used an alternative metric to reflect mutability of a given sequence context in our ncRVIS and RVIS-CHGV adaptations. For the sites reflecting a genic unit (noncoding or coding) we use an in-house script developed by Dr Yujun Han. This script leverages the DNA tri-mer mutation rate matrix (kindly provided by Drs. Shamil Sunyaev and Paz Polak of The Broad Institute of MIT and Harvard, Cambridge) to generate a mutation rate for a given genic unit, which is calculated for each gene by summing the point mutation rates across the effectively captured sequence [28].

The mutation rate model provides an estimated rate of mutation per base. The rate is based solely on three bases: the interrogated base, the base immediately before, and the base immediately after the interrogated base. The model is based on human, chimpanzee and baboon genomic sequences [46]. The mutation rate model does not currently account for effects of larger sequence context or biological processes that affect mutation rate, such as background selection, distance to CpG islands, or replication timing. At the level of the gene, like others [43], we find very high correlation (*r* ^*2*^ = 0.95) between gene coding length and mutation rate. While the high correlation suggests it is possible to use gene size as a proxy, we prefer leveraging the mutation rate to accommodate for some additional information that is likely lost when using gene size. The source code can be found in S3 Data.

### Calculating the number of common variants in the noncoding sequence

All sequencing was performed on the Illumina HiSeq2000 platform (Illumina, San Diego, CA) in the Genomic Analysis Facility in the Center for Human Genome Variation (CHGV) at Duke University. After sequencing, reads were aligned to Genome Reference Consortium Human Genome build 37 (GRCh37) using the Burrows-Wheeler Alignment Tool (BWA)[47] and PCR duplicates were removed using Picard software (http://picard.sourceforge.net). The reference sequence we used is identical to the 1000 Genomes Phase II reference and it consists of chromosomes 1–22, X, Y, MT, unplaced and unlocalized contigs, the human herpesvirus 4 type 1 (NC_007605), and decoy sequences (hs37d5) derived from HuRef, Human Bac and Fosmid clones and NA12878. Variants were called using the Genome Analysis Toolkit[48]. SnpEff was used to annotate the variants[49].

To construct the ncRVIS score, we defined the minor allele frequency threshold dividing “common” and “rare” variants as ρ. To identify the number of variants with a MAF > ρ in the noncoding region of a gene, we use an in-house package, Analysis Tool for Annotated Variants (ATAV). ATAV communicates with our in-house relational database that houses all the variant call (and non-carrier) information for all sites across each of the 690 whole-genome sequenced samples. Additional filtering consisted of excluding indel calls and requiring a minimum of 10-fold coverage to call a variant (or be confident that a variant wasn’t present in a non-carrier sample). To increase confidence in called variants the following additional filters were applied: relying on GATK VQSLOD “pass” and “intermediate tranches,” requiring a QUAL score of at least 30, a QD (quality by depth) score of at least 2, a genotyping quality (GQ) score of at least 20, and a fisher strand bias (FS) score of less than 60. For noncoding regions, we considered all common variants residing in the noncoding sequence as contributors to (Y), the number of common variants.

For the CHGV-based protein-coding RVIS score (based upon the same 690 whole-genome sequenced samples as ncRVIS), we adopted the same criteria as in our earlier work introducing RVIS. That is, synonymous protein-coding variants did not contribute to the number of common ‘functional’ variants when deriving the CHGV protein-coding RVIS score. However, we did assess a secondary score (RVIS-Yall) for comparison purposes. RVIS-Yall considered all protein-coding variants as eligible to contribute to (Y), including the putatively neutral, synonymous coding variants.

### Deriving the noncoding RVIS (ncRVIS) score

We defined Y as the total number of common (Minor Allele Frequency [MAF]>ρ) SNVs in the noncoding sequence of a gene, and X as the effective mutation rate of the noncoding sequence of the gene, using the mutation matrix described previously. We then regressed Y on X and took the studentized residual as the noncoding Residual Variation Intolerance Score (ncRVIS). The raw residual was divided by an estimate of its standard deviation to account for differences in variability that come with differing mutational burdens. The ncRVIS then provides a measure of the departure from the (genome-wide) average number of common variants found in the noncoding sequence of genes with a similar amount of noncoding mutational burden. When S = 0, the gene has the average number of common noncoding variants given its total mutational burden; when S<0, the gene has fewer common noncoding variation than predicted; when S>0, it has more. Each ncRVIS is then translated to a corresponding percentile to reflect the relative position of that gene on the genome-wide spectrum of ncRVIS based on the relative intolerance of that gene’s noncoding sequence. S1 Data contains the X and Y estimates used to construct ncRVIS. The R code to reproduce ncRVIS relies on the MASS package [50]: *studres(glm(Y~X))*.

As we only had 690 whole-genome sequenced samples available, we chose to adopt a MAF threshold ρ of 1% for the noncoding RVIS and RVIS-CHGV. We had in our previous publication explored the behavior of the original RVIS for ρ of 0.01% and 1%, and found both of these to be strongly correlated with ρ = 0.1% (Pearson's *r* = 0.849 and Pearson's *r* = 0.813, respectively).

The collection of genomes used to derive ncRVIS includes various sample ascertainments (S1 Table). Given that we use the mutation rate matrix to define the underlying mutation rate (X), and only consider variants with a MAF>1% when determining (Y), we consider it highly unlikely that case-ascertained variants could be systematically influencing the current ncRVIS or RVIS-CHGV scores. We highlight *F8* as the single gene that might require careful interpretation due to our collection of WGS samples that were ascertained for haemophilia.

Under the residual variation intolerance framework, ncRVIS will not correlate with either the noncoding mutability or noncoding sequence length. To confirm this, we find that the correlation between ncRVIS and the corresponding mutability or size of the effective noncoding sequence to be *r* ^*2*^ = 3.0x10^-8^ and *r* ^*2*^ = 2.0x10^-5^, respectively (S1B and S1C Fig). We further confirmed that the ncRVIS is not strongly correlated to the corresponding genes ‘protein-coding’ sequence size or ‘protein-coding’ mutability: *r* ^*2*^ = 0.0031 and *r* ^*2*^ = 0.0026, respectively (S1D and S1E Fig). We do note, however, that there is high correlation between a gene’s noncoding sequence length (number of bases) and its derived mutability using the mutation rate matrix (*r* ^*2*^ = 0.9493, S1F Fig).

### Formulations of the protein-coding RVIS

We first assessed the likely impact of using the estimated mutation rate instead of the observed variation by comparing two formulations of the original RVIS. To construct RVIS-mut we replaced the observed variation among the EVS population with the estimated mutation rate for that gene to represent (X) and kept the original Y variable from RVIS. Reassuringly, we find that RVIS-mut, using the estimated mutation rate, correlates highly (Pearson’s *r* ^*2*^ = 0.83) with that using the total number of variants in each gene (RVIS) (S2 Table and S1G Fig).

We next evaluated the effect of not being able to identify functional mutations by comparing RVIS to a third formulation (RVIS-YALL). For RVIS-YALL we again use the effective mutation rate to represent X; however, we now permit all common protein-coding variants (including synonymous variants) for the Y variable. We find that RVIS-YALL remains highly correlated with the original RVIS (Pearson’s *r* ^*2*^ = 0.59, S1H Fig); more importantly, it remains predictive of genes causing Mendelian disease (S2 Table).

Finally, we show that a fourth formulation of RVIS, using an independent set of 690 whole-genome samples that were sequenced at the CHGV (RVIS-CHGV), remains highly correlated with the original RVIS (Pearson’s *r* ^*2*^ of 0.63, S1I Fig) despite a decreased sample size, and continues to be significantly predictive of the disease gene lists, with the exception of genes causing recessive disease (S2 Table).

These comparisons suggest that, in principle, the ncRVIS formulation should work similarly to RVIS when regulatory sequences are subject to purifying selection.

### Comparison to estimates of phylogenetic conservation

In our original RVIS paper we used omega (ω) as the phylogenetic approach to compare non-synonymous substitutions per non-synonymous site (dN) to the synonymous substitutions per synonymous site (dS) (aka Ka/Ks, dN/dS). Given we are now interested in noncoding sequence, we have generated an alternative estimate to assess whether correlation exists between the ncRVIS and that of possible phylogenetic conservation at noncoding sites. For each gene we constructed two conservation vectors: one reflecting the noncoding sequence of a gene after excluding protein-coding overlapping sites (ncGERP), and the other reflecting the protein-coding sequence of a gene (pcGERP). Both conservation vectors were based on the average GERP++ score [6] of the qualifying chromosomal sites within the defined sequence.

We found that ncGERP and pcGERP were moderately correlated to each other (*r* ^*2*^ = 0.30). Compared to ncRVIS, both ncGERP and pcGERP had low correlation: *r* ^*2*^ = 0.06 and *r* ^*2*^ = 0.04, respectively. Likewise, compared to previously described RVIS [15], both ncGERP and pcGERP had relatively low correlation *r* ^*2*^ = 0.06 and *r* ^*2*^ = 0.15, respectively. These five correlation tests were performed based on the 14,998 genes with the corresponding scatterplots available in S1J–S1N Fig.

**Fig 5:**
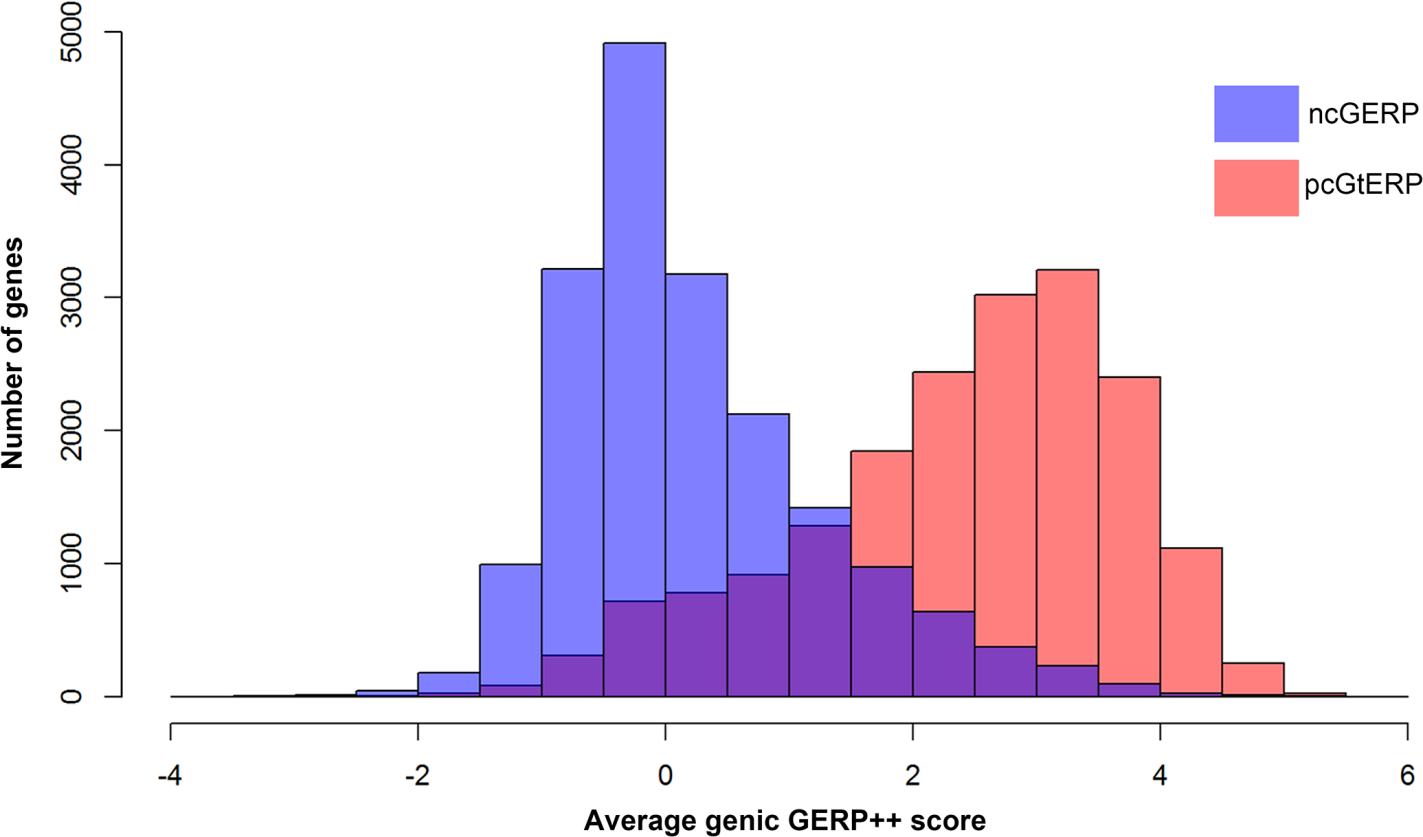
Overlaid histograms of ncGERP (blue) and pcGERP (red). These data show that the two form very different genome-wide distributions (medians: ncGERP -0.02 versus pcGERP 2.64). Moreover, pcGERP tends to present with a slightly platykurtic, left-skewed distribution (γ_2_ = -0.10, γ_1_ = -0.66) compared to ncGERP, which reflects a more leptokurtic, right-skewed distribution (γ_2_ = 0.97, γ_1_ = 0.96).

Interestingly, we found that pcGERP was inferior to ncGERP when comparing the 1,235 LoF deficient genes to the 1,762 LoF control genes, as described above (median pcGERP 32.38% vs. 65.57%; Mann-Whitney U test, p = 5.6x10^-141^; in comparison to median ncGERP 23.39% vs. 64.49%; Mann-Whitney U test, p = 3.4x10^-171^). While protein-coding genes are generally fairly phylogenetically conserved overall, there is variability inside the protein-coding genes in the phylogenetic conservation that correlates with whether a site causes disease or not. Overall, however, the majority of protein-coding genes are reasonably conserved across species. This leaves less scope for pcGERP variability among genes that can then be related to disease gene status (Fig 5). This is less true for the regulatory regions, where single-site variation is unlikely to systematically experience the same overall constraint as sites coding for structural components of the proteins. As a consequence of this, there is more scope for variability among the average ncGERP across the genome-wide spectrum of genes (Fig 5).

### Alternative noncoding nucleotide-level scores

Literature includes alternatives to GERP++ for quantifying the degree of importance (sometimes referred to as functionality) of noncoding sequence in the human genome. Unlike GERP++, which is a direct measure of the phylogenetic conservation of a single site or a stretch of sequence, more recent alternatives include ensemble based predictors that leverage many features beyond conservation.

Although we recognize that nucleotide-level scores were constructed specifically for variant-level assessments; we nonetheless investigate whether a regionalized version of these scores could add information to predicting dosage-sensitive gene lists as well or better than ncRVIS or ncGERP. To this end, we calculated noncoding regional scores based on two popular nucleotide-level scoring frameworks: CADD [12] and GWAVA [13]. Using the same coordinates as ncGERP, we took the average CADD and GWAVA scores across the defined noncoding regions as a gene’s noncoding score.

To calculate regional noncoding CADD scores, referred to as ncCADD, we used the scaled C-scores from CADD version 1.0 [12]. In a regionalized form, ncCADD reflects the average CADD score for all possible single nucleotide substitutions across a gene’s defined noncoding sequence.

For regionalized noncoding GWAVA, referred to here as ncGWAVA, we downloaded the required training data and scripts from (ftp://ftp.sanger.ac.uk/pub/resources/software/gwava/) and followed instructions given by the developers to generate the site-specific scores for all noncoding exome sites. We were advised that for UTR sequence the TSS-distance matched training set would be optimal (personal communication with Dr. Graham Ritchie). Using the TSS-distance matched training set we derived the GWAVA score for each noncoding nucleotide site in a gene’s defined noncoding exome sequence and took the average to be the gene’s ncGWAVA score.

Neither CADD nor GWAVA were specifically developed to be interpreted as regional assessments. However, understanding the overall importance of a gene’s noncoding sequence as inferred from CADD or GWAVA could still be of interest. Both noncoding scores are provided in S1 Data. Scatterplots assessing correlations with other scores (including ncGERP) are available in S1S–S1AA Fig.

### Comparing ncRVIS across the noncoding sub-units

To assess the possible contributions of each ncRVIS subunit, we generated an ncRVIS score for promoter regions, 5’ UTR regions, and 3’ UTR regions for the set of 10,726 genes that had “assessable” sequence across all three distinct noncoding subunits. To permit comparisons with the original RVIS score, we further restricted comparison to the 9,644 distinct genes that also had a published RVIS score (Petrovski et al. 2013 [15]), an assessable ncRVIS score. We find that the highest correlation with the ncRVIS score comes from the 3’ UTR ncRVIS (*r* ^*2*^ = 0.79), compared to promoter and 5’ UTR regions, which had *r* ^*2*^ correlation of 0.25 and 0.20, respectively (S3 Table).

### Using standing variation to identify loss-of-function deficient genes

To generate a loss-of-function (LoF) deficient gene list, we take the five distinct mutation rates provided per gene by Samocha et al. (2014)[43] and calculate the expected frequency of protein-coding loss-of-function variants for each assessable consensus coding sequence (CCDS) gene (*Ps*). We achieve this by first summing the mutation rates corresponding to the three loss-of-function variant effect classes (nonsense, splice and frameshift) and dividing that by the total sum of the mutation rates of every possible mutation effect in the gene. We then use the resulting rate to determine the percentage of variants in a gene that we expect to result in a LoF effect, accommodating for the mutation rate. Based on the above, the average percentage of possible protein-coding mutations in a gene that are expected to result in a loss-of-function annotated variant (whether it is subsequently selected against or not) is ~9.22% of the sum of all possible protein-coding and canonical splice site mutation events. We then use the ESP6500SI database (accessed 20^th^ March 2013) to extract for each gene both the total number of observed unique variants (*n*) and specifically the number of observed unique loss-of-function variants reported in the CCDS of each gene (*x*). This gives us our observed rate of LoF variants given all the protein-coding variation identified in the gene. Taking a gene’s expected percentage of unique loss-of-function variation under neutrality as calculated by (*Ps*), a subset of 1,235 genes with ncRVIS and ncGERP scores were identified as being significantly deficient of loss-of-function variants using a one-sided binomial exact test with Benjamini & Hochberg false discovery rate multiple-testing correction (FDR = 1%)[51] (S4 Data). As a comparative group, we identified a set of 1,762 ‘control’ genes where we observe greater than the expected number of loss-of-function variants. While this list of LoF control genes cannot be interpreted as significantly LoF tolerant, we consider the list a useful comparative group to the genes found to be significantly LoF deficient. It is clear that we are currently missing some true LoF intolerant genes due to insufficient resolution (power) from the EVS reference cohort. The result of this reduced power is that the majority of the exome is considered non-informative for LoF deficiency. Larger cohorts will enable better discrimination of truly LoF deficient genes. However, even though it is currently an incomplete list, the list of genes that are already significantly LoF deficient is already a valuable resource.

Finally, to illustrate that this list is robust to false positives driven by how much of the gene has been effectively sequenced, we repeated the exact implementation only this time asking whether any genes were significantly deficient of synonymous (presumed neutral) variation. In comparison to the LoF assessment where we identified 1,235 genes with an FDR < 1%, genome-wide the lowest FDR among the synonymous assessment was an FDR of 61%, with no other gene achieving an FDR < 99.99% for the synonymous assessment. This further highlights the integrity of this approach to detect LoF deficient genes in the human genome.

## Supporting Information

S1 Fig Scatter plots. Scatter plots between different metrics and features used in this manuscript. S1A: ncRVIS and RVIS-CHGV; S1B: ncRVIS and noncoding mutation rate; S1C: ncRVIS and noncoding sequence size; S1D: ncRVIS and coding sequence size; S1E: ncRVIS and coding mutation rate; S1F: noncoding sequence size and noncoding mutation rate; S1G: RVIS and RVIS-mut; S1H: RVIS and RVIS-YALL; S1I: RVIS and RVIS-CHGV; S1J: ncGERP and pcGERP; S1K: ncRVIS and ncGERP; S1L: ncRVIS and pcGERP; S1M: RVIS and ncGERP; S1N: RVIS and pcGERP; S1O: ncRVIS and 3’ UTR ncRVIS; S1P: ncRVIS and promoter ncRVIS; S1Q: ncRVIS and 5’ UTR ncRVIS; S1R: RVIS and ncRVIS; S1S: ncCADD and noncoding sequence size; S1T: ncCADD and ncRVIS; S1U: ncCADD and RVIS-CHGV; S1V: ncCADD and ncGERP; S1W: ncCADD and ncGWAVA; S1X: ncGWAVA and noncoding sequence size; S1Y: ncGWAVA and ncRVIS; S1Z: ncGAVA and RVIS-CHGV; S1AA: ncGWAVA and ncGERP.
(DOCX)

S2 Fig Distribution of genic percentile scores for LoF deficient and control genes. The distribution of genic score percentiles for the 1,235 LoF deficient (red distribution) compared to the 1,762 LoF control (blue distribution) genes. (A) ncRVIS percentiles, (B) ncGERP percentiles, (C) ncCADD percentiles and (D) ncGWAVA percentiles.
(TIF)

S3 Fig De novo mutations among case and control trios. **(A)** Distribution of RVIS-sum scores for genes affected by loss-of-function de novo mutations. A median RVIS-sum score of 70.3 observed among 494 case-ascertained de novo mutations and a median of 85.9 among 180 de novo mutations from controls not ascertained for a neuropsychiatric disorder (Mann-Whitney U test p = 1.5x10^-3^). **(B)** Receiver operating characteristic (ROC) curve measuring the ability of the Euclidean distance for each LoF de novo mutation to discriminate between case and control ascertained LoF DNMs (AUC = 0.58 [95% CI 0.53–0.63]).
(TIF)

S4 Fig Threshold selection method. In S4A, S4B, S4D Figures the blue curve represents the number of our 690 whole-genome sequenced samples that had at least 10-fold coverage (Y-axis) versus the cumulative percentage of the 34.1Mbp Ensembl-defined UTR noncoding sites (X-axis). For example, in S4A Fig the intersection between the blue curve and green line (an illustrative cutoff) indicates that at this point approximately 92% of samples have at least 10-fold read coverage at approximately 83% of the Ensembl noncoding sites, and less than 10-fold coverage at approximately 17% of the Ensembl noncoding sites. S4C Fig represents the area of region II (red) and region III (blue in S4A Fig) for different X-axis cutoffs. The optimal threshold we decide to use as an x-axis cut-off in S4A Fig is selected by finding the intersection between the blue and red curves in S4C Fig–as represented by the red line in S4D Fig.
(TIF)

S5 Fig Threshold selection scatter plots. Scatter plots between three different ncRVIS sets, each generated with a different threshold for the percentage of samples with 10-fold coverage required at a given site for that site to be included. (A) compares the correlation between the choice of a 70% or 60% cut-off (r^2^ = 0.99); (B) compares the correlation between the choice of a 80% or 60% cut-off (r^2^ = 0.95); and (C) compares the correlation between the choice of a 80% or 70% cut-off (r^2^ = 0.97). These plots show that ncRVIS is not highly sensitive to varying this threshold.
(TIFF)

S1 Table Cohort summary. Cohort of whole-genome sequenced samples used to construct the ncRVIS and RVIS-CHGV scores.
(DOCX)

S2 Table Comparisons of deviations from original RVIS. Based on 16,275 consensus coding sequence (CCDS) genes with assessable scores across all four RVIS formulations. Reflecting 96% of the CCDS release 9 genes scored in Petrovski et al (2013). Details on adopted gene-lists can be found in Petrovski et al (2013). **RVIS:** Based on the ESP6500 dataset. X = observed protein-coding variants, Y = common (>0.1% minor allele frequency) functional variants [15]. **RVIS-mut:** Based on the ESP6500 dataset. X = Genic mutation rate calculated via the tri-mer mutation matrix, Y = common (>0.1% minor allele frequency) functional variants. **RVIS-YALL:** Based on the ESP6500 dataset. X = Genic mutation rate calculated via the tri-mer mutation matrix, Y = common (>0.1% minor allele frequency) protein-coding variants of all effects (including synonymous variants). **RVIS-CHGV:** Based on an internally sequenced cohort of 690 whole-genome sequenced samples. X = Genic mutation rate calculated via the tri-mer mutation matrix, Y = common (>1% minor allele frequency) functional variants. To obtain the presented levels of significance, we used a logistic regression model to regress the presence or absence of a gene, within the corresponding gene list, on each of the genic scores. The [95% CI] for the AUC estimates are provided for each cell. Scatter plots for the pairs of scores are available in S1 Fig.
(DOCX)

S3 Table Comparing the sub-regions of noncoding RVIS. Based on the intersecting 9,644 (57%) CCDS release 9 genes that had all the following RVIS “assessable” formulations: an original RVIS score (Petrovski et al 2013), an ncRVIS, a promoter ncRVIS, a 5’ UTR ncRVIS, and a 3’ UTR ncRVIS. To obtain the presented levels of significance, we used a logistic regression model to regress the presence or absence of a gene, within the corresponding gene list, on each of the genic scores. The [95% CI] for the AUC estimates are provided for each cell. Scatter plots for the pairs of scores are available in S1 Fig.
(DOCX)

S4 Table Joint logistic regression model to predict ClinGen’s dosage sensitive genes. This table contains the estimates achieved by six genic features in their ability to predict ClinGen’s dosage sensitive genes from the remaining exome background. See accompanying Fig 2.
(DOCX)

S5 Table Neuropsychiatric disorder ascertained loss-of-function de novo mutations. This table contains the loss-of-function de novo mutations found among a collection of neuropsychiatric disorder ascertained patients, that both occur in genes that are loss-of-function deficient and have a Euclidean distance from (0,0) ≤ 0.4.
(DOCX)

S1 Data Collection of RVIS and GERP scores and their corresponding percentiles. (XLSX)

S2 Data ClinGen Genome Dosage Map assessment. (XLSX)

S3 Data Tri-mer mutation rate calculator. (PL)

S4 Data Significantly loss-of-function (LoF) depleted gene list. (XLSX)

S5 Data Noncoding RVIS (ncRVIS) genomic boundaries. (TXT)
