# Supplementary material for: The Intolerance of Regulatory Sequence to Genetic Variation Predicts Gene Dosage Sensitivity
Source: PLoS Genet. 2015 Sep 2;11(9):e1005492. doi: 10.1371/journal.pgen.1005492 (PMC4557908; doi:10.1371/journal.pgen.1005492)
Supplement: S1 Fig — Scatter plots between different metrics and features used in this manuscript. S1A: ncRVIS and RVIS-CHGV; S1B: ncRVIS and noncoding mutation rate; S1C: ncRVIS and noncoding sequence size; S1D: ncRVIS and coding sequence size; S1E: ncRVIS and coding mutation rate; S1F: noncoding sequence size and noncoding mutation rate; S1G: RVIS and RVIS-mut; S1H: RVIS and RVIS-YALL; S1I: RVIS and RVIS-CHGV; S1J: ncGERP and pcGERP; S1K: ncRVIS and ncGERP; S1L: ncRVIS and pcGERP; S1M: RVIS and ncGERP; S1N: RVIS and pcGERP; S1O: ncRVIS and 3’ UTR ncRVIS; S1P: ncRVIS and promoter ncRVIS; S1Q: ncRVIS and 5’ UTR ncRVIS; S1R: RVIS and ncRVIS; S1S: ncCADD and noncoding sequence size; S1T: ncCADD and ncRVIS; S1U: ncCADD and RVIS-CHGV; S1V: ncCADD and ncGERP; S1W: ncCADD and ncGWAVA; S1X: ncGWAVA and noncoding sequence size; S1Y: ncGWAVA and ncRVIS; S1Z: ncGAVA and RVIS-CHGV; S1AA: ncGWAVA and ncGERP. (DOCX) [file pgen.1005492.s001.docx]

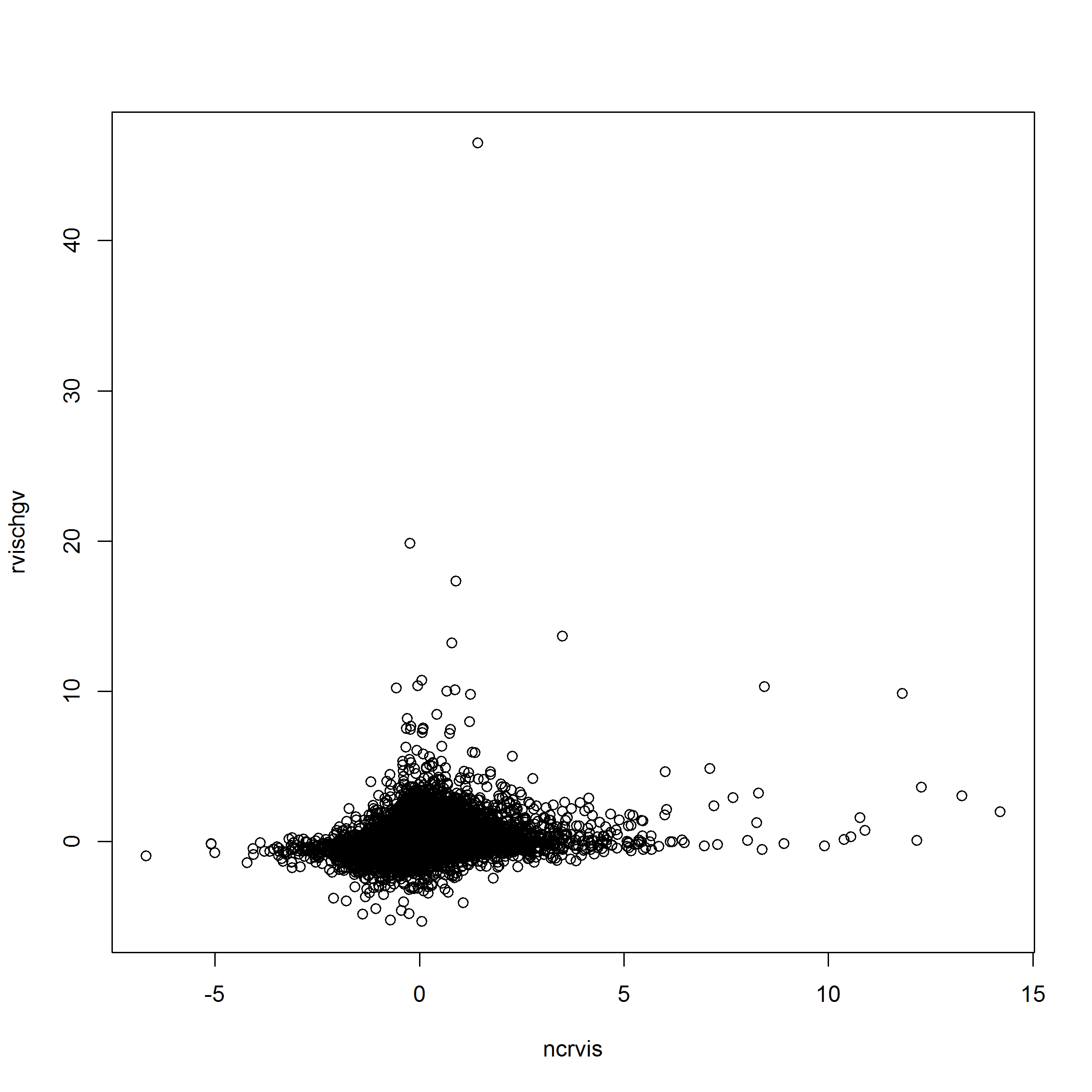
**
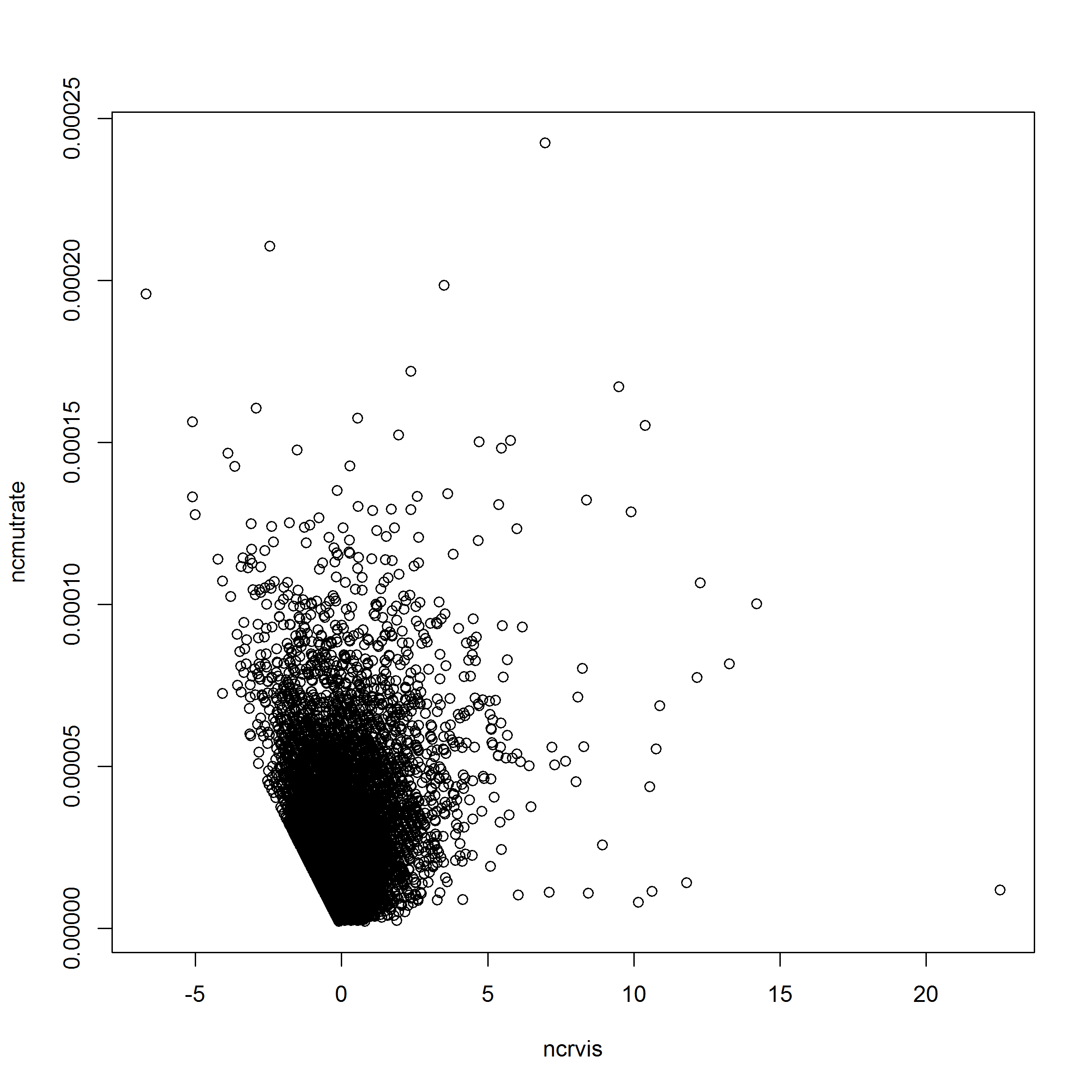

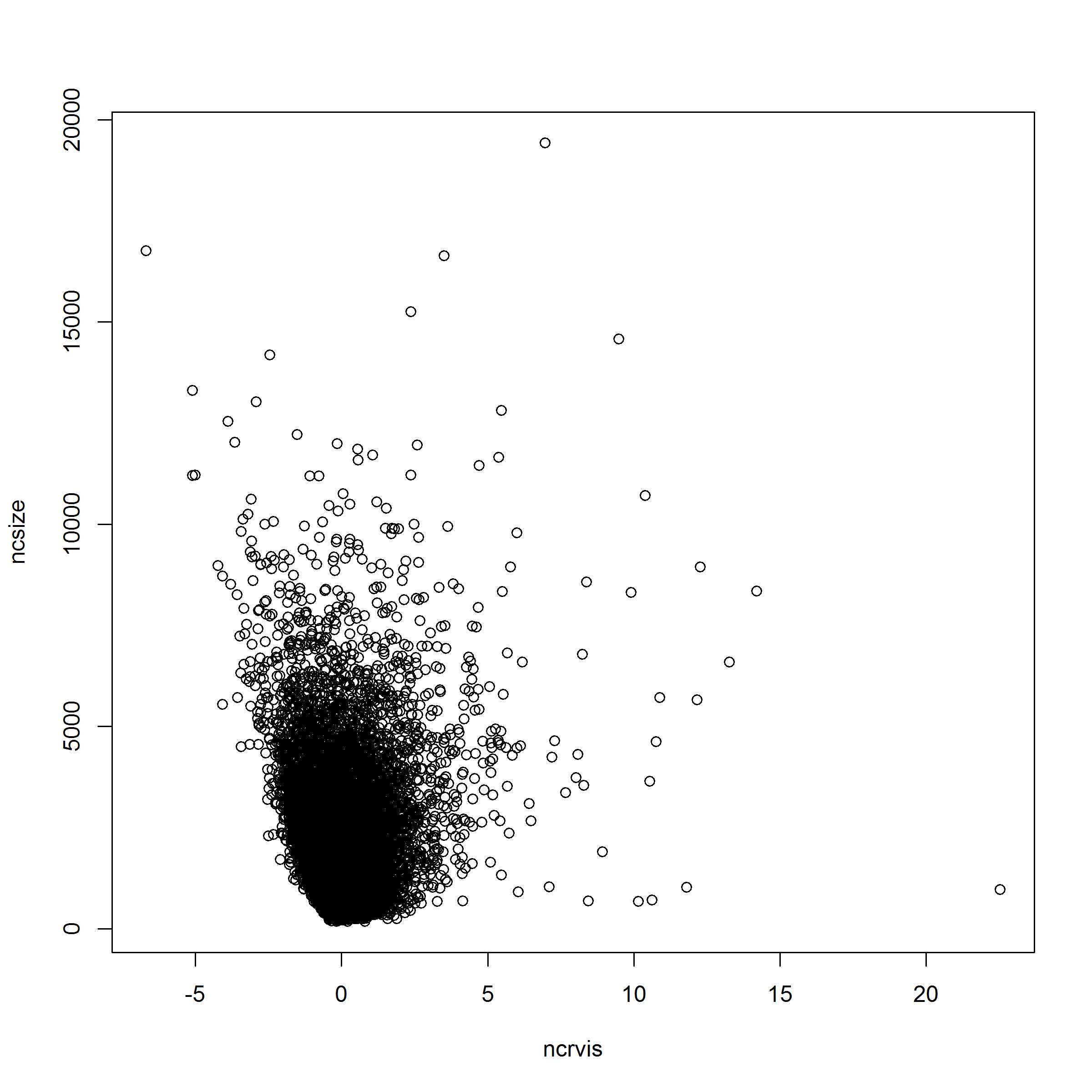

 A: ncRVIS and RVIS-CHGV B: ncRVIS and noncoding mutation rate C: ncRVIS and noncoding sequence size**

**n=15,471 assessable genes n=16,273 assessable genes n=16,273 assessable genes**

**(Spearman’s *r_s_* = 0.28; Pearson’s *r^2^* = 0.04) (Spearman’s *r_s_ = -0.15*; Pearson’s *r^2^* = 3x10^-8^) (Spearman’s *r_s_ = -0.12*; Pearson’s *r^2^* = 2x10^-5^)**

**
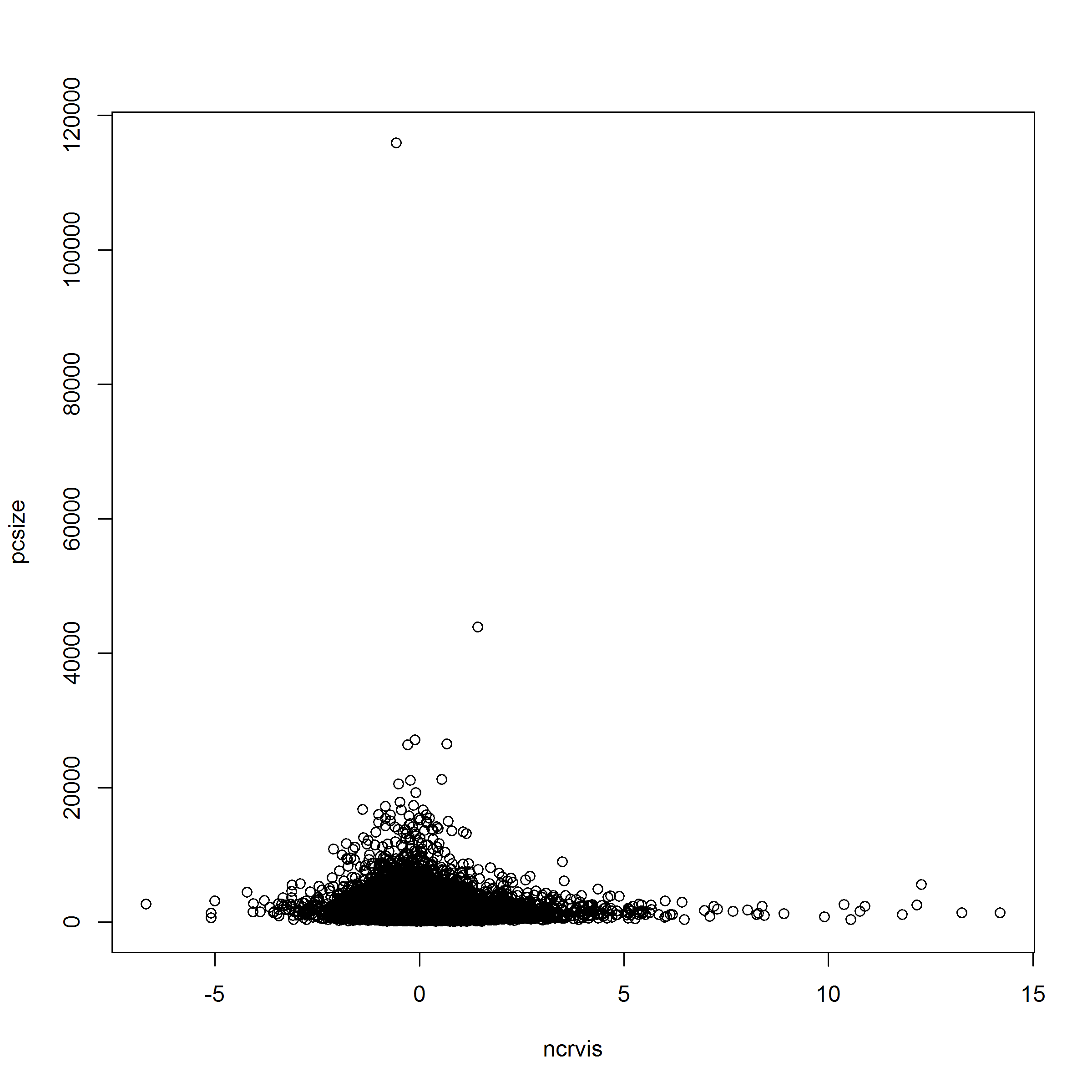
**
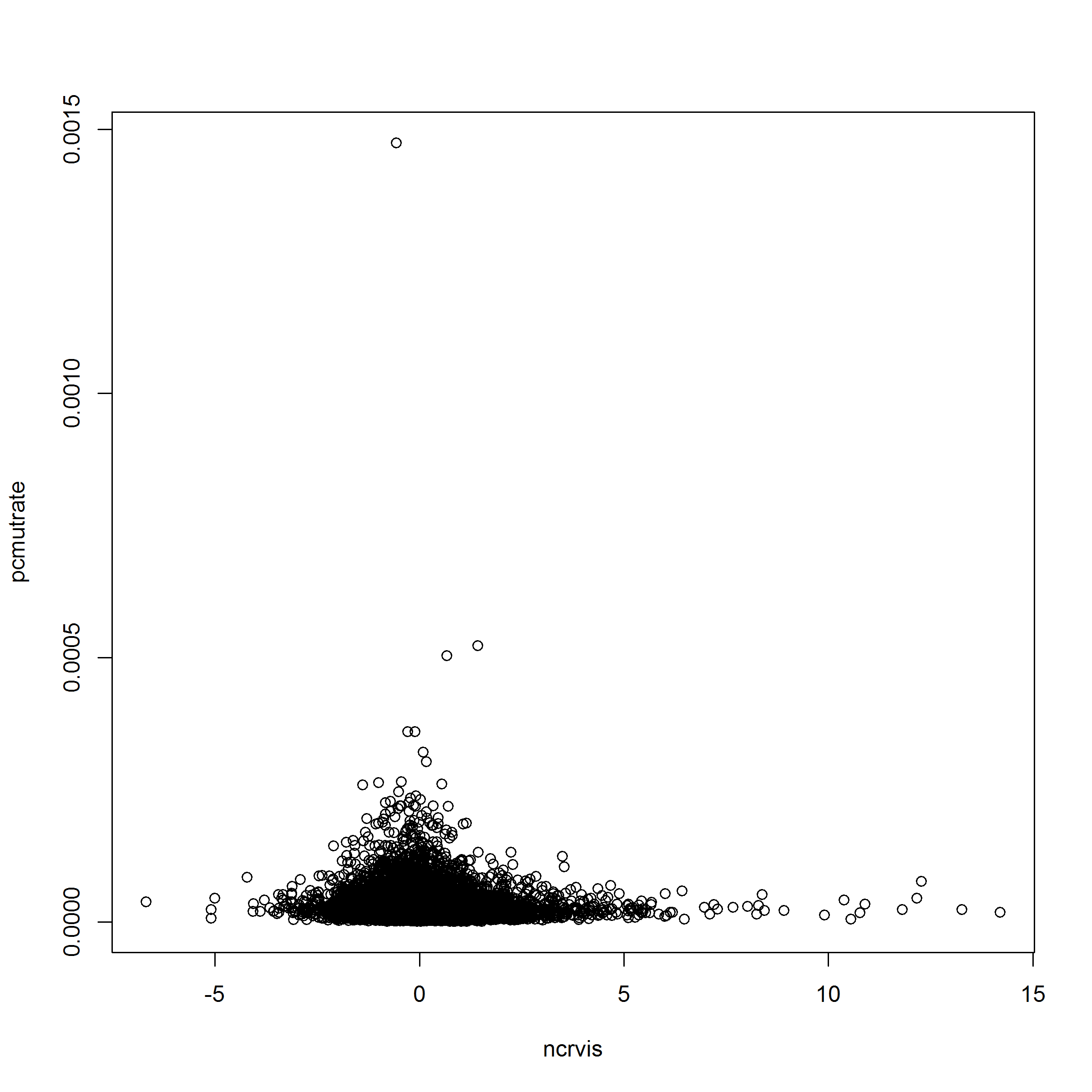
 **
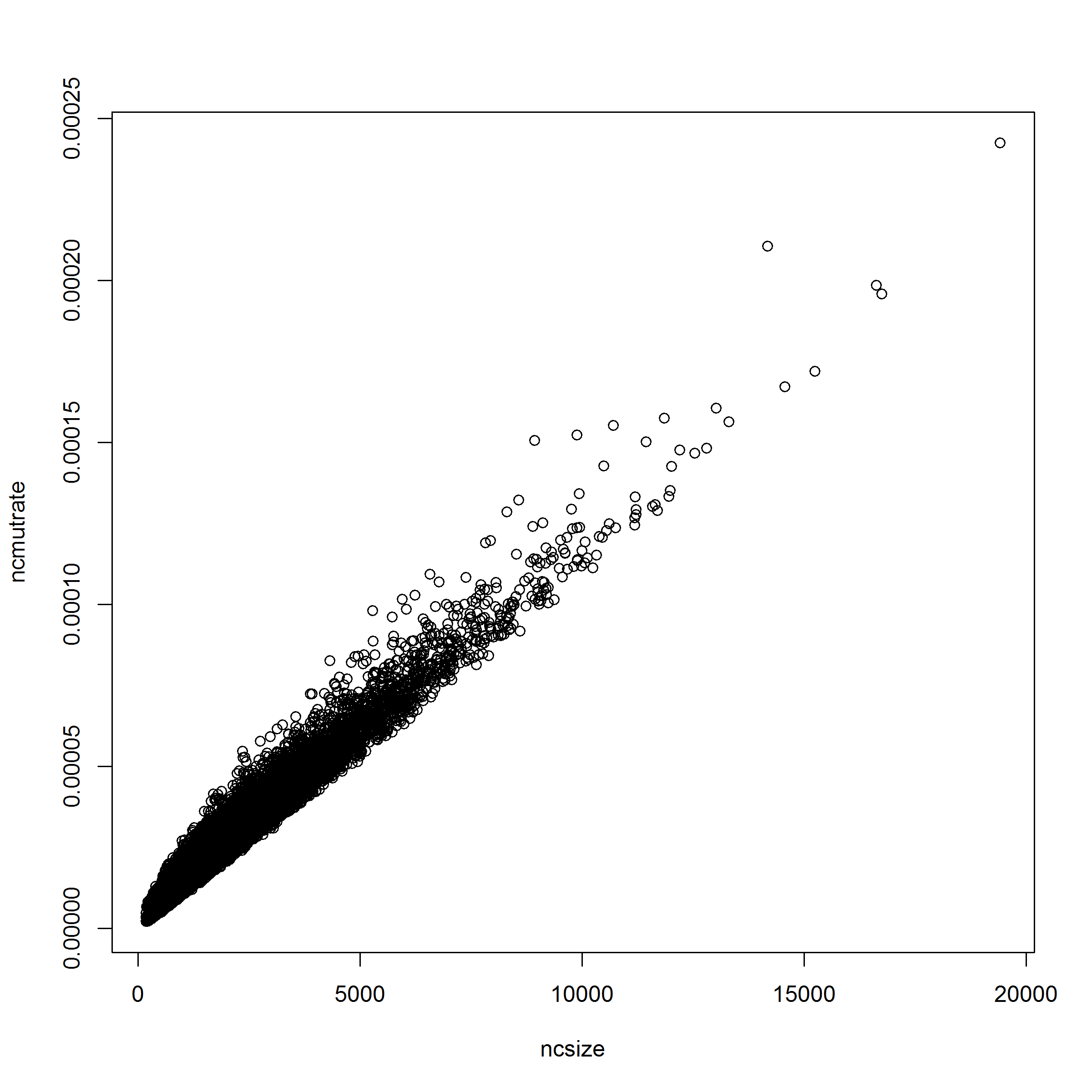
**

**D: ncRVIS and coding sequence size E: ncRVIS and coding mutation rate F: noncoding sequence size and mutation rate**

**n=15,471 assessable genes n=15,471 assessable genes n=16,273 assessable genes**

**(Spearman’s *r_s_* = -0.09; Pearson’s *r^2^* = 0.003) (Spearman’s *r_s_* = -0.10; Pearson’s *r^2^* = 0.003) (Spearman’s *r_s_* = 0.97; Pearson’s *r^2^* = 0.949)**

**
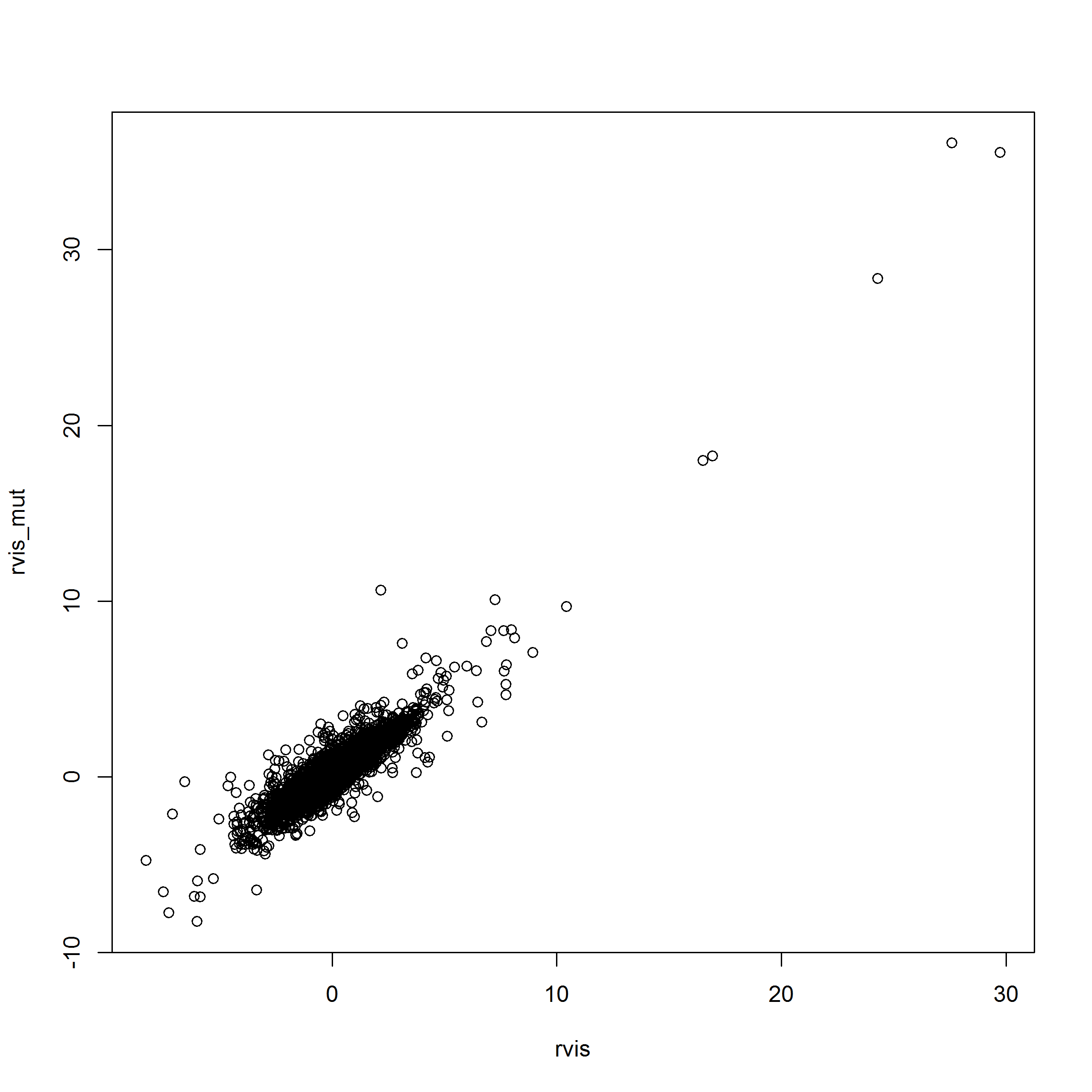

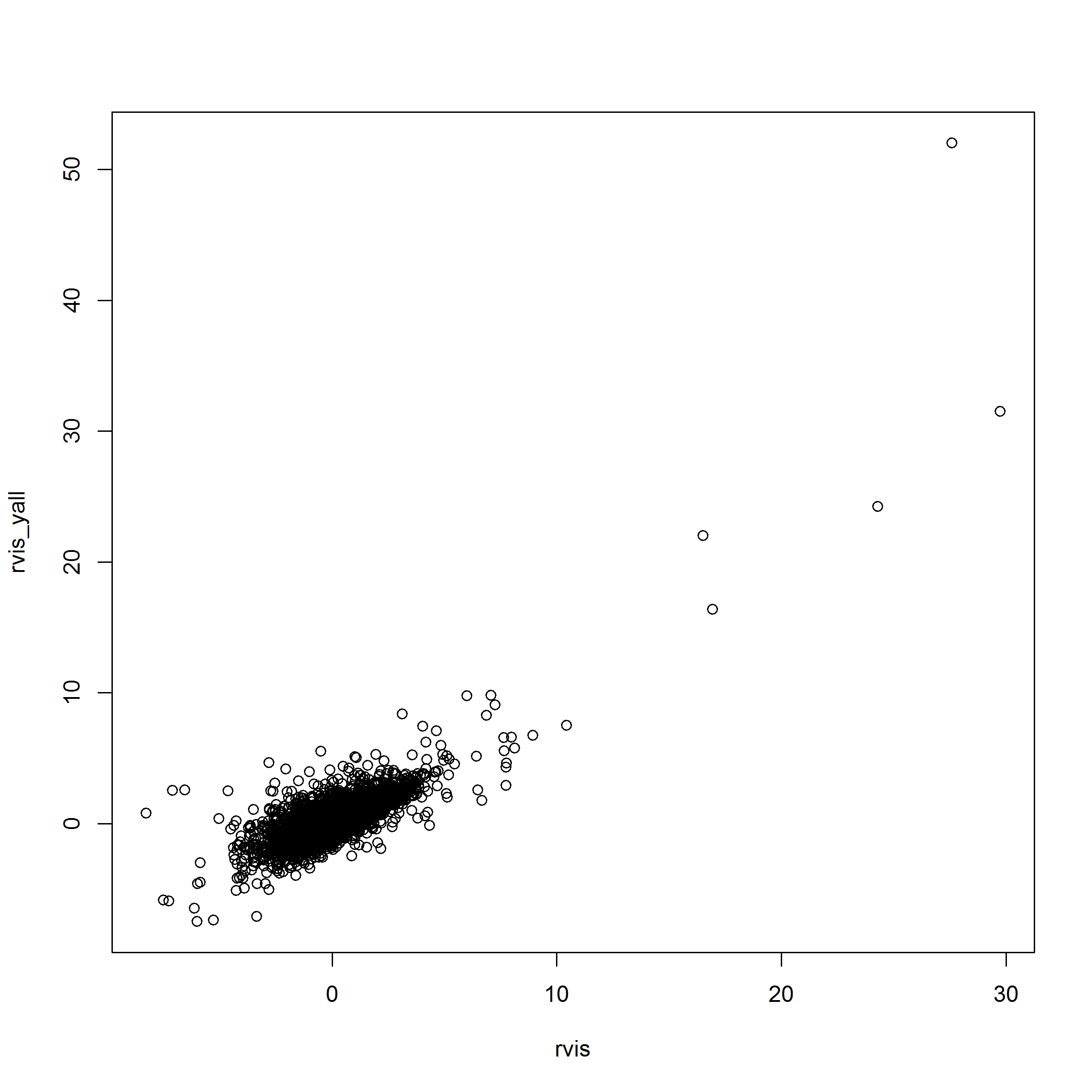

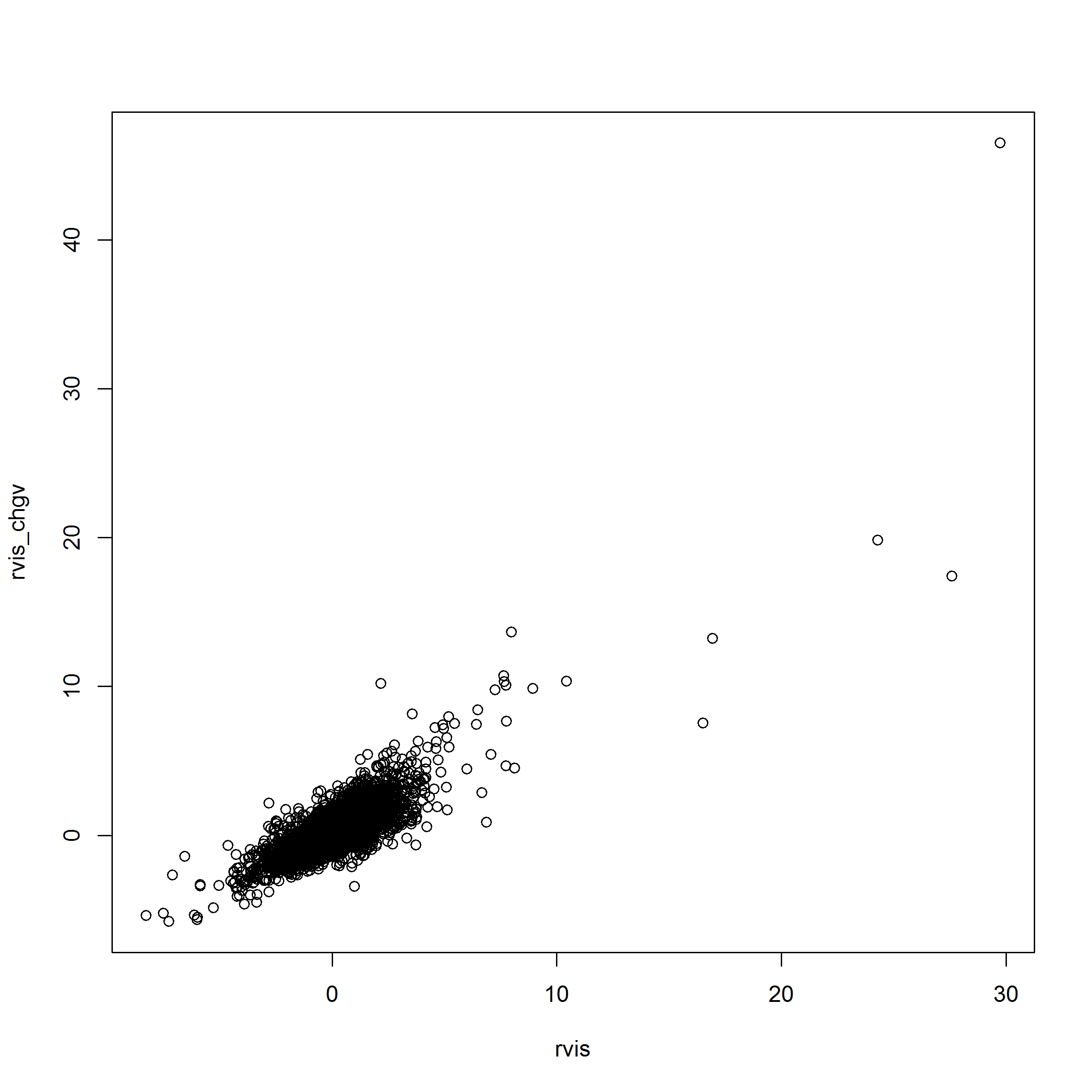
**

**G: RVIS and RVIS-mut H: RVIS and RVIS-YALL I: RVIS and RVIS-CHGV**

**n=16,275 assessable genes n=16,275 assessable genes n=16,275 assessable genes**

**(Spearman’s *r_s_* = 0.88; Pearson’s *r^2^* = 0.831) (Spearman’s *r_s_* = 0.68; Pearson’s *r^2^* = 0.588) (Spearman’s *r_s_* = 0.71; Pearson’s *r^2^* = 0.634)**

**
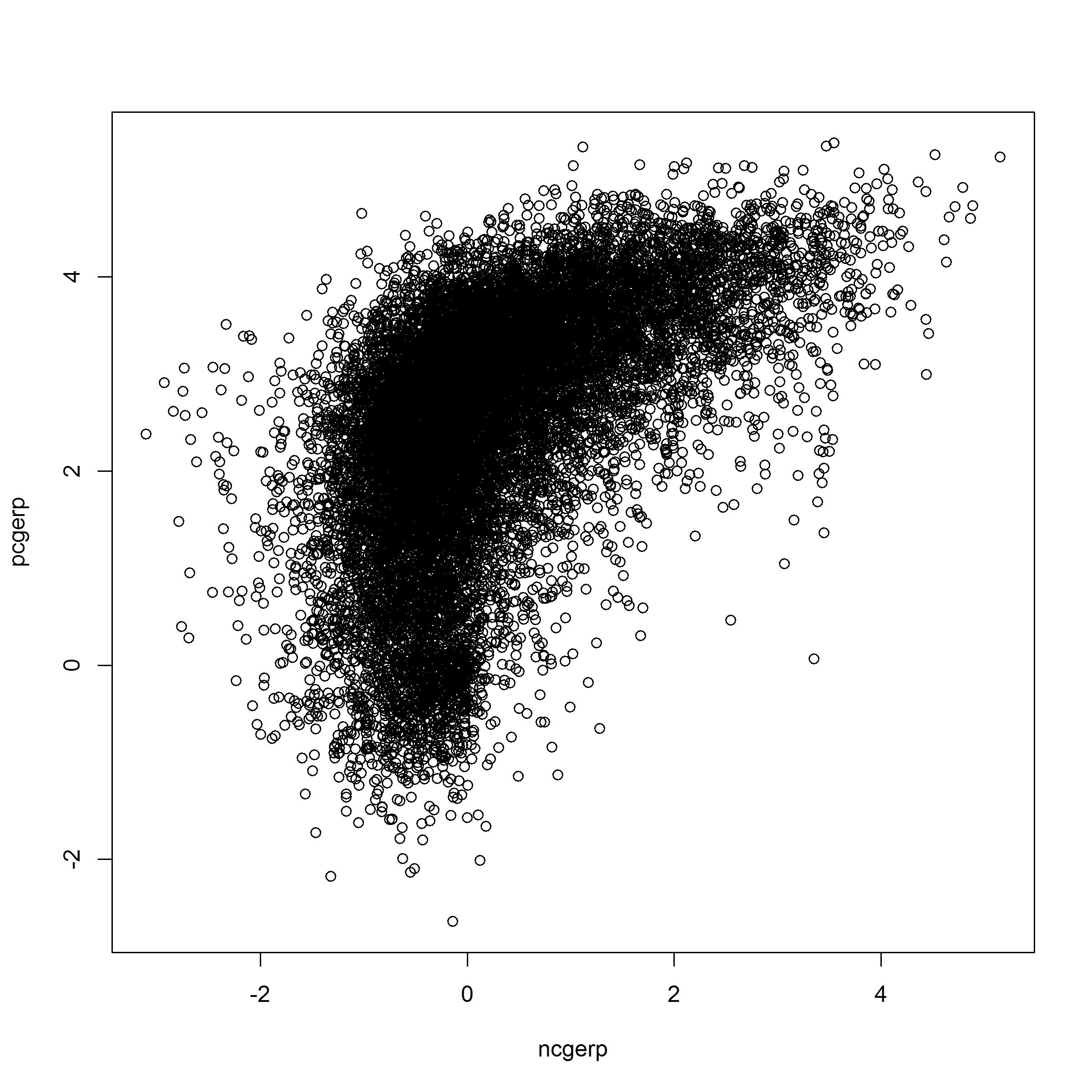

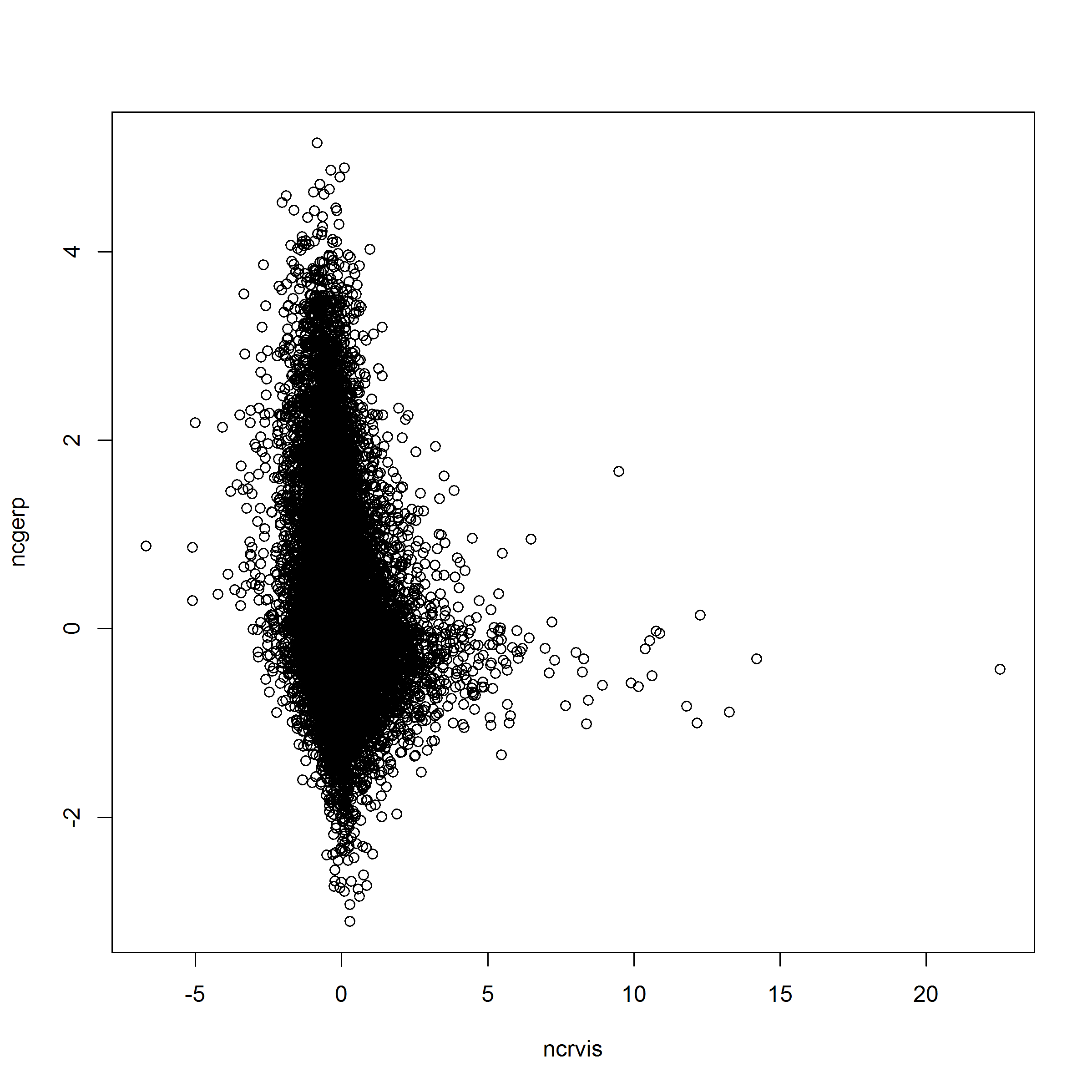

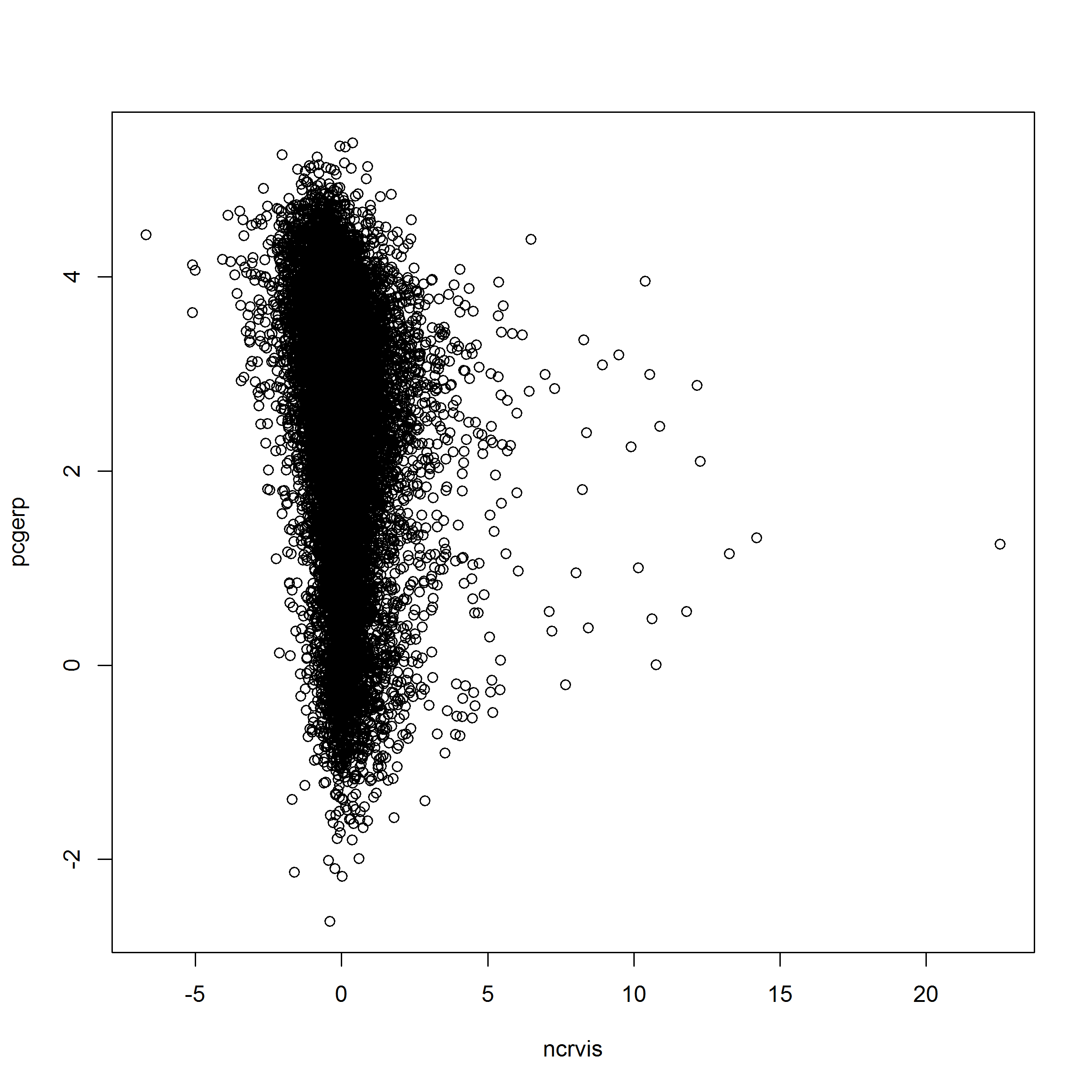
**

**J: ncGERP and pcGERP K: ncRVIS and ncGERP L: ncRVIS and pcGERP**

**n=17,989 assessable genes n=16,214 assessable genes n=16,189 assessable genes**

**(Spearman’s *r_s_* = 0.59; Pearson’s *r^2^* = 0.297) (Spearman’s *r_s_* = -0.30; Pearson’s *r^2^* = 0.064) (Spearman’s *r_s_* = -0.26; Pearson’s *r^2^* = 0.040)**


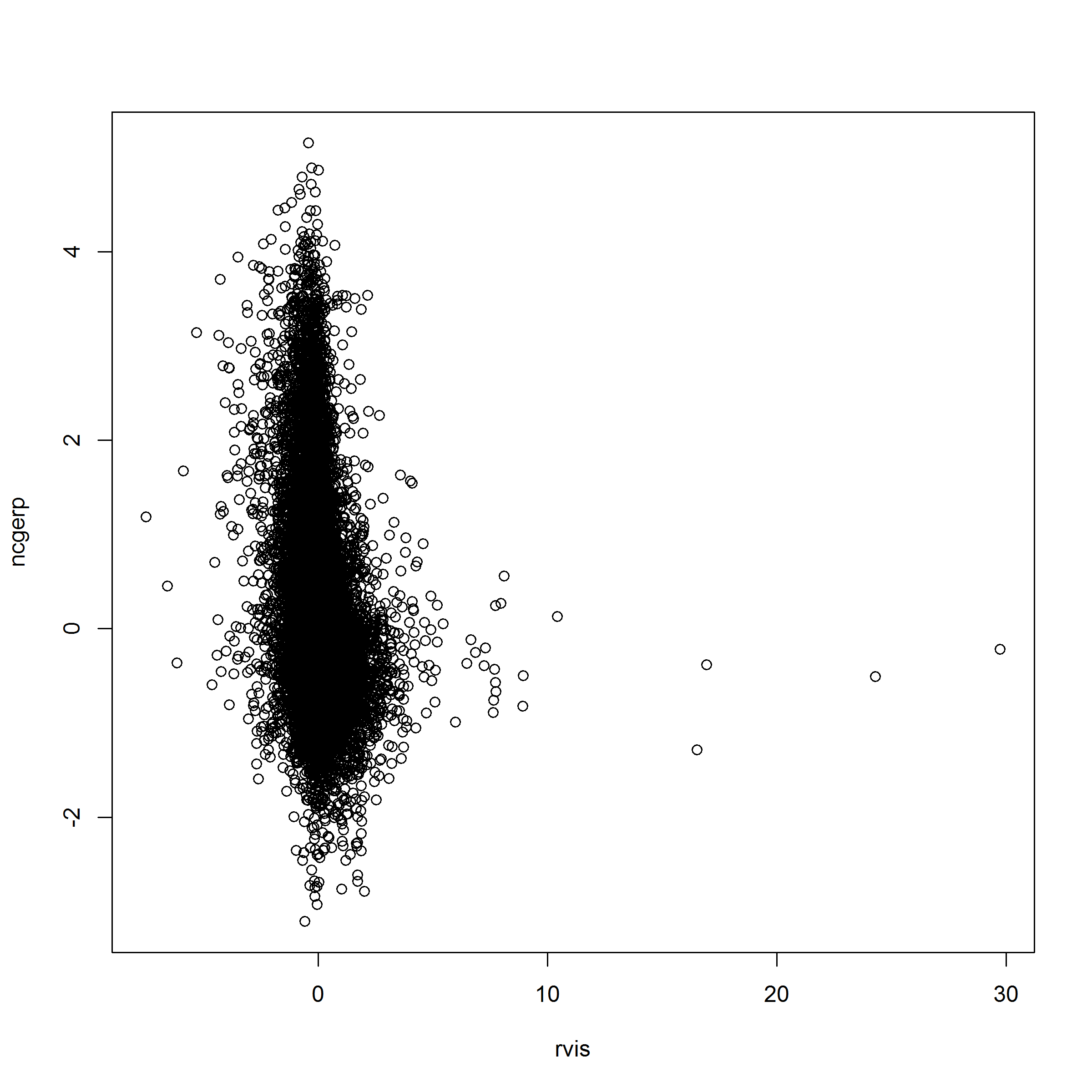

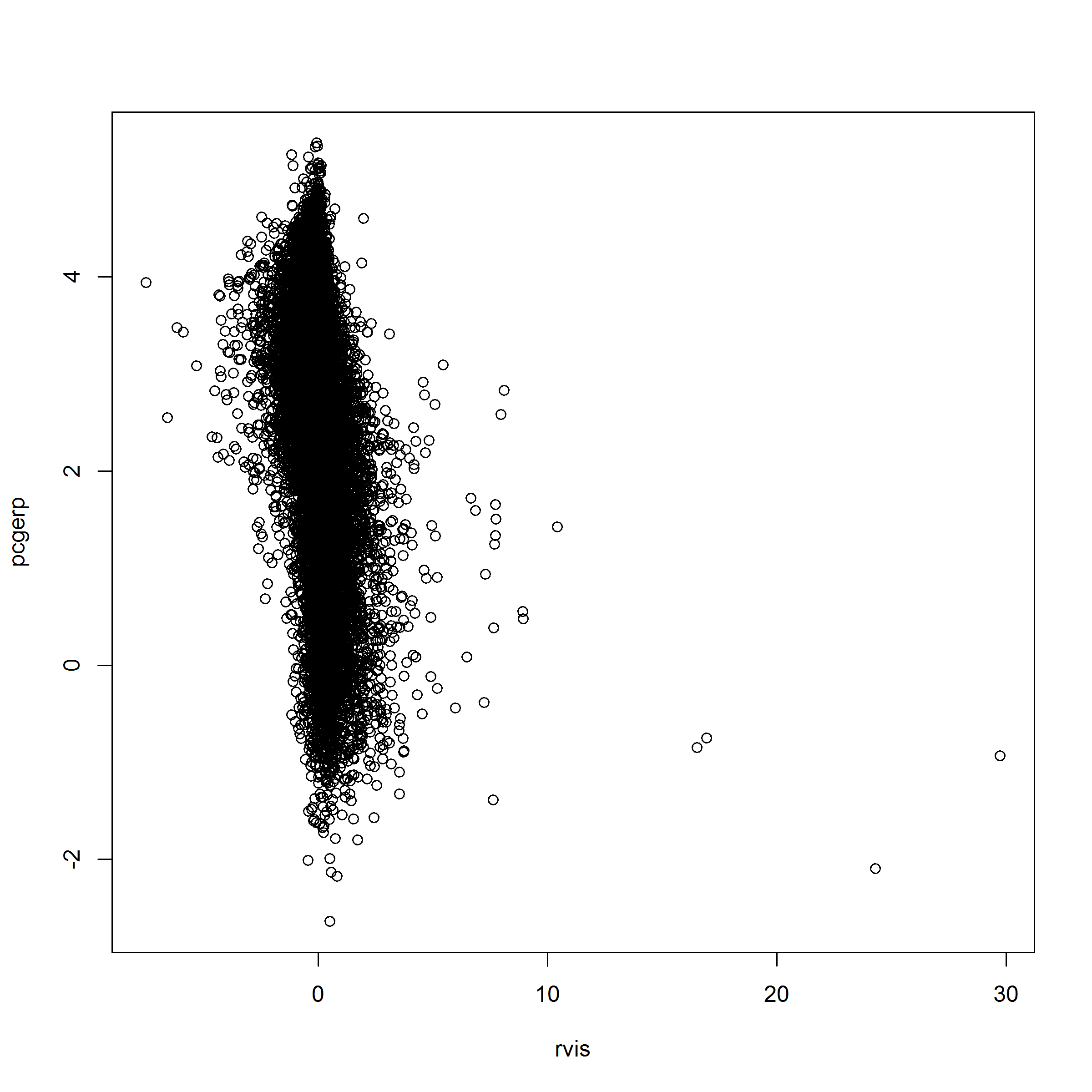

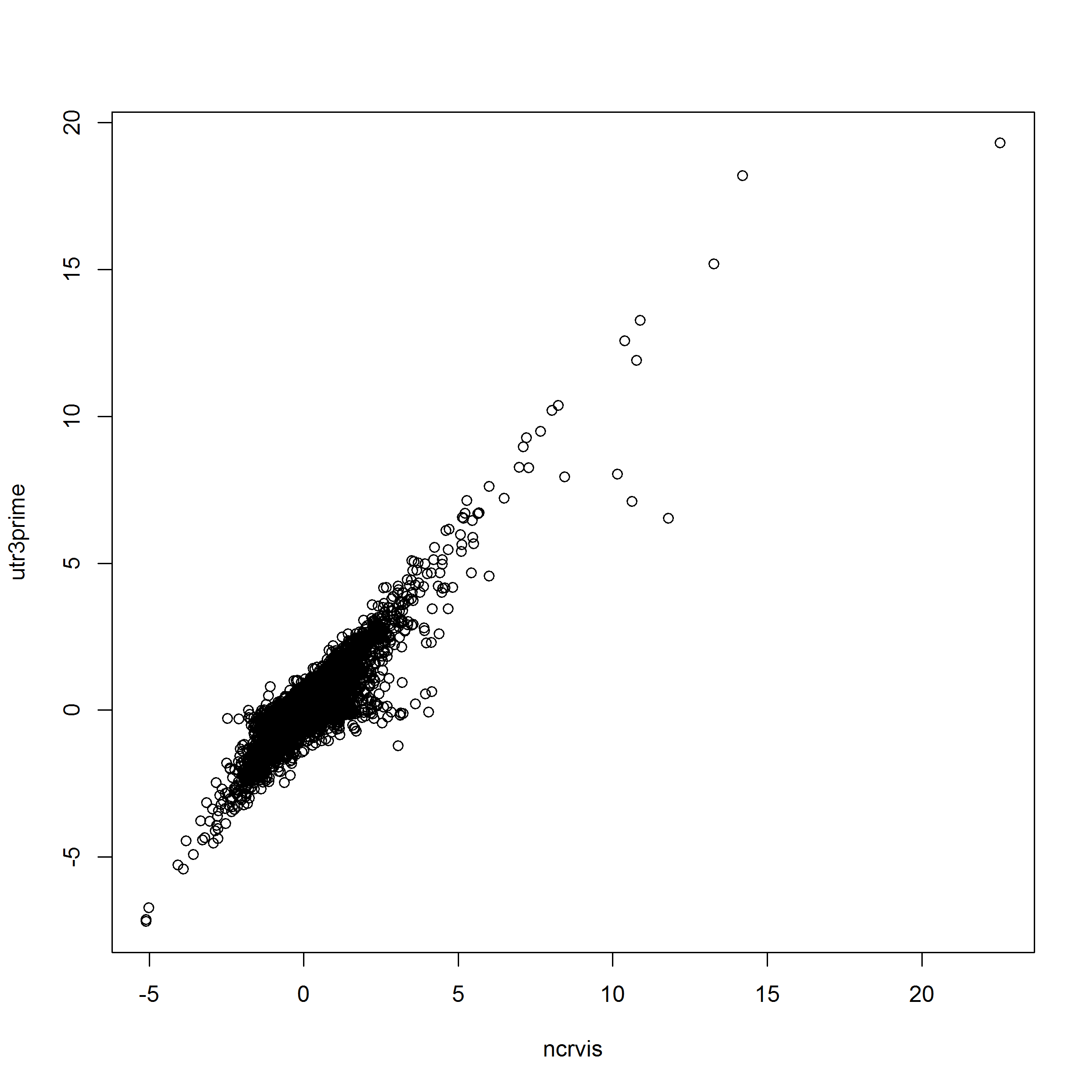


**M: RVIS and ncGERP N: RVIS and pcGERP O: ncRVIS and 3’ UTR ncRVIS**

**n=16,736 assessable genes n=16,708 assessable genes n=9,644 assessable genes**

**(Spearman’s *r_s_* = -0.29; Pearson’s *r^2^* = 0.063) (Spearman’s *r_s_* = -0.44; Pearson’s *r^2^* = 0.146) (Spearman’s *r_s_* = 0.78; Pearson’s *r^2^* = 0.790)**


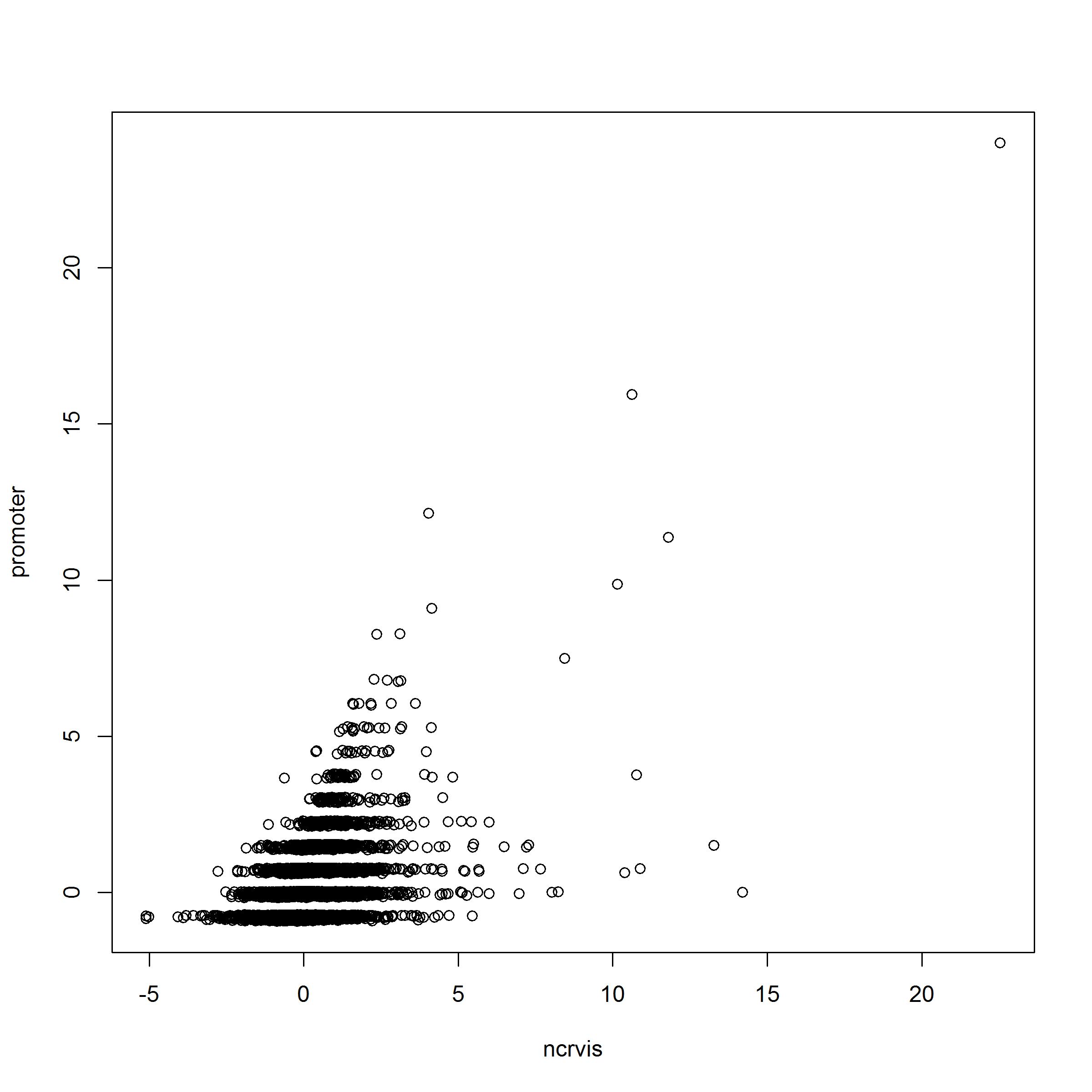

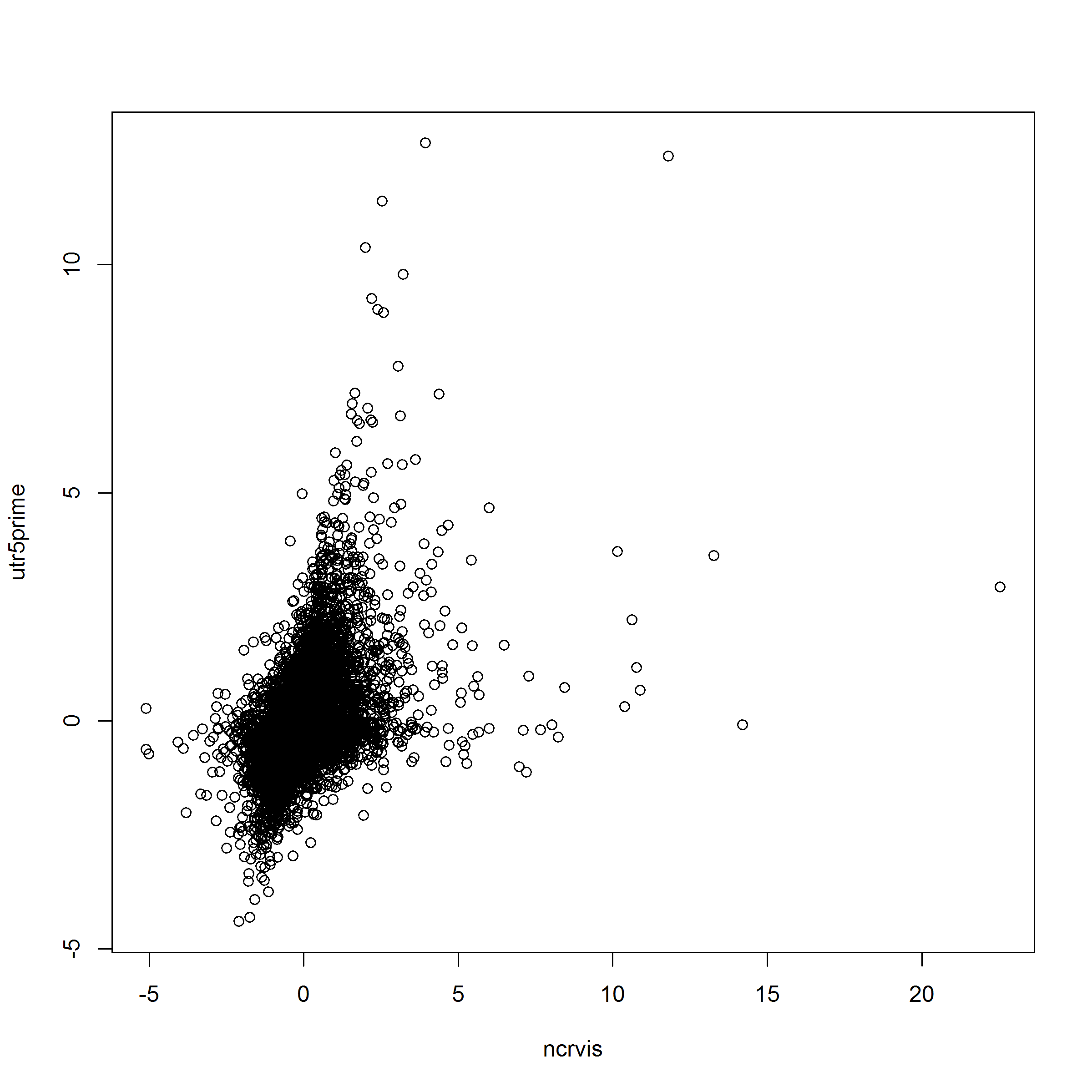

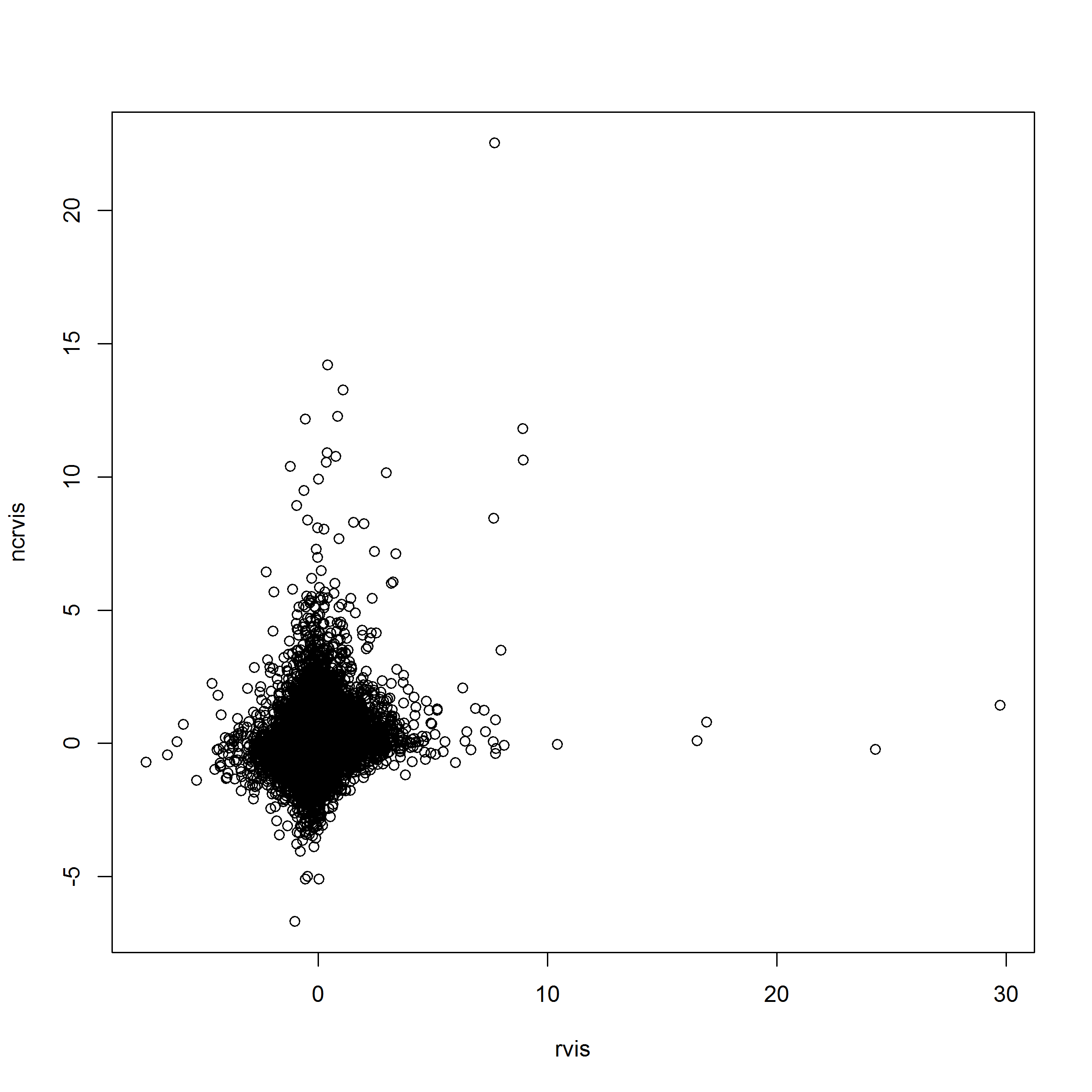


**P: ncRVIS and promoter ncRVIS Q: ncRVIS and 5’ UTR ncRVIS R: RVIS and ncRVIS**

**n=9,644 assessable genes n=9,644 assessable genes n=15,034 assessable genes**

**(Spearman’s *r_s_* = 0.52; Pearson’s *r^2^* = 0.247) (Spearman’s *r_s_* = 0.48; Pearson’s *r^2^* = 0.197) (Spearman’s *r_s_* = 0.20; Pearson’s *r^2^* = 0.028)**


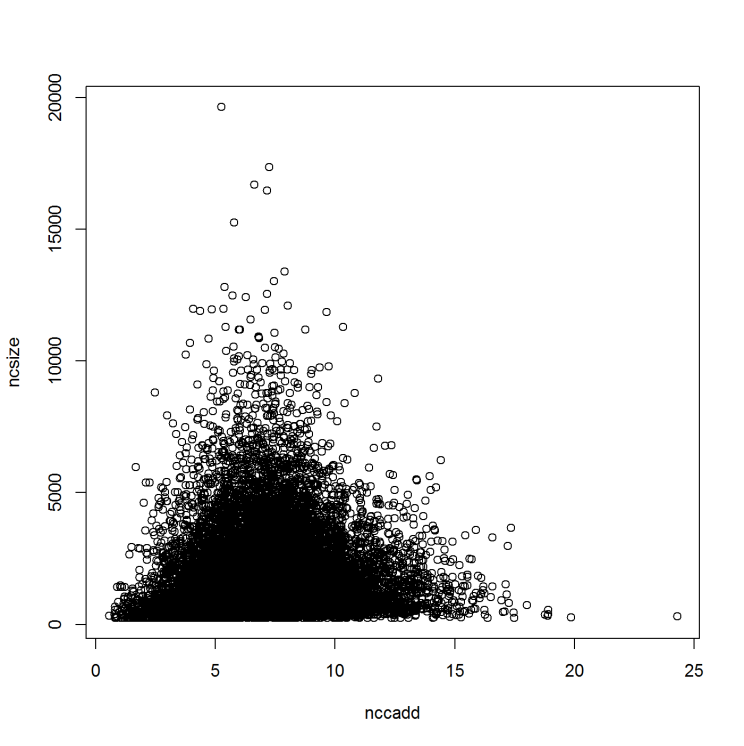

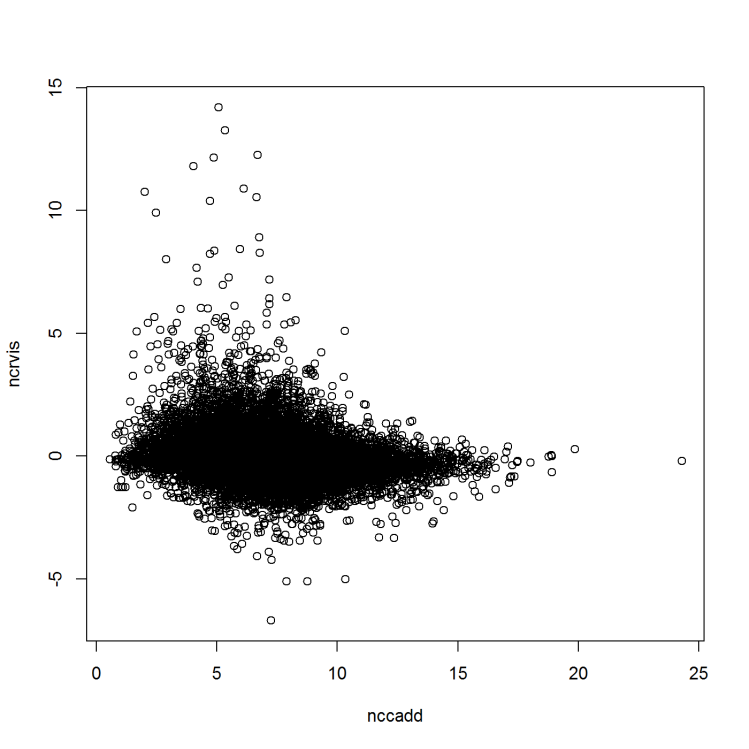
**
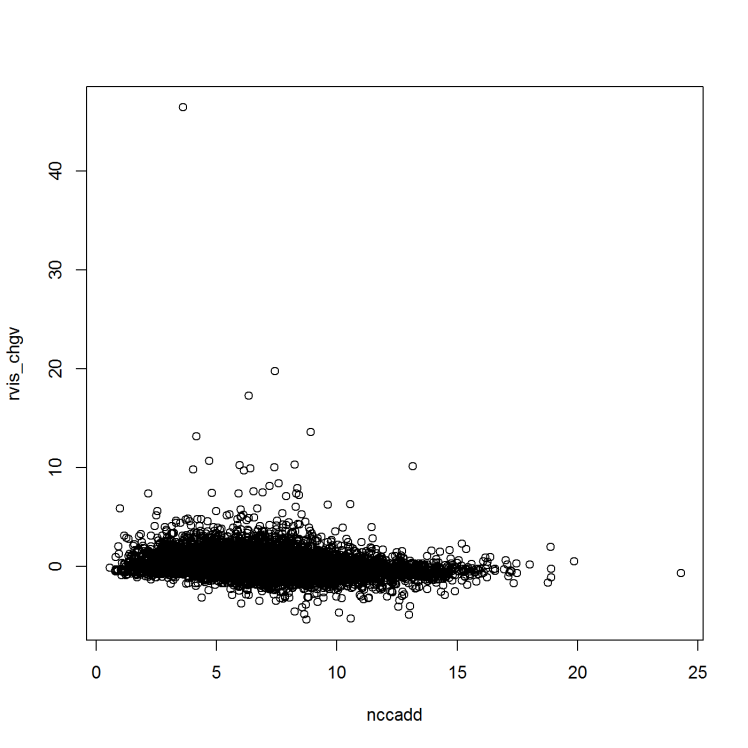
**

**S: ncCADD and noncoding sequence size T: ncCADD and ncRVIS U: ncCADD and RVIS-CHGV**

**n=15,420 assessable genes n=15,420 assessable genes n=15,420 assessable genes**

**(Spearman’s *r_s_* = 0.04; Pearson’s *r^2^* = 4.6x10^-5^) (Spearman’s *r_s_* = -0.25; Pearson’s *r^2^* = 0.050) (Spearman’s *r_s_* = -0.30; Pearson’s *r^2^* = 0.057)**


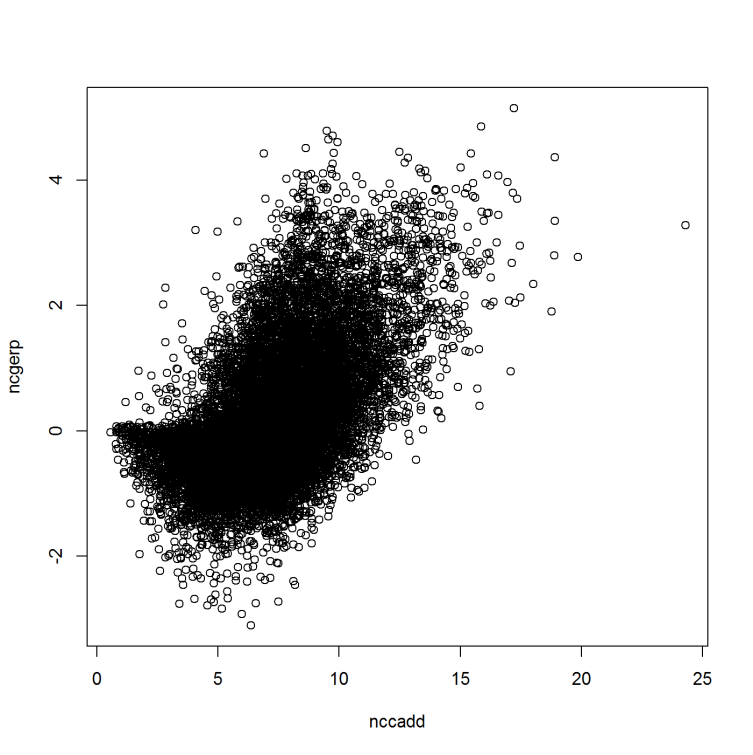

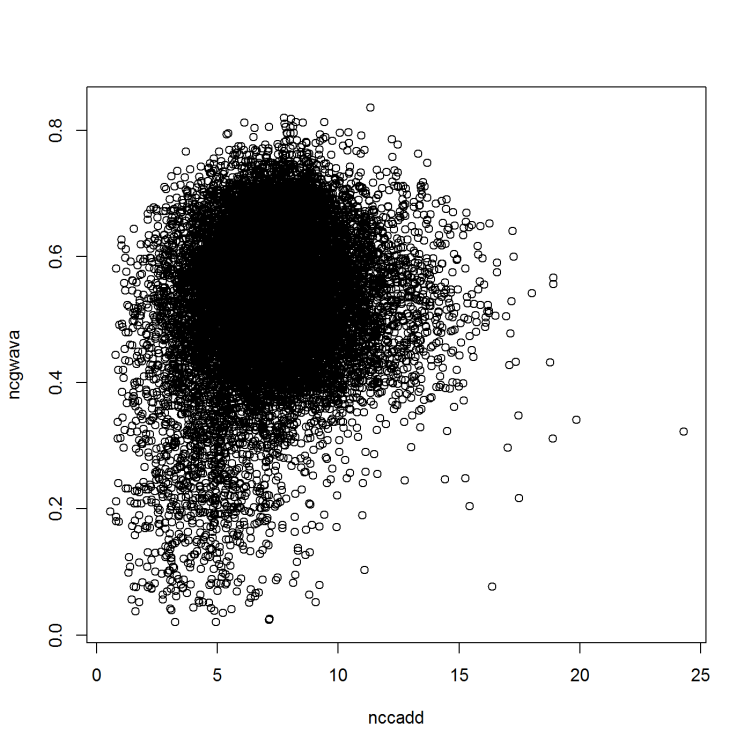

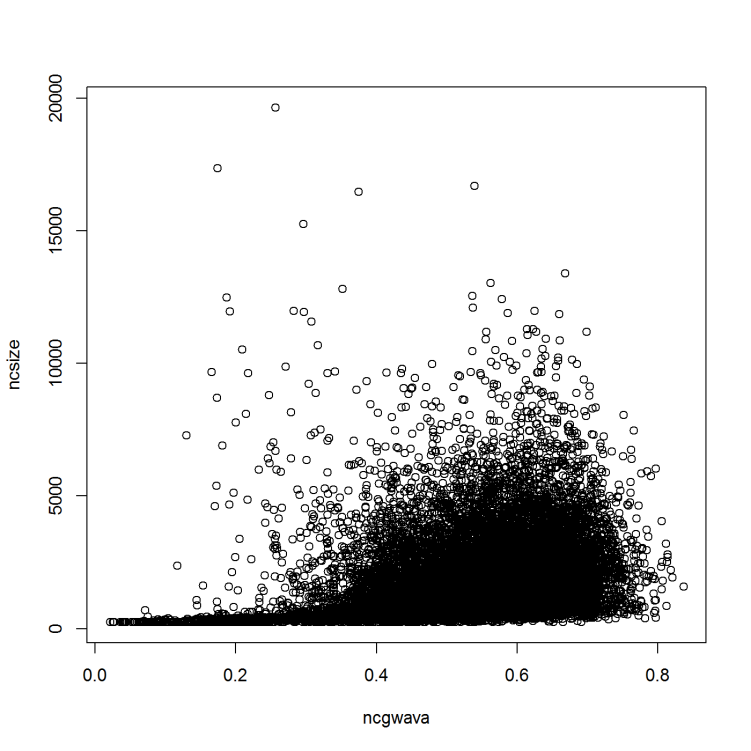


**V: ncCADD and ncGERP W: ncCADD and ncGWAVA X: ncGWAVA and noncoding sequence size**

**n=15,420 assessable genes n=15,420 assessable genes n=15,420 assessable genes**

**(Spearman’s *r_s_* = 0.55; Pearson’s *r^2^* = 0.315) (Spearman’s *r_s_* = 0.14; Pearson’s *r^2^* = 0.032) (Spearman’s *r_s_* = 0.39; Pearson’s *r^2^* = 0.069)**


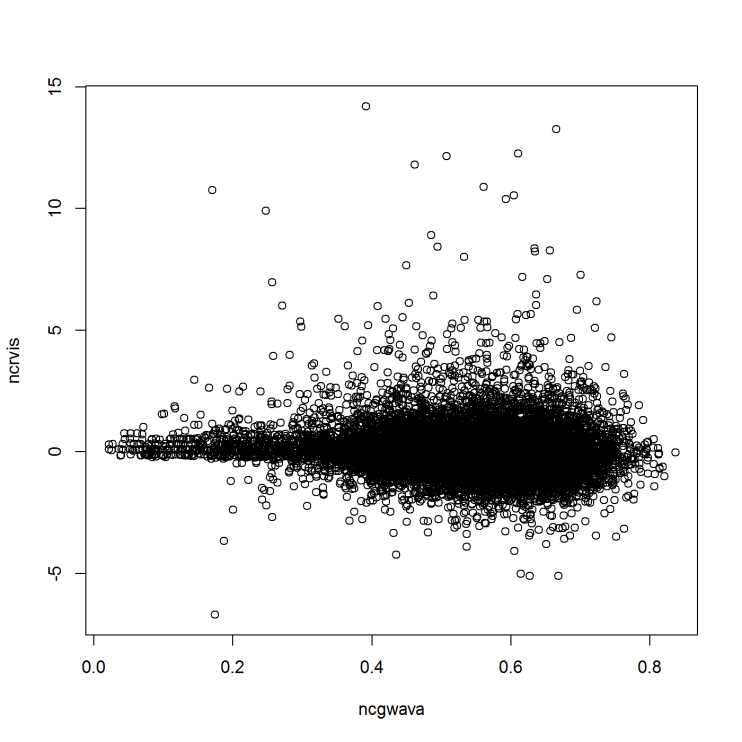

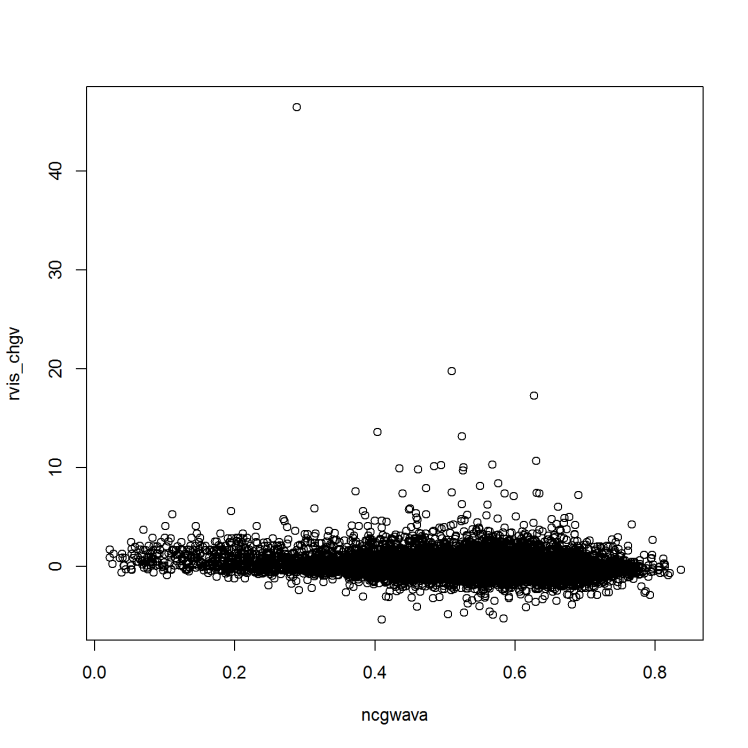

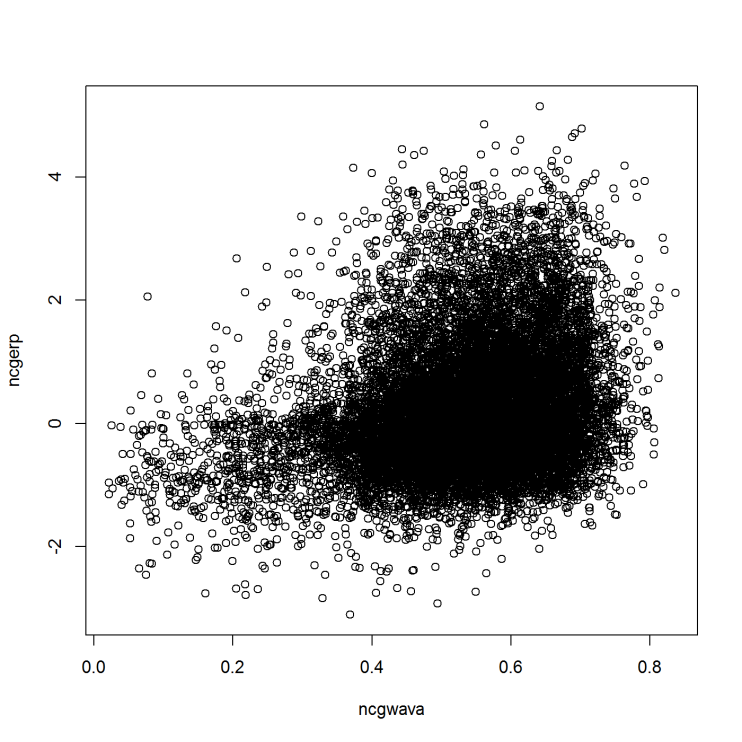


**Y: ncGWAVA and ncRVIS Z: ncGWAVA and RVIS-CHGV AA: ncGWAVA and ncGERP**

**n=15,420 assessable genes n=15,420 assessable genes n=15,420 assessable genes**

**(Spearman’s *r_s_* = -0.10; Pearson’s *r^2^* = 0.006) (Spearman’s *r_s_* = -0.17; Pearson’s *r^2^* = 0.029) (Spearman’s *r_s_* = 0.24; Pearson’s *r^2^* = 0.064)**
