# Supplementary figures and images for: The Intolerance of Regulatory Sequence to Genetic Variation Predicts Gene Dosage Sensitivity
Source: PLoS Genet. 2015 Sep 2;11(9):e1005492. doi: 10.1371/journal.pgen.1005492 (PMC4557908; doi:10.1371/journal.pgen.1005492)

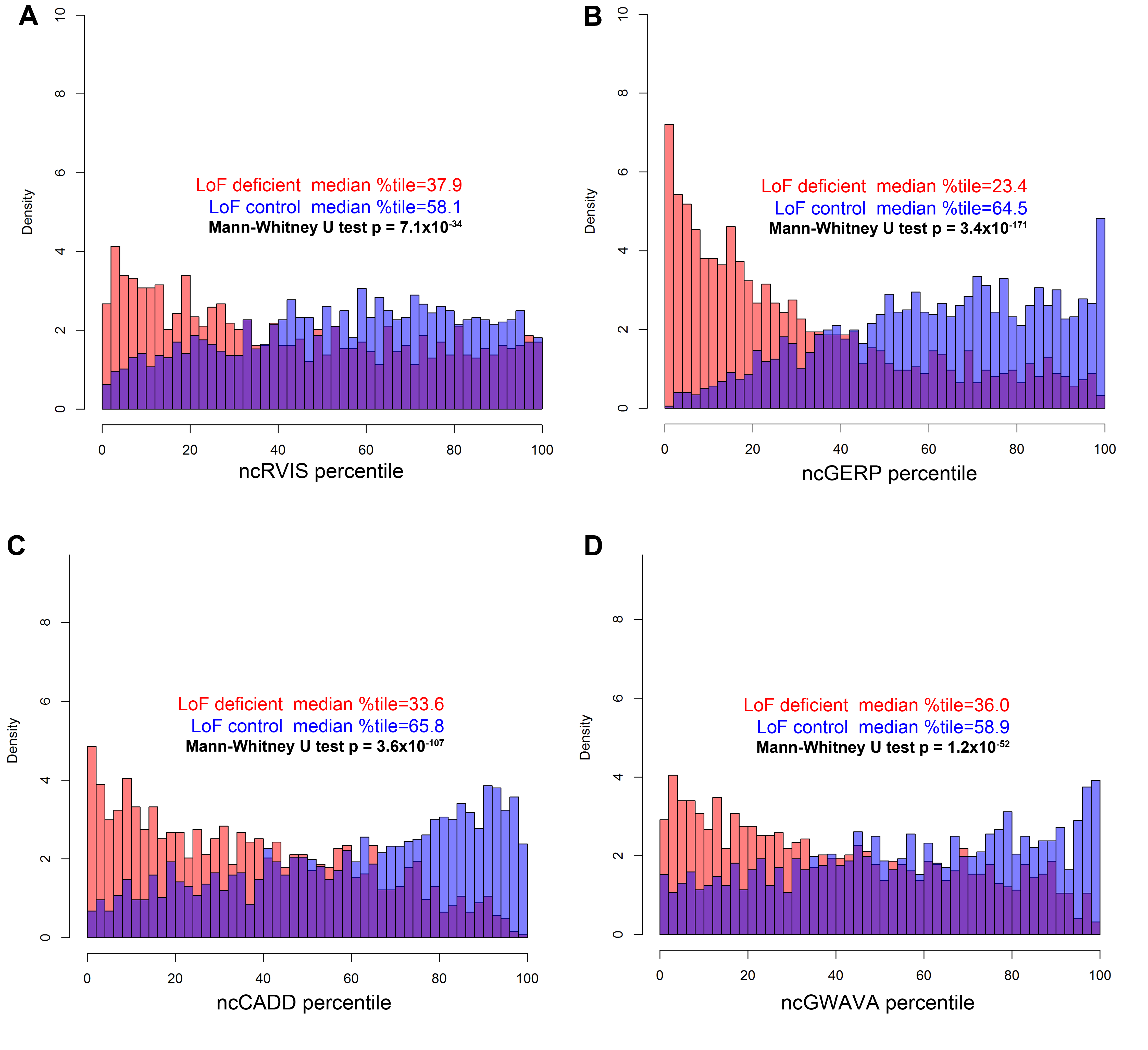

Supplement: S2 Fig — The distribution of genic score percentiles for the 1,235 LoF deficient (red distribution) compared to the 1,762 LoF control (blue distribution) genes. (A) ncRVIS percentiles, (B) ncGERP percentiles, (C) ncCADD percentiles and (D) ncGWAVA percentiles. (TIF) [file pgen.1005492.s002.tif]

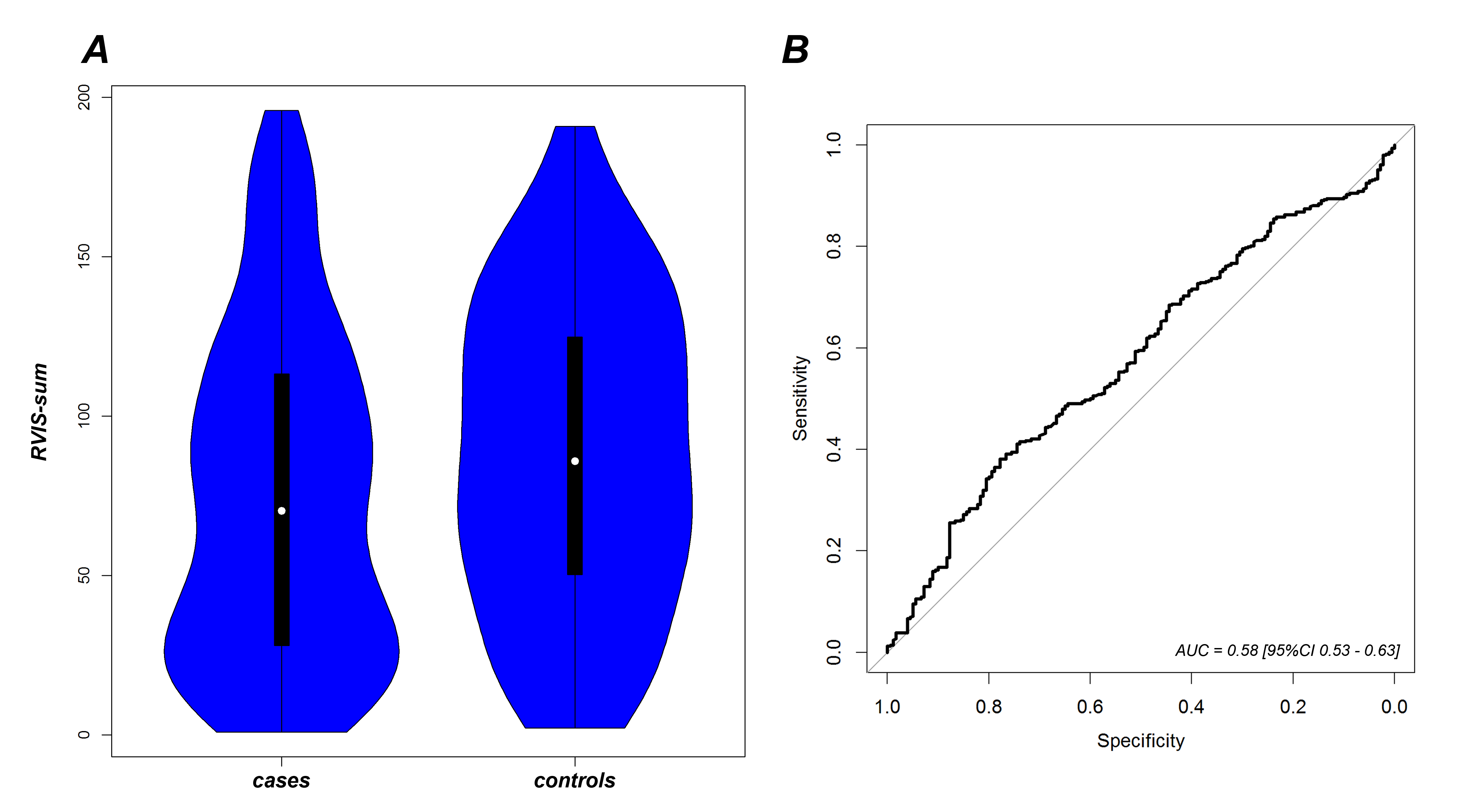

Supplement: S3 Fig — (A) Distribution of RVIS-sum scores for genes affected by loss-of-function de novo mutations. A median RVIS-sum score of 70.3 observed among 494 case-ascertained de novo mutations and a median of 85.9 among 180 de novo mutations from controls not ascertained for a neuropsychiatric disorder (Mann-Whitney U test p = 1.5x10^-3^). (B) Receiver operating characteristic (ROC) curve measuring the ability of the Euclidean distance for each LoF de novo mutation to discriminate between case and control ascertained LoF DNMs (AUC = 0.58 [95% CI 0.53–0.63]). (TIF) [file pgen.1005492.s003.tif]

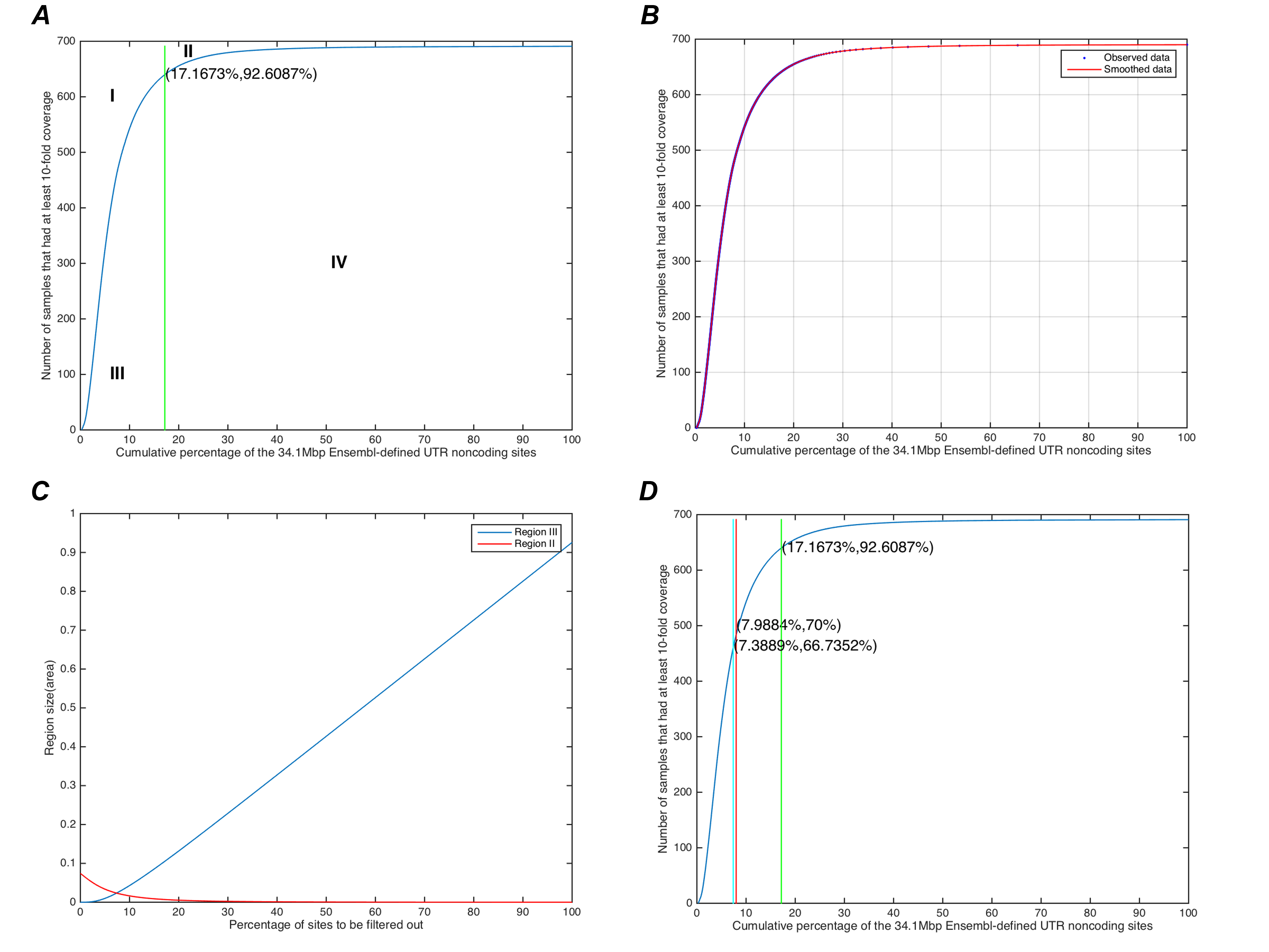

Supplement: S4 Fig — In S4A, S4B, S4D Figures the blue curve represents the number of our 690 whole-genome sequenced samples that had at least 10-fold coverage (Y-axis) versus the cumulative percentage of the 34.1Mbp Ensembl-defined UTR noncoding sites (X-axis). For example, in S4A Fig the intersection between the blue curve and green line (an illustrative cutoff) indicates that at this point approximately 92% of samples have at least 10-fold read coverage at approximately 83% of the Ensembl noncoding sites, and less than 10-fold coverage at approximately 17% of the Ensembl noncoding sites. S4C Fig represents the area of region II (red) and region III (blue in S4A Fig) for different X-axis cutoffs. The optimal threshold we decide to use as an x-axis cut-off in S4A Fig is selected by finding the intersection between the blue and red curves in S4C Fig–as represented by the red line in S4D Fig. (TIF) [file pgen.1005492.s004.tif]

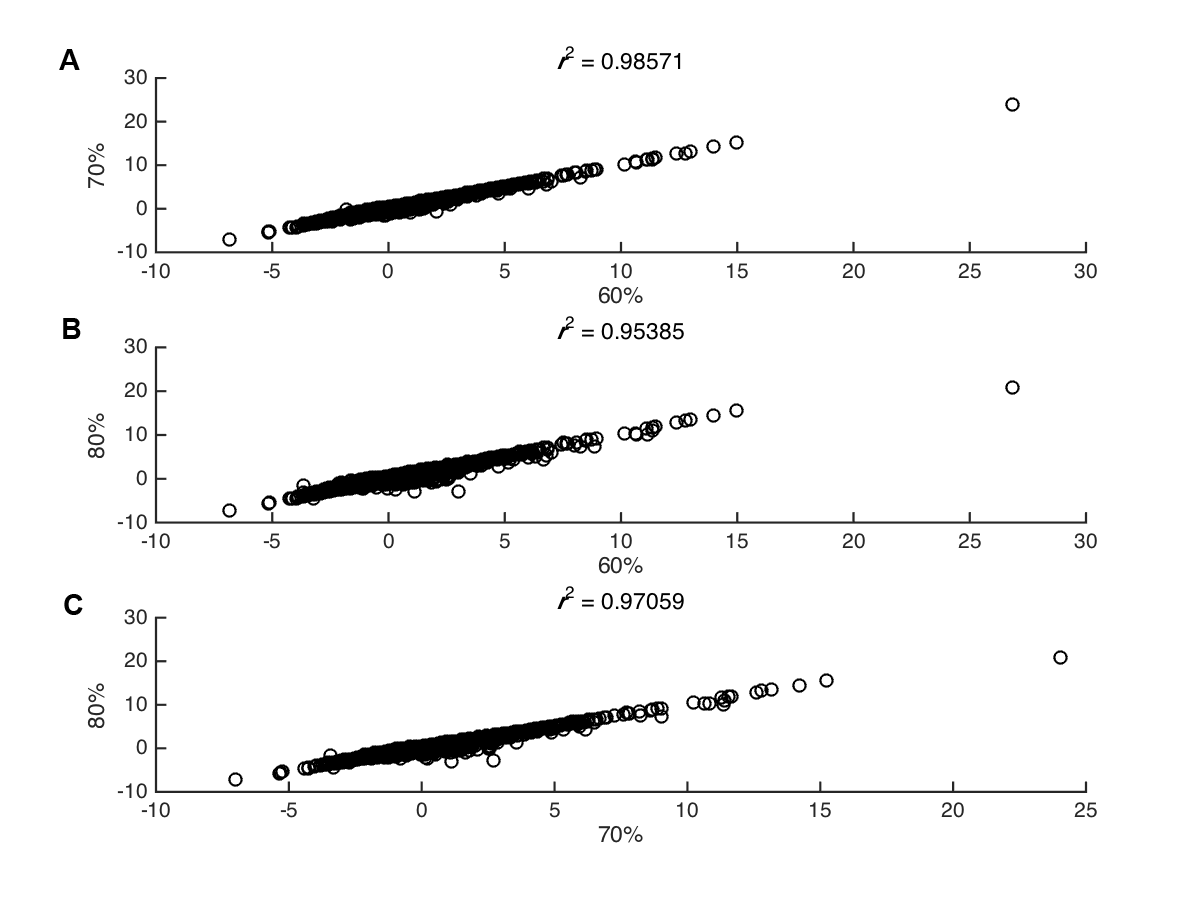

Supplement: S5 Fig — Scatter plots between three different ncRVIS sets, each generated with a different threshold for the percentage of samples with 10-fold coverage required at a given site for that site to be included. (A) compares the correlation between the choice of a 70% or 60% cut-off (r2 = 0.99); (B) compares the correlation between the choice of a 80% or 60% cut-off (r2 = 0.95); and (C) compares the correlation between the choice of a 80% or 70% cut-off (r2 = 0.97). These plots show that ncRVIS is not highly sensitive to varying this threshold. (TIFF) [file pgen.1005492.s005.tiff]
