# Supplementary material for: The Intolerance of Regulatory Sequence to Genetic Variation Predicts Gene Dosage Sensitivity
Source: PLoS Genet. 2015 Sep 2;11(9):e1005492. doi: 10.1371/journal.pgen.1005492 (PMC4557908; doi:10.1371/journal.pgen.1005492)
Supplement: S1 Table — Cohort of whole-genome sequenced samples used to construct the ncRVIS and RVIS-CHGV scores. (DOCX) [file pgen.1005492.s006.docx]

| **Ascertained phenotype** | **Number of genomes** |
| --- | --- |
| Hemophilia | 162 |
| Schizophrenia | 108 |
| HIV Controller | 99 |
| Chronic HIV Infection | 97 |
| Healthy Parent/Control | 76 |
| Amyotrophic Lateral Sclerosis (ALS) | 48 |
| Epilepsy / Autism | 36 |
| Centenarian | 19 |
| Long-Term HIV Non-Progressor | 15 |
| Other | 15 |
| Rapid HIV Progressor | 5 |
| APOE44 AD | 5 |
| Immunodeficiency | 5 |
