# Supplementary material for: The Intolerance of Regulatory Sequence to Genetic Variation Predicts Gene Dosage Sensitivity
Source: PLoS Genet. 2015 Sep 2;11(9):e1005492. doi: 10.1371/journal.pgen.1005492 (PMC4557908; doi:10.1371/journal.pgen.1005492)
Supplement: S2 Table — Based on 16,275 consensus coding sequence (CCDS) genes with assessable scores across all four RVIS formulations. Reflecting 96% of the CCDS release 9 genes scored in Petrovski et al (2013). Details on adopted gene-lists can be found in Petrovski et al (2013). RVIS: Based on the ESP6500 dataset. X = observed protein-coding variants, Y = common (>0.1% minor allele frequency) functional variants [15]. RVIS-mut: Based on the ESP6500 dataset. X = Genic mutation rate calculated via the tri-mer mutation matrix, Y = common (>0.1% minor allele frequency) functional variants. RVIS-YALL: Based on the ESP6500 dataset. X = Genic mutation rate calculated via the tri-mer mutation matrix, Y = common (>0.1% minor allele frequency) protein-coding variants of all effects (including synonymous variants). RVIS-CHGV: Based on an internally sequenced cohort of 690 whole-genome sequenced samples. X = Genic mutation rate calculated via the tri-mer mutation matrix, Y = common (>1% minor allele frequency) functional variants. To obtain the presented levels of significance, we used a logistic regression model to regress the presence or absence of a gene, within the corresponding gene list, on each of the genic scores. The [95% CI] for the AUC estimates are provided for each cell. Scatter plots for the pairs of scores are available in S1 Fig. (DOCX) [file pgen.1005492.s007.docx]

|  | **OMIM disease** | **Recessive** | **HI** | **dominant negative** | **HI and de novo** | **Essential Gene List** | Pearson’s *r* (*r*^2^) to RVIS (6503) |
| --- | --- | --- | --- | --- | --- | --- | --- |
| **Number of genes** | 2,067 | 798 | 165 | 352 | 99 | 2,206 | - |
| **RVIS (6503)** | **8.8x10^-26^**  AUC=0.577  [0.56-0.59] | **6.9x10^-5^**  AUC=0.551  [0.53-0.57] | **5.3x10^-31^**  AUC=0.735  [0.70-0.77] | **1.7x10^-23^**  AUC=0.650  [0.62-0.68] | **2.8x10^-28^**  AUC=0.786  [0.75-0.83] | **1.0x10^-110^**  AUC=0.656  [0.64-0.67] | **1 (1)** |
| **RVIS-mut (6503)** | **2.0x10^-8^**  AUC=0.548  [0.53-0.56] | **0.11**  AUC=0.504  [0.48-0.52] | **2.6x10^-30^**  AUC=0.749  [0.71-0.79] | **5.0x10^-18^**  AUC=0.644  [0.61-0.67] | **1.0x10^-28^**  AUC=0.803  [0.76-0.85] | **1.8x10^-114^**  AUC=0.676  [0.66-0.69] | **0.91 (0.83)** |
| **RVIS-YALL (6503)** | **4.0x10^-3^**  AUC=0.526  [0.51-0.54] | **0.01**  AUC=0.513  [0.49-0.53] | **4.3x10^-23^**  AUC=0.701  [0.66-0.74] | **5.5x10^-12^**  AUC=0.607  [0.58-0.64] | **2.9x10^-24^**  AUC=0.752  [0.70-0.80] | **2.3x10^-85^**  AUC=0.649  [0.64-0.66] | **0.77 (0.59)** |
| **RVIS-CHGV (690)** | **4.6x10^-10^**  AUC=0.549  [0.54-0.56] | **0.82**  AUC=0.516  [0.49-0.54] | **3.4x10^-29^**  AUC=0.724  [0.68-0.77] | **3.9x10^-17^**  AUC=0.634  [0.60-0.66] | **1.4x10^-27^**  AUC=0.765  [0.72-0.81] | **1.8x10^-97^**  AUC=0.662  [0.65-0.67] | **0.80 (0.63)** |

**S2 Table: Comparisons of deviatio­ns from RVIS**

Based on 16,275 consensus coding sequence (CCDS) genes with assessable scores across all four RVIS formulations. Reflecting 96% of the CCDS release 9 genes scored in Petrovski et al (2013). Details on adopted gene-lists can be found in Petrovski et al (2013).

**RVIS:** Based on the ESP6500 dataset. X = observed protein-coding variants, Y = common (>0.1% minor allele frequency) functional variants [[15](#_ENREF_15)]. **RVIS-mut:** Based on the ESP6500 dataset. X = Genic mutation rate calculated via the tri-mer mutation matrix, Y = common (>0.1% minor allele frequency) functional variants. **RVIS-YALL:** Based on the ESP6500 dataset. X = Genic mutation rate calculated via the tri-mer mutation matrix, Y = common (>0.1% minor allele frequency) protein-coding variants of all effects (including synonymous variants). **RVIS-CHGV:** Based on an internally sequenced cohort of 690 whole-genome sequenced samples. X = Genic mutation rate calculated via the tri-mer mutation matrix, Y = common (>1% minor allele frequency) functional variants.

To obtain the presented levels of significance, we used a logistic regression model to regress the presence or absence of a gene, within the corresponding gene list, on each of the genic scores. The [95% CI] for the AUC estimates are provided for each cell. Correlation plots for the pairs of scores are available in Fig S1.
