# Supplementary material for: The Intolerance of Regulatory Sequence to Genetic Variation Predicts Gene Dosage Sensitivity
Source: PLoS Genet. 2015 Sep 2;11(9):e1005492. doi: 10.1371/journal.pgen.1005492 (PMC4557908; doi:10.1371/journal.pgen.1005492)
Supplement: S3 Table — Based on the intersecting 9,644 (57%) CCDS release 9 genes that had all the following RVIS “assessable” formulations: an original RVIS score (Petrovski et al 2013), an ncRVIS, a promoter ncRVIS, a 5’ UTR ncRVIS, and a 3’ UTR ncRVIS. To obtain the presented levels of significance, we used a logistic regression model to regress the presence or absence of a gene, within the corresponding gene list, on each of the genic scores. The [95% CI] for the AUC estimates are provided for each cell. Scatter plots for the pairs of scores are available in S1 Fig. (DOCX) [file pgen.1005492.s008.docx]

|  | **OMIM disease** | **HI** | **HI and *de novo*** | **Essential Gene List** | Pearson’s *r* (*r^2^*) to ncRVIS |
| --- | --- | --- | --- | --- | --- |
| **Number of genes** | 1,232 | 87 | 47 | 1,098 | - |
| **RVIS (6503)** | **1.3x10^-18^**  AUC=0.594  [0.58-0.61] | **2.9x10^-13^**  AUC=0.711  [0.66-0.76] | **1.1x10^-10^**  AUC=0.769  [0.71-0.82] | **1.1x10^-43^**  AUC=0.647  [0.63-0.66] | 0.21 (0.042) |
| **ncRVIS (690)** | **0.42**  AUC=0.506  [0.49-0.52] | **1.9x10^-5^**  AUC=0.610  [0.55-0.67] | **1.4x10^-6^**  AUC=0.637  [0.56-0.71] | **5.5x10^-13^**  AUC=0.581  [0.56-0.60] | **1 (1)** |
| **ncRVIS (promoter)** | **0.19**  AUC=0.510  [0.49-0.53] | **0.024**  AUC=0.563  [0.50-0.62] | **0.064**  AUC=0.557  [0.48-0.63] | **3.1x10^-4^**  AUC=0.548  [0.53-0.57] | **0.50 (0.247)** |
| **ncRVIS (5’UTR)** | **0.025**  AUC=0.511  [0.49-0.53] | **0.012**  AUC=0.560  [0.50-0.62] | **0.088**  AUC=0.554  [0.46-0.64] | **5.1x10^-6^**  AUC=0.546  [0.53-0.56] | **0.44 (0.197)** |
| **ncRVIS (3’UTR)** | **0.98**  AUC=0.506  [0.49-0.52] | **1.2x10^-4^**  AUC=0.587  [0.53-0.65] | **1.4x10^-7^**  AUC=0.631  [0.55-0.71] | **2.0x10^-8^**  AUC=0.568  [0.55-0.59] | **0.89 (0.790)** |

**S3 Table.** Based on the intersecting 9,644 (57%) CCDS release 9 genes that had all the following RVIS “assessable” formulations: an original RVIS score (Petrovski et al 2013), a ncRVIS, a promoter ncRVIS, a 5’ UTR ncRVIS, and a 3’ UTR ncRVIS.

To obtain the presented levels of significance, we used a logistic regression model to regress the presence or absence of a gene, within the corresponding gene list, on each of the genic scores. The [95% CI] for the AUC estimates are provided for each cell. Correlation plots for the pairs of scores are available in Fig S1.
