# Supplementary material for: The Intolerance of Regulatory Sequence to Genetic Variation Predicts Gene Dosage Sensitivity
Source: PLoS Genet. 2015 Sep 2;11(9):e1005492. doi: 10.1371/journal.pgen.1005492 (PMC4557908; doi:10.1371/journal.pgen.1005492)
Supplement: S4 Table — This table contains the estimates achieved by six genic features in their ability to predict ClinGen’s dosage sensitive genes from the remaining exome background. See accompanying Fig 2. (DOCX) [file pgen.1005492.s009.docx]

| **Score** | **Effect Size** | **Standard Error** | **P-Value** |
| --- | --- | --- | --- |
| **ncRVIS** | -0.30809 | 0.09160 | 0.00077 |
| **ncGERP** | 0.57218 | 0.08233 | 3.66x10^-12^ |
| **RVIS-CHGV** | -0.66828 | 0.10011 | 2.47x10^-11^ |
| **pcGERP** | 0.27303 | 0.10181 | 0.00732 |
| **ncCADD** | -0.11929 | 0.04028 | 0.00306 |
| **ncGWAVA** | 3.39838 | 0.86225 | 8.10x10^-5^ |

**S4 Table. Joint logistic regression model to predict ClinGen’s dosage sensitive genes.**

This table contains the estimates achieved by six genic features in their ability to predict ClinGen’s dosage sensitive genes from the remaining exome background. See accompanying Figure 2.
