# Supplementary material for: The Intolerance of Regulatory Sequence to Genetic Variation Predicts Gene Dosage Sensitivity
Source: PLoS Genet. 2015 Sep 2;11(9):e1005492. doi: 10.1371/journal.pgen.1005492 (PMC4557908; doi:10.1371/journal.pgen.1005492)
Supplement: S5 Table — This table contains the loss-of-function de novo mutations found among a collection of neuropsychiatric disorder ascertained patients, that both occur in genes that are loss-of-function deficient and have a Euclidean distance from (0,0) ≤ 0.4. (DOCX) [file pgen.1005492.s010.docx]

| **PMID** | **Disease** | **Trio ID** | **RVIS%** | **Variant Effect** | **HGNC** | **%RVIS CHGV** | **%ncRVIS** | **RVIS-sum** | **Expected % LoF (mutRate)** | **Expected LoF in EVS (Number)** | **Observed LoF (EVS)** | **Euclidean Distance** | **Binomial Exact 1-tail (BH [FDR])** |
| --- | --- | --- | --- | --- | --- | --- | --- | --- | --- | --- | --- | --- | --- |
| 25363768 | Autism | 12044 | 0.625 | stop gain | *WDFY3* | 0.086 | 0.817 | 0.904 | 0.109 | 24.604 | 1 | 0.008 | 2.6x10^-8^ |
| 22542183 | Autism | 13094 | 0.625 | stop gain | *WDFY3* | 0.086 | 0.817 | 0.904 | 0.109 | 24.604 | 1 | 0.008 | 2.6x10^-8^ |
| 25363768 | Autism | 13813 | 4.052 | indel | *MED13* | 0.777 | 0.756 | 1.533 | 0.105 | 18.166 | 0 | 0.011 | 5.4x10^-7^ |
| 25363768 | Autism | 11145 | 0.407 | indel | *KMT2A* | 0.081 | 1.592 | 1.672 | 0.099 | 24.706 | 1 | 0.016 | 2.6x10^-8^ |
| 25363768 | Autism | 11025 | 2.465 | indel | *BTAF1* | 1.128 | 2.937 | 4.065 | 0.119 | 14.223 | 0 | 0.031 | 1.6x10^-5^ |
| 23934111 | EE | isnd31120cq1 | 9.324 | stop gain | *RALGAPB* | 2.008 | 2.489 | 4.497 | 0.103 | 10.011 | 1 | 0.032 | 4.6x10^-3^ |
| 25363768 | Autism | 12093 | 0.448 | stop gain | *DOT1L* | 0.736 | 3.189 | 3.926 | 0.057 | 10.693 | 0 | 0.033 | 4.7x10^-4^ |
| 22495311 | Autism | 09C98975 | 6.293 | stop gain | *ZNF292* | 2.330 | 3.441 | 5.772 | 0.098 | 23.422 | 1 | 0.042 | 8.4x10^-8^ |
| 23033978 | severe ID | DUTCH22 | 6.293 | indel | *ZNF292* | 2.330 | 3.441 | 5.772 | 0.098 | 23.422 | 1 | 0.042 | 8.4x10^-8^ |
| 25363768 | Autism | 13097 | 20.022 | stop gain | *ARHGAP5* | 2.486 | 4.203 | 6.689 | 0.096 | 11.685 | 0 | 0.049 | 1.7x10^-4^ |
| 22542183 | Autism | 12645 | 0.413 | stop gain | *ANK2* | 0.558 | 5.033 | 5.591 | 0.094 | 34.905 | 0 | 0.051 | 8.2x10^-14^ |
| 25363768 | Autism | 13768 | 0.413 | indel | *ANK2* | 0.558 | 5.033 | 5.591 | 0.094 | 34.905 | 0 | 0.051 | 8.2x10^-14^ |
| 25363768 | Autism | 14256 | 0.413 | stop gain | *ANK2* | 0.558 | 5.033 | 5.591 | 0.094 | 34.905 | 0 | 0.051 | 8.2x10^-14^ |
| 25363768 | Autism | 12733 | 3.686 | indel | *BRD4* | 1.928 | 6.348 | 8.275 | 0.075 | 8.136 | 0 | 0.066 | 3.4x10^-3^ |
| 25363768 | Autism | 14617 | 5.709 | indel | *RALGAPA1* | 2.986 | 6.071 | 9.058 | 0.119 | 15.510 | 0 | 0.068 | 5.1x10^-6^ |
| 23042115 | SCZ | trio_026 | 3.114 | indel | *RB1CC1* | 5.455 | 4.738 | 10.192 | 0.119 | 16.181 | 0 | 0.072 | 2.9x10^-6^ |
| 25363768 | Autism | 14165 | 6.564 | indel | *SPAG9* | 6.018 | 4.511 | 10.529 | 0.104 | 10.852 | 0 | 0.075 | 3.3x10^-4^ |
| 24463507 | SCZ | 3096-1 | 1.014 | stop gain | *NIPBL* | 0.460 | 9.101 | 9.561 | 0.126 | 20.646 | 0 | 0.091 | 4.4x10^-8^ |
| 22495306 | Autism | 11114 | 1.769 | stop gain | *SCN2A* | 1.398 | 9.199 | 10.597 | 0.100 | 12.214 | 1 | 0.093 | 9.0x10^-4^ |
| 22495306 | Autism | 11892 | 1.769 | stop gain | *SCN2A* | 1.398 | 9.199 | 10.597 | 0.100 | 12.214 | 1 | 0.093 | 9.0x10^-4^ |
| 23033978 | severe ID | DUTCH22 | 1.769 | stop gain | *SCN2A* | 1.398 | 9.199 | 10.597 | 0.100 | 12.214 | 1 | 0.093 | 9.0x10^-4^ |
| 23020937 | severe ID | ER8490 | 1.769 | indel | *SCN2A* | 1.398 | 9.199 | 10.597 | 0.100 | 12.214 | 1 | 0.093 | 9.0x10^-4^ |
| 24463507 | SCZ | 1317-1 | 1.769 | splice acceptor | *SCN2A* | 1.398 | 9.199 | 10.597 | 0.100 | 12.214 | 1 | 0.093 | 9.0x10^-4^ |
| 23020937 | severe ID | MS111684 | 1.769 | indel | *SCN2A* | 1.398 | 9.199 | 10.597 | 0.100 | 12.214 | 1 | 0.093 | 9.0x10^-4^ |
| 25363768 | Autism | 13760 | 3.332 | indel | *RANBP2* | 0.316 | 9.500 | 9.817 | 0.103 | 31.026 | 1 | 0.095 | 7.8x10^-11^ |
| 25363768 | Autism | 14133 | 12.096 | indel | *PHF3* | 4.954 | 9.027 | 13.981 | 0.106 | 18.862 | 0 | 0.103 | 2.9x10^-7^ |
| 22495309 | Autism | 12346 | 1.516 | indel | *MBD5* | 2.158 | 10.502 | 12.660 | 0.086 | 10.961 | 0 | 0.107 | 3.3x10^-4^ |
| 25363768 | Autism | 14012 | 0.572 | indel | *UBR5* | 0.242 | 11.860 | 12.102 | 0.121 | 18.426 | 0 | 0.119 | 3.7x10^-7^ |
| 25363768 | Autism | 12600 | 4.777 | indel | *ZC3H4* | 3.297 | 11.645 | 14.942 | 0.074 | 10.015 | 1 | 0.121 | 5.0x10^-3^ |
| 25363768 | Autism | 12600 | 4.777 | indel | *ZC3H4* | 3.297 | 11.645 | 14.942 | 0.074 | 10.015 | 1 | 0.121 | 5.0x10^-3^ |
| 23020937 | severe ID | ER14209 | 12.597 | stop gain | *SETD5* | 4.114 | 11.989 | 16.103 | 0.094 | 10.501 | 1 | 0.127 | 3.3x10^-3^ |
| 22495311 | Autism | 10C104461 | 10.922 | stop gain | *ITGA5* | 7.365 | 12.278 | 19.643 | 0.103 | 10.605 | 1 | 0.143 | 3.0x10^-3^ |
| 25363768 | Autism | 12950 | 19.999 | indel | *UBN2* | 10.046 | 12.628 | 22.674 | 0.091 | 10.218 | 1 | 0.161 | 4.1x10^-3^ |
| 25363768 | Autism | 14687 | 10.728 | stop gain | *INTS6* | 10.150 | 13.108 | 23.257 | 0.118 | 6.980 | 0 | 0.166 | 7.3x10^-3^ |
| 25363768 | Autism | 12969 | 0.69 | indel | *MED13L* | 0.409 | 16.672 | 17.080 | 0.090 | 15.984 | 1 | 0.167 | 4.8x10^-5^ |
| 25363768 | Autism | 14416 | 0.69 | stop gain | *MED13L* | 0.409 | 16.672 | 17.080 | 0.090 | 15.984 | 1 | 0.167 | 4.8x10^-5^ |
| 25363768 | Autism | 14093 | 7.484 | indel | *LARP4B* | 6.283 | 17.919 | 24.202 | 0.088 | 6.970 | 0 | 0.19 | 8.0x10^-3^ |
| 25363768 | Autism | 13344 | 0.33 | stop gain | *NOTCH1* | 0.270 | 19.554 | 19.824 | 0.055 | 19.034 | 0 | 0.196 | 3.8x10^-7^ |
| 25363768 | Autism | 13006 | 7.714 | indel | *STAG1* | 8.475 | 18.349 | 26.825 | 0.128 | 8.572 | 0 | 0.202 | 2.0x10^-3^ |
| 25363768 | Autism | 12952 | 3.904 | indel | *KMT2E* | 5.903 | 20.082 | 25.986 | 0.099 | 16.250 | 1 | 0.209 | 3.7x10^-5^ |
| 25363768 | Autism | 14299 | 3.904 | indel | *KMT2E* | 5.903 | 20.082 | 25.986 | 0.099 | 16.250 | 1 | 0.209 | 3.7x10^-5^ |
| 25363768 | Autism | 13614 | 2.371 | stop gain | *CHD2* | 3.855 | 23.542 | 27.397 | 0.115 | 13.876 | 0 | 0.239 | 2.3x10^-5^ |
| 25363768 | Autism | 13618 | 2.371 | indel | *CHD2* | 3.855 | 23.542 | 27.397 | 0.115 | 13.876 | 0 | 0.239 | 2.3x10^-5^ |
| 25363768 | Autism | 13818 | 2.371 | indel | *CHD2* | 3.855 | 23.542 | 27.397 | 0.115 | 13.876 | 0 | 0.239 | 2.3x10^-5^ |
| 23934111 | EE | lgsnd29528ih1 | 2.371 | splice donor | *CHD2* | 3.855 | 23.542 | 27.397 | 0.115 | 13.876 | 0 | 0.239 | 2.3x10^-5^ |
| 25363768 | Autism | 13678 | 2.223 | stop gain | *ASH1L* | 0.575 | 24.501 | 25.076 | 0.107 | 21.501 | 1 | 0.245 | 4.0x10^-7^ |
| 23934111 | EE | lgsnd24053gj1 | 4.028 | stop gain | *SCN1A* | 1.341 | 24.630 | 25.970 | 0.105 | 15.794 | 0 | 0.247 | 4.5x10^-6^ |
| 23934111 | EE | lgsnd30216iy1 | 4.028 | splice donor | *SCN1A* | 1.341 | 24.630 | 25.970 | 0.105 | 15.794 | 0 | 0.247 | 4.5x10^-6^ |
| 23934111 | EE | lgsnd32727kd1 | 4.028 | splice donor | *SCN1A* | 1.341 | 24.630 | 25.970 | 0.105 | 15.794 | 0 | 0.247 | 4.5x10^-6^ |
| 23934111 | EE | lgsnd34816ku1 | 4.028 | indel | *SCN1A* | 1.341 | 24.630 | 25.970 | 0.105 | 15.794 | 0 | 0.247 | 4.5x10^-6^ |
| 22542183 | Autism | 12764 | 5.762 | stop gain | *NCKAP1* | 5.173 | 24.931 | 30.103 | 0.123 | 7.779 | 0 | 0.255 | 3.8x10^-3^ |
| 25363768 | Autism | 14030 | 5.762 | indel | *NCKAP1* | 5.173 | 24.931 | 30.103 | 0.123 | 7.779 | 0 | 0.255 | 3.8x10^-3^ |
| 23934111 | EE | lgsnd33762kq1 | 16.071 | indel | *DNAJC6* | 14.217 | 21.244 | 35.461 | 0.108 | 11.294 | 0 | 0.256 | 2.2x10^-4^ |
| 25363768 | Autism | 11654 | 1.18 | splice acceptor | *CHD8* | 0.380 | 27.678 | 28.057 | 0.100 | 16.937 | 0 | 0.277 | 1.7x10^-6^ |
| 22495309 | Autism | 12752 | 1.18 | indel | *CHD8* | 0.380 | 27.678 | 28.057 | 0.100 | 16.937 | 0 | 0.277 | 1.7x10^-6^ |
| 25363768 | Autism | 12991 | 1.18 | indel | *CHD8* | 0.380 | 27.678 | 28.057 | 0.100 | 16.937 | 0 | 0.277 | 1.7x10^-6^ |
| 22495309 | Autism | 13844 | 1.18 | stop gain | *CHD8* | 0.380 | 27.678 | 28.057 | 0.100 | 16.937 | 0 | 0.277 | 1.7x10^-6^ |
| 25363768 | Autism | 13900 | 1.18 | indel | *CHD8* | 0.380 | 27.678 | 28.057 | 0.100 | 16.937 | 0 | 0.277 | 1.7x10^-6^ |
| 25363768 | Autism | 14016 | 1.18 | stop gain | *CHD8* | 0.380 | 27.678 | 28.057 | 0.100 | 16.937 | 0 | 0.277 | 1.7x10^-6^ |
| 25363768 | Autism | 14233 | 1.18 | indel | *CHD8* | 0.380 | 27.678 | 28.057 | 0.100 | 16.937 | 0 | 0.277 | 1.7x10^-6^ |
| 25363768 | Autism | 14533 | 6.334 | stop gain | *GIGYF2* | 13.049 | 26.092 | 39.142 | 0.141 | 16.319 | 0 | 0.292 | 2.1x10^-6^ |
| 22542183 | Autism | 12501 | 2.247 | stop gain | *NRXN1* | 0.662 | 29.595 | 30.257 | 0.071 | 9.517 | 0 | 0.296 | 1.1x10^-3^ |
| 24463507 | SCZ | 3010-1 | 4.518 | indel | *KIAA1429* | 1.243 | 29.773 | 31.016 | 0.108 | 14.960 | 1 | 0.298 | 9.9x10^-5^ |
| 25363768 | Autism | 13398 | 3.409 | indel | *POGZ* | 9.925 | 28.999 | 38.924 | 0.099 | 9.912 | 1 | 0.307 | 4.9x10^-4^ |
| 25363768 | Autism | 13627 | 3.409 | stop gain | *POGZ* | 9.925 | 28.999 | 38.924 | 0.099 | 9.912 | 1 | 0.307 | 4.9x10^-4^ |
| 22495311 | Autism | 10C102646 | 3.409 | indel | *POGZ* | 9.925 | 28.999 | 38.924 | 0.099 | 9.912 | 1 | 0.307 | 4.9x10^-4^ |
| 24463507 | SCZ | 3103-1 | 3.409 | indel | *POGZ* | 9.925 | 28.999 | 38.924 | 0.099 | 9.912 | 1 | 0.307 | 4.9x10^-4^ |
| 22495306 | Autism | 13128 | 3.863 | stop gain | *EPHB2* | 1.870 | 32.766 | 34.636 | 0.061 | 7.262 | 0 | 0.328 | 6.9x10^-3^ |
| 22542183 | Autism | 12221 | 17.31 | stop gain | *FAM91A1* | 26.323 | 23.714 | 50.037 | 0.118 | 6.971 | 0 | 0.354 | 7.3x10^-3^ |
| 25363768 | Autism | 12653 | 6.894 | indel | *DLL1* | 6.093 | 39.261 | 45.355 | 0.067 | 6.864 | 0 | 0.397 | 9.2x10^-3^ |

**S5 Table:** This table contains the loss-of-function *de novo* mutations found among a collection of neuropsychiatric disorder ascertained patients, that both occur in genes that are loss-of-function deficient and have a Euclidean distance from (0,0) ≤ 0.4.

SCZ = Schizophrenia; EE = Epileptic Encephalopathy; severe ID = Severe Intellectual Disability
